# A disulfide bond sculpts the CTNIP4 phytocytokine fold for recognition by the receptor kinase HSL3

**DOI:** 10.1038/s41477-026-02380-y

**Published:** 2026-09-17

**Authors:** Pedro Jiménez-Sandoval, Oliver Johanndrees, Simon Snoeck, Chitthavalli Y. Harshith, Moutasem Omary, Caroline Broyart, Jack Rhodes, Kyle W. Bender, Cyril Zipfel, Julia Santiago

**Affiliations:** 1 The Plant Signaling Mechanisms Laboratory, Department of Plant Molecular Biology, University of Lausanne, Lausanne, Switzerland; 2 Institute of Plant and Microbial Biology, Zurich-Basel Plant Science Center, University of Zurich, Zurich, Switzerland; 3 The Sainsbury Laboratory, University of East Anglia, Norwich Research Park, Norwich, UK; 4 Present Address: Lonza, Visp, Switzerland

**Keywords:** Plant signalling, Pattern recognition receptors in plants

## Abstract

Precise ligand recognition by closely related leucine-rich repeat receptor kinases (LRR-RKs) is essential for plants to coordinate immunity, development and environmental adaptation. Here we show how the LRR-RK HSL3/NUT specifically recognizes the folded, disulfide-stabilized CTNIP4/SCREW2 phytocytokine in *Arabidopsis*. Quantitative binding assays define a minimal CTNIP4 region required for high-affinity HSL3 interaction and signalling activation. A 2.12-Å crystal structure of the HSL3–CTNIP4 complex reveals a unique C-terminal receptor pocket that accommodates the peptide’s cyclic architecture through a combination of hydrophobic and polar contacts, a feature absent in the closely related HAE/HSL LRR-RKs. The cyclic CTNIP4 fold further establishes a largely hydrophobic interface that bridges HSL3 to the SERK co-receptor, forming a distinct activation surface. Together, these structural, biochemical and physiological insights uncover a previously unrecognized mechanism of CTNIP4 peptide perception and HSL3 receptor activation, highlighting how subtle architectural variations enable precise ligand selectivity among highly conserved plant receptor kinases.

## Abstract (web summary)

A disulfide bond sculpts a plant signalling peptide into a unique shape that enables selective receptor recognition and activation, revealing an unexpected mechanism by which plants achieve highly specific cell-to-cell communication.

## Main

Plant peptide hormones have emerged as central mediators of cell-to-cell communication, coordinating growth, development and environmental adaptation such as immune responses^1–14^. These signalling molecules are typically processed from larger precursor proteins and undergo diverse post-translational modifications including tyrosine sulfation, proline hydroxylation, glycosylation or the formation of disulfide bonds^3,6,7,11,14–19^. Such chemical modifications can critically influence peptide diffusion, stability and bioactivity, enabling precise recognition by their cognate cell-surface receptors^6,7,9^. To perceive an extensive repertoire of sequence-diverse signalling peptides, plants have co-evolved an equally diverse array of cell-surface localized receptors. Among these, leucine-rich repeat receptor kinases (LRR-RKs) represent the largest and most functionally versatile family^20^. Despite their structural similarity, individual LRR-RKs have diversified their extracellular binding pockets to prevent ligand cross-reactivity and ensure specific recognition of their bona fide peptide hormones^6^. Typically, LRR-RKs initiate a specific signalling response through the ligand-dependent recruitment of a shape-complementary co-receptor, forming an active receptor complex at the plasma membrane^7–9,20–22^.

Recently, we and others identified a new family of plant signalling peptides acting as phytocytokines, termed CTNIPs or SMALL PHYTOCYTOKINES REGULATING DEFENSE AND WATER LOSS (SCREWs), together with their cognate receptor, the LRR-RK HAESA-LIKE 3 (HSL3), also known as PLANT SCREW UNRESPONSIVE RECEPTOR (NUT)^1,4,23^. CTNIP/SCREW peptides contain a pair of conserved cysteine residues that are essential for their bioactivity and for recognition by HSL3/NUT^4^. Ligand binding promotes heteromerization of HSL3/NUT with members of the SOMATIC EMBRYOGENESIS RECEPTOR-LIKE KINASE (SERK) co-receptor family such as SERK3 (also named BRASSINOSTEROID INSENSITIVE 1-ASSOCIATED KINASE 1, BAK1), forming an active receptor complex that triggers stress-induced responses^1,4^. However, despite its homology to the HAESA (HAE) and HAESA-LIKE 1/2 (HSL1/2) receptors, HSL3 features a distinct ligand-binding pocket that lacks the conserved motifs required for INFLORESCENCE DEFICIENT IN ABSCISSION (IDA)/IDA-LIKE (IDL) peptide recognition^4,6,7^, suggesting that it uses a different mechanism to perceive CTNIP/SCREW peptides. Elucidating this unique mode of peptide recognition and receptor activation is the central focus of this study.

**Fig. 1:**
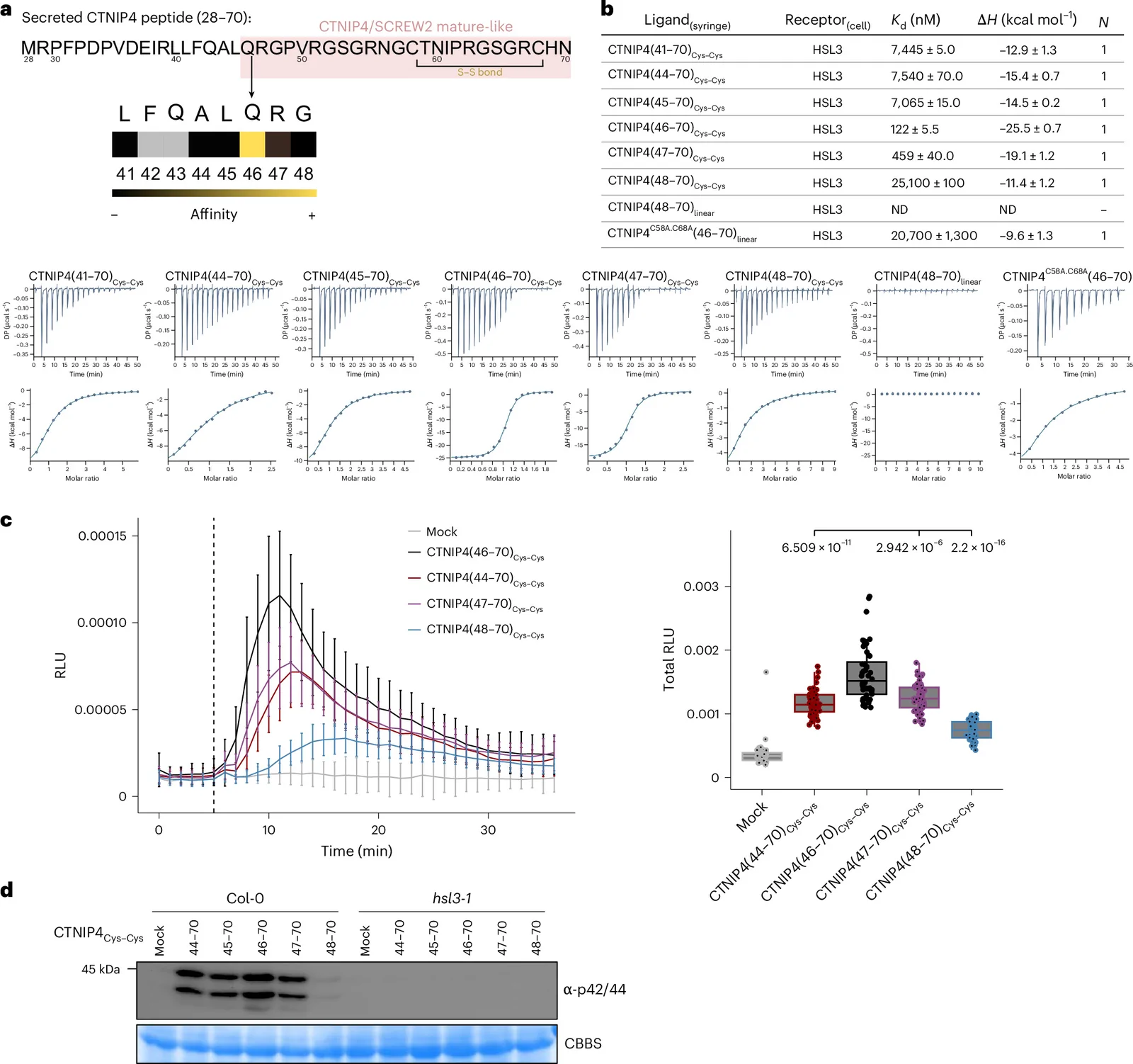
Mapping of a bioactive CTNIP4/SCREW2 peptide specifically recognized by the HSL3 receptor. Systematic biochemical analysis defines the minimal region of the CTNIP4/SCREW2 peptide required for high-affinity recognition by the HSL3 receptor. **a**, Schematic representation of the CTNIP4 secreted precursor. The N-terminal truncations used for biochemical analysis are indicated below. The minimal region required for high-affinity binding, including the conserved disulfide bond, is highlighted in pink. Binding affinities are represented using a colour scale corresponding to the inverse of the *K*_d_ value, with yellow indicating the highest affinity. Grey indicates peptide lengths that were not tested. **b**, ITC assays showing binding of HSL3 ectodomain to folded and linear CTNIP4 peptides of varying lengths. ITC table summarizes *K*_d_ (nM), stoichiometry (*N* = 1); ND, no binding detected. Data are presented as mean ± s.d. from at least two independent experiments. **c**, Ca^2+^ influx assays in relative luminescence units (RLUs) show that folded CTNIP4(46–70)_Cys–Cys_ elicits the highest response in wild-type Col-0^AEQ^ plants compared with other lengths. The peptide concentration used was 100 nM. Data represent mean ± s.d. (*n* = 48), and the vertical dashed line indicates the timing of the treatment. Considering the cumulative RLUs (3–30 min after treatment), the box plots indicate the median and the interquartile range (IQR), and the whiskers extend to the most extreme data points within 1.5 times the IQR. Significance was tested by performing two-sided non-parametric Wilcoxon–Mann–Whitney tests between CTNIP4(46–70)_Cys–Cys_ and CTNIP4 length variants. **d**, Immunoblot analysis of MAP kinase phosphorylation in response to folded 5-nM CTNIP4 peptides of different lengths for 20 min, confirming that functional activity correlates with high-affinity receptor binding. Data shown are from one representative experiment out of three that yielded similar results. CBBS, Coomassie Brilliant Blue staining; DP, differential power. Source data

**Extended Data Fig. 1:**
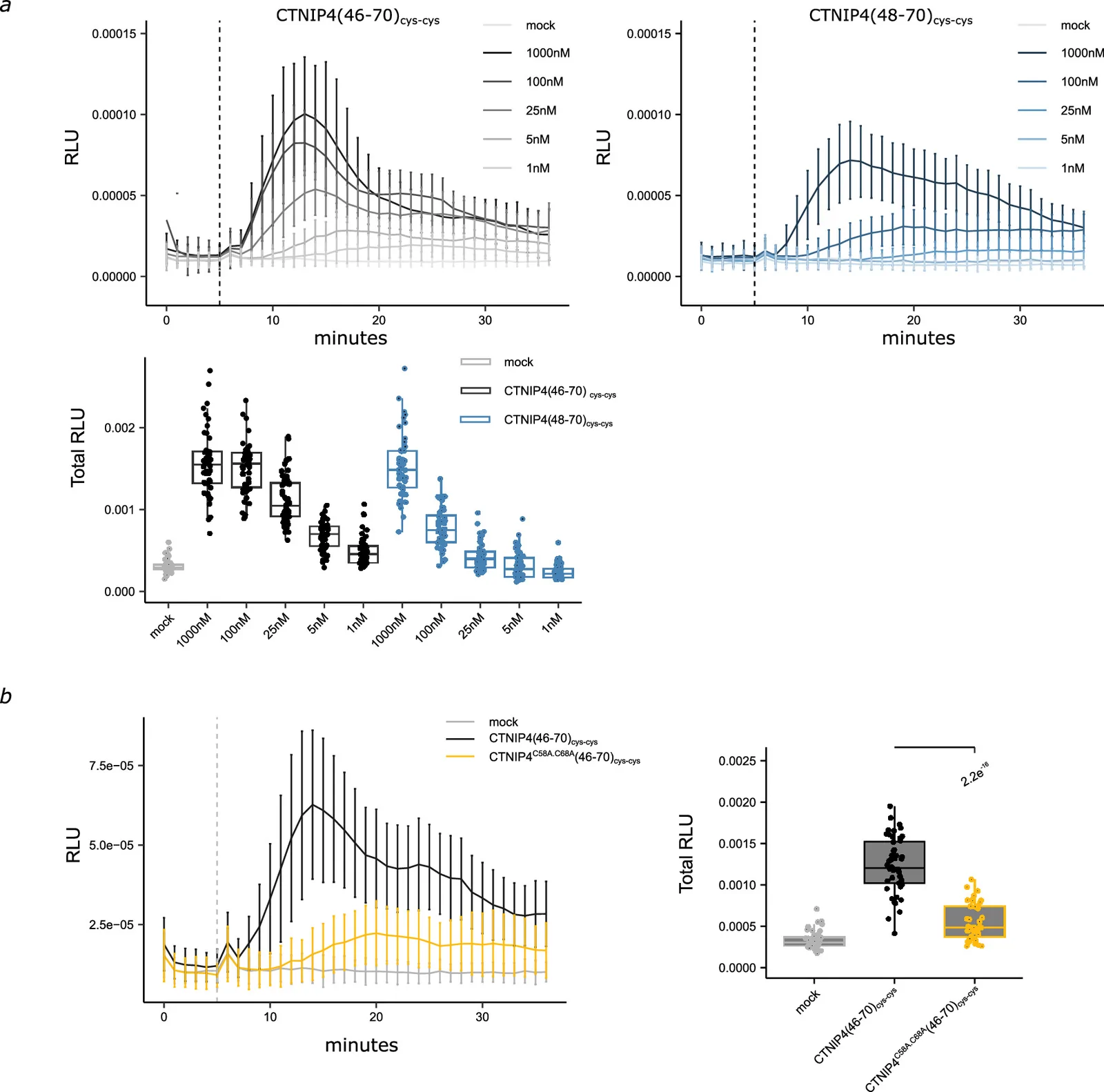
Bioactvivity of CTNIP4/SCREW2 peptide variants. Calcium responses are shown as relative luminescence units (RLUs). Data represents mean ± SD (n = 48). Vertical dotted lines indicate the time of peptide application. Box plots show cumulative RLUs from 3–30 min post-treatment, with medians and interquartile ranges (IQR); whiskers extend to 1.5× IQR. **a**, The fully coordinated CTNIP4 peptide elicits calcium signaling at lower concentrations. Comparative Ca^2+^ influx responses in wild-type Col-0^AEQ^ plants treated with the highly coordinated CTNIP4(46–70)_Cys-Cys_ peptide or the truncated CTNIP4(48–70)_Cys-Cys_ variant. Peptide concentrations are indicated in the figure. **b**, Calcium responses to CTNIP4(46–70)_Cys-Cys_ and the linear, non-cyclized mutant CTNIP4^C58A.C68A^(46–70) at 25 nM. Statistical significance was assessed using two-sided Wilcoxon-Mann-Whitney tests comparing CTNIP4(46–70)_Cys-Cys_ with length and cysteine variants. Source data

Building on our previous identification of CTNIP peptides and the report of micromolar range binding affinities^4^, we next sought to define a minimal CTNIP region required for high-affinity (nanomolar range) recognition by the HSL3 receptor. To this end, we synthetized multiple longer or shorter N-terminal versions of CTNIP4 (corresponding to SCREW2) and assessed binding of the resulting peptide variants to the HSL3 ectodomain using isothermal titration calorimetry (ITC) (Fig. 1a,b and Supplementary Figs. 1 and 2a). Given the essential role of the conserved cysteine residues for receptor interaction^4^, all variants were synthesized with a disulfide bond linking Cys58 and Cys68 to stabilize the folded conformation and peptide homogeneity (Methods). ITC analyses revealed that CTNIP4(46–70)_Cys–Cys_ exhibited the highest binding affinity to HSL3, defining the peptide length most effectively coordinated by the receptor-binding pocket. The interaction is enthalpy driven, indicating that peptide–receptor recognition is dominated by specific polar and hydrogen-bonding interactions rather than by hydrophobic effects (Supplementary Fig. 2b). By contrast, longer CTNIP4 versions displayed up to a 60-fold reduction in affinity, suggesting that additional N-terminal residues may hinder receptor recognition. Stepwise truncation further refined this interaction: removal of one residue CTNIP4(47–70)_Cys–Cys_ resulted in an approximately fourfold reduction in binding, while deletion of two residues, CTNIP4(48–70)_Cys–Cys_, led to a dramatic ~200-fold loss of affinity (Fig. 1b and Supplementary Fig. 2a). These results identify Gln46 and Arg47 as key anchoring residues required for optimal receptor–peptide coordination. Consistent with these observations, the linear (non-cyclized) CTNIP4^C58A.C68A^(46–70) and the shorter and non-folded CTNIP4(48–70)_linear_ peptides, displayed either severely impaired (~200-fold) or non-detectable binding, respectively. These results highlight the critical contribution of both the N-terminal anchoring residues and the disulfide-stabilized cyclic conformation to high affinity and thermodynamically favourable receptor–peptide association (Fig. 1b and Supplementary Fig. 2a,b). To assess whether binding affinities correlate with biological activity, we next tested the CTNIP4 variants for their ability to trigger downstream signalling responses in planta. Measurements of cytosolic Ca^2+^ influx and MAP kinase phosphorylation closely mirrored the in vitro binding profiles, with the CTNIP4(46–70)_Cys–Cys_ peptide, which exhibits the highest binding affinity to HSL3, inducing robust signalling responses (Fig. 1c,d, Extended Data Fig. 1a and Supplementary Fig. 2c). By contrast, the linear CTNIP4^C58A.C68A^(46–70) peptide showed severely reduced bioactivity, consistent with its markedly impaired binding affinity observed in the biochemical assays (Fig. 1b and Extended Data Fig. 1b). Together, these results delineate the core structural features and sequence determinants of CTNIP4 required for receptor recognition and signalling activation.

**Fig. 2:**
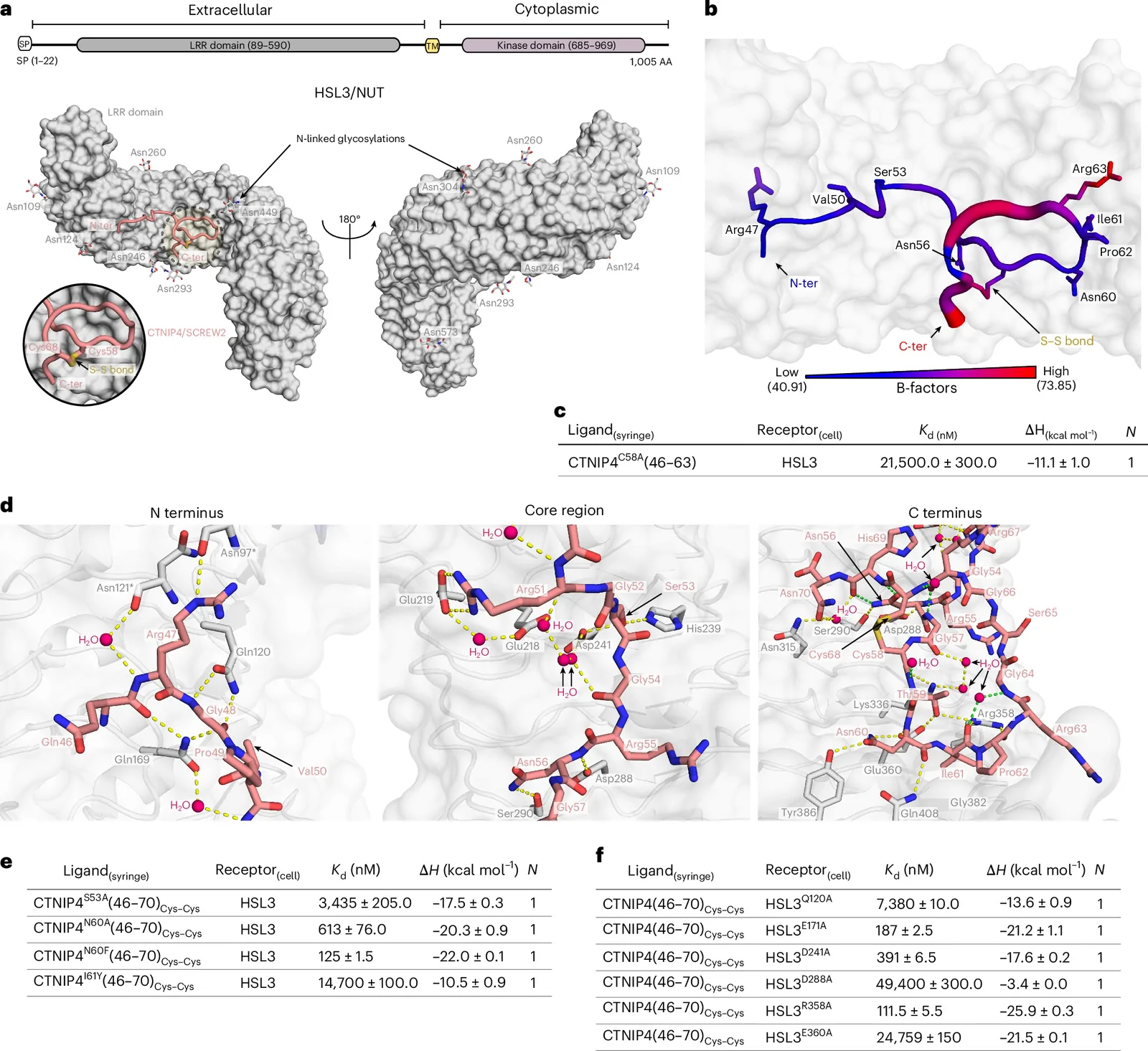
HSL3 pocket recognizes the unique disulfide-stabilized fold of CTNIP4. **a**, Domain architecture of HSL3, comprising a signal peptide (SP), an LRR ectodomain, a single transmembrane helix (TM) and a cytoplasmic kinase domain. Surface representation of HSL3 ectodomain (grey) bound to CTNIP4 (pink cartoon) shows the peptide adopting a cyclic conformation stabilized by an intramolecular disulfide bond. The disulfide bond is shown in yellow, and N-linked glycans are displayed as grey sticks. AA, amino acid. **b**, Temperature-factor (B-factor) representation of CTNIP4 within the HSL3 binding pocket, illustrating receptor–peptide coordination. Regions of low B-factors correspond to tightly coordinated peptide segments stabilized by receptor contacts. **c**, The disulfide-locked cyclic C-terminal region of CTNIP4 is essential for high-affinity HSL3 binding. ITC of the truncated CTNIP4(46–63) linear peptide versus HSL3. **d**, Detailed view of CTNIP4 coordination along the HSL3 binding pocket. HSL3 is shown in grey, CTNIP4 in pink sticks and water molecules in hot-pink spheres. Hydrogen bonds between receptor and peptide are depicted as dashed yellow lines, and intramolecular polar contacts stabilizing the cyclic peptide fold are shown in dashed green lines. **e**,**f**, ITC analyses of engineered CTNIP4 variants binding to wild-type HSL3. **f**, ITC analyses of engineered HSL3 receptor variants binding to wild-type CTNIP4. Together, these variants reveal key residues contributing to receptor-peptide coordination. Tables summarize dissociation constants (*K*_d_, nM), stoichiometry (*N* = 1) and mean ± s.d. from at least two independent experiments.

**Fig. 3:**
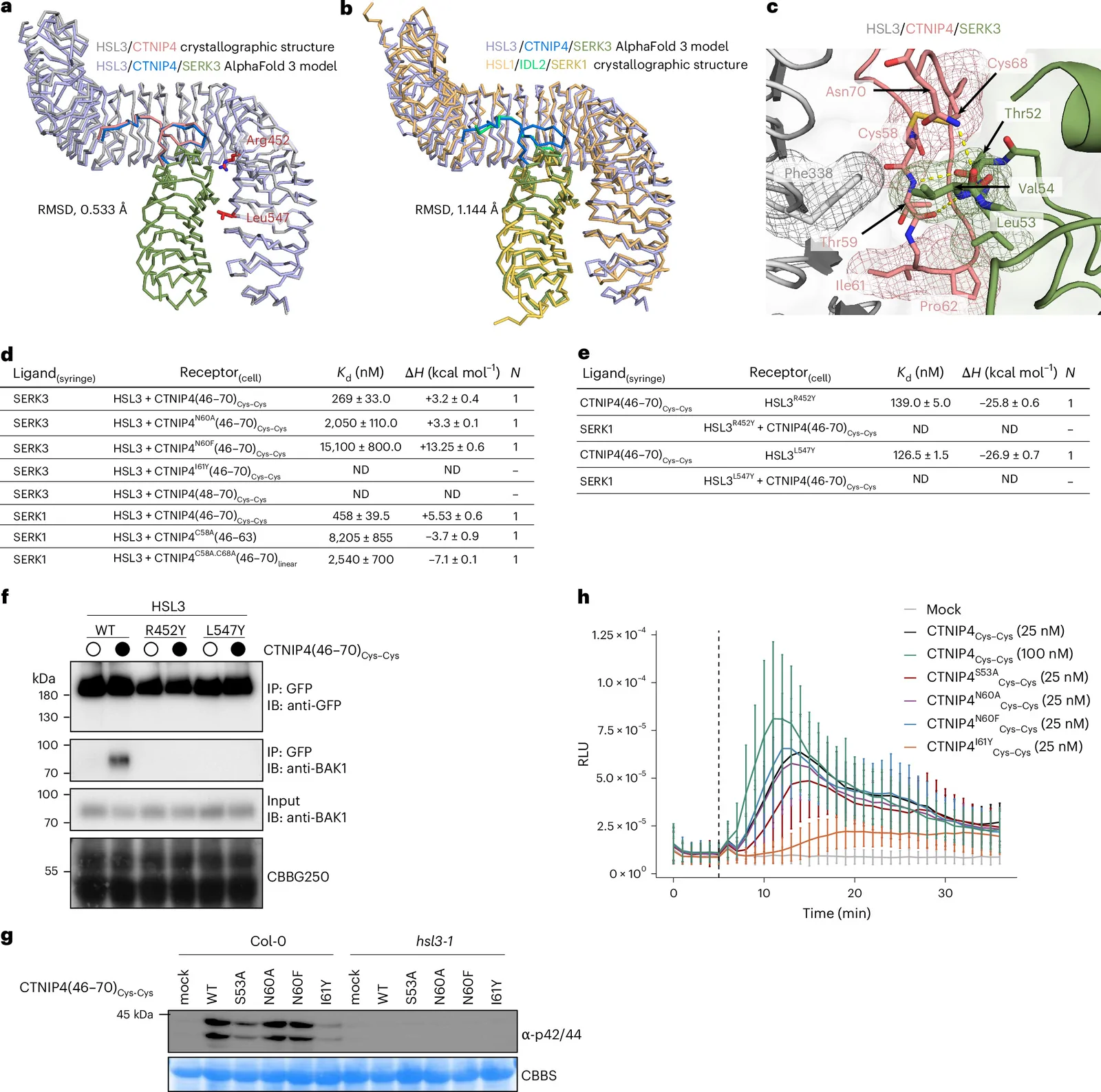
Structural basis of HSL3 phytocytokine recognition and a model for receptor activation. **a**, Structural superimposition of the HSL3–CTNIP4 complex (PDB 9T26, this study) with the AlphaFold 3 model of the ternary HSL3–CTNIP4–SERK3 complex (RMSD, 0.533 Å). Residues Arg452 and Leu547 at the receptor–co-receptor zipper-like interface are highlighted as red sticks. **b**, Structural comparison of the AlphaFold 3-predicted HSL3–CTNIP4–SERK3 complex with the experimentally determined HSL1–IDL2–SERK1 complex (PDB 7OGQ). RMSD, 1.144 Å. Structures are shown in ribbon representation. **c**, The disulfide-locked cyclic conformation of CTNIP4 bound to HSL3 generates a hydrophobic surface recognized by the N-terminal region of SERK co-receptors, with SERK3 Val54 embedded into the receptor–peptide hydrophobic pocket. Close-up view showing the C-terminal cyclic fold of CTNIP4 (pink sticks) interacting with HSL3 (grey) and the SERK co-receptor (green). Polar contacts further stabilizing the ternary complex are depicted in yellow dashes. Hydrophobic residues are highlighted by a mesh. **d**, ITC analyses of HSL3 binding to CTNIP4 variant peptides that alter the interface with the SERK co-receptors. **e**, ITC assays of HSL3 mutants targeting the receptor–co-receptor zipper-like interface using the folded CTNIP4(46–70) peptide. ITC tables summarize dissociation constants (*K*_d_, nM), stoichiometry (*N* = 1) and mean ± s.d. from at least two independent experiments; ND, no binding detected. **f**, HSL3–BAK1/SERK3 complex formation in planta. HSL3 wild-type or zipper-like surface mutants were transiently expressed in *N. benthamiana*. Protein complexes were immunoprecipitated using GFP–Trap resin and analysed by immunoblotting with anti-GFP and anti-BAK1 antibodies. Coomassie Brilliant Blue staining of the membrane is shown as a loading control. Experiments were repeated three times with similar results. IB, immunoblotting; IP, immunoprecipitation; WT, wild type. **g**, MAP kinase phosphorylation in response to 1 nM folded CTNIP4(46–70)_Cys–Cys_ interface variant peptides. Treatment was given for 20 min. Data shown are from one representative experiment out of three with similar results. **h**, Calcium influx responses in RLUs triggered by CTNIP4(46–70)_Cys–Cys_ interface mutants in wild-type Col-0^AEQ^ seedlings. The peptide concentration used was 25 nM except if indicated differently. Data represent mean ± s.d. (*n* = 60), and the vertical dashed line indicates the timing of the treatment. Source data

**Extended Data Fig. 2:**
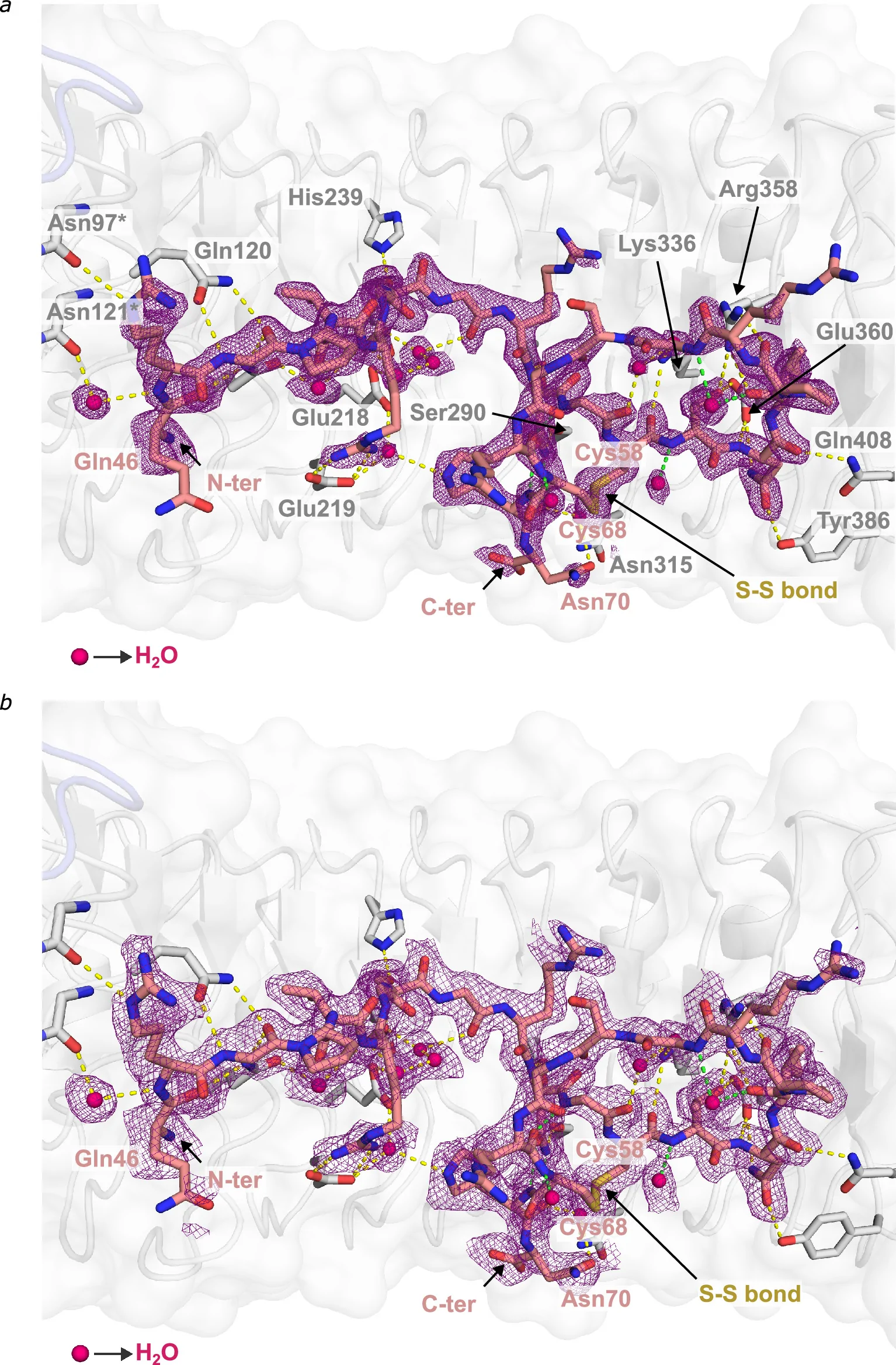
CTNIP4 omit map within HSL3 binding pocket. **a,b**, Electron-density omit map (|Fo|–|Fc|), contoured at 3σ (top) and 1.5σ (bottom) (purple mesh) highlighting the CTNIP4 peptide (pink sticks) and crystallographic water molecules (hot-pink spheres) that contribute to the polar interaction network (yellow dashed lines). Intramolecular peptide interactions are shown as green dashed lines. The HSL3 receptor is displayed in grey with interacting residues indicated. The disulfide bond between Cys58 and Cys68 constrains CTNIP4 into a cyclic conformation that is accommodated within the C-terminal ligand-binding pocket of HSL3.

**Extended Data Fig. 3:**
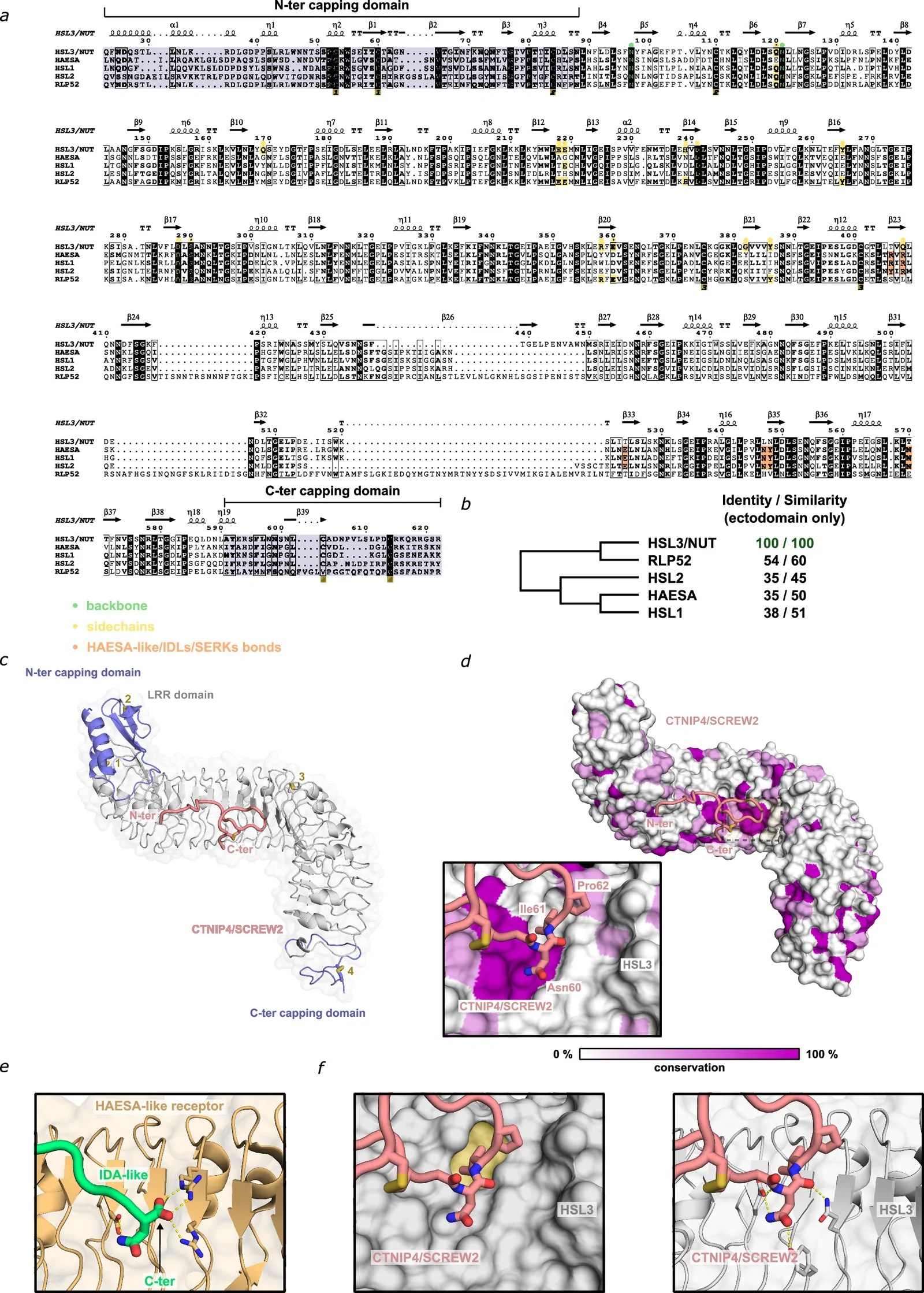
HSL3 selectively recognizes cyclic, disulfide-stabilized CTNIP peptides through a distinct C-terminal binding pocket. **a**, Comparison of HSL3 with related LRR-RKs reveals diversification of the C-terminal binding pocket, which underlies the receptor’s specificity for the cyclic, disulfide-stabilised CTNIP peptide fold. Ectodomain sequence alignment of HSL3 with its closest LRR relatives is shown, including secondary structure elements and residue numbering based on the HSL3 crystal structure (PDB ID: 9T26, this study). Residues involved in backbone and side-chain interactions with CTNIP4 are highlighted. Links to HAESA-like receptors, IDL peptides, and SERK co-receptors are indicated to illustrate structural differences that define *bona fide* ligand recognition. **b**, Phylogenetic analysis of HSL3 and closely related LRR proteins, with sequence identity scores relative to HSL3 shown. **c**, Topology of the HSL3-CTNIP4 complex, showing that HSL3 contains the canonical N-terminal and C-terminal capping domains sandwiching a solenoid composed of 21 LRRs. Conserved disulfide bonds are indicated in yellow. The CTNIP4 peptide binds to the inner surface of the LRR receptor. **d**, Surface conservation analysis of HSL3 and related LRRs from panel b, illustrating the differences in pocket architecture. **e**, Close-up view of the C-terminal ligand pocket in HAESA-like receptors coordinating the linear IDA peptide via a conserved pair of Arg residues that constrict the binding groove. **f**, Detailed view of the HSL3 C-terminal pocket in cartoon and surface representation, showing how it accommodates the cyclic loop of CTNIP4. The extended C-terminal end of HSL3 forms a unique hydrophobic cavity (highlighted in yellow) that, together with polar contacts (indicated with dashed lines), anchors the disulfide-stabilised peptide fold and enables selective peptide recognition.

**Extended Data Fig. 4:**
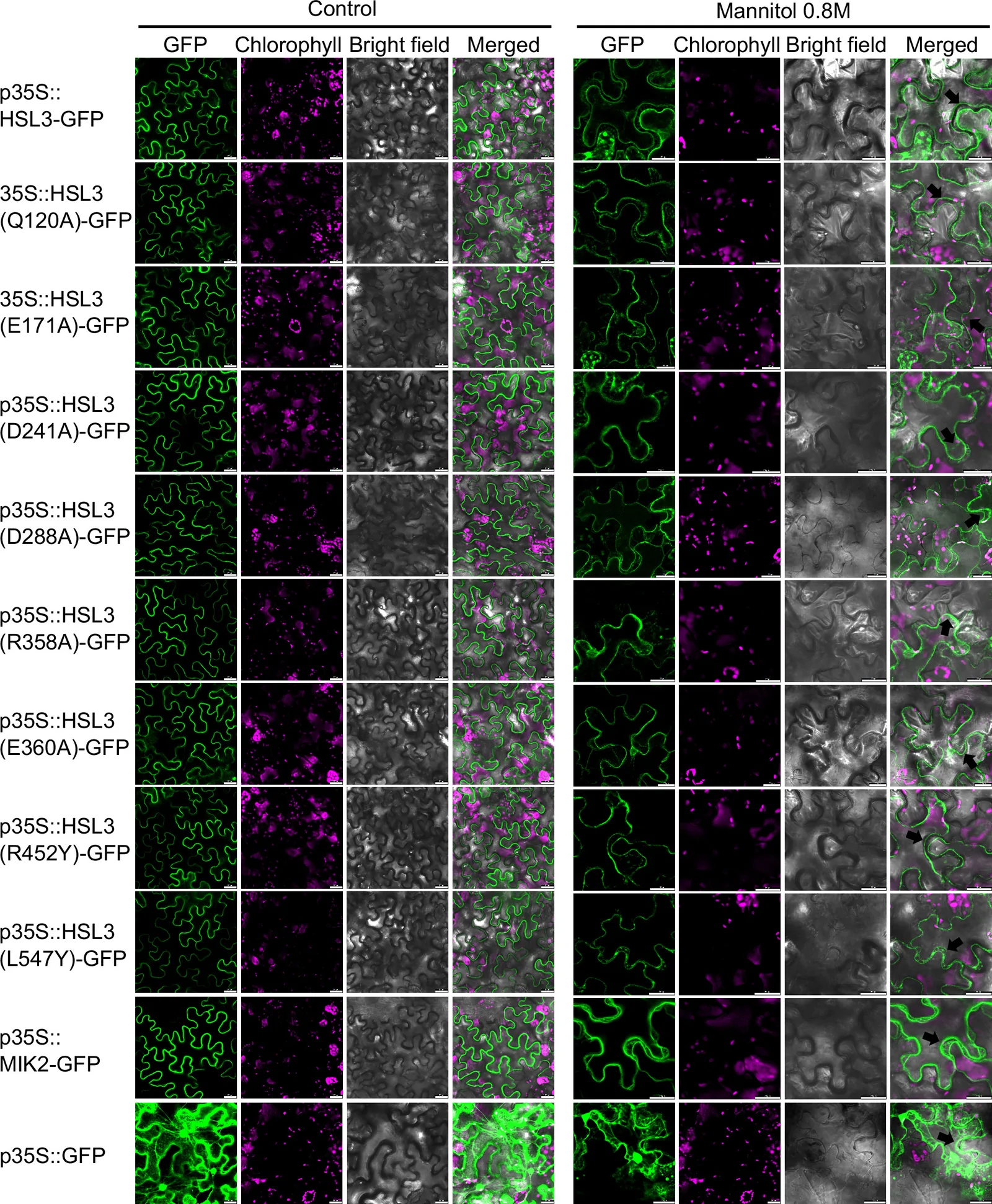
In planta plasma membrane localization of HSL3 wild-type and mutant variants. Subcellular localization of *p35S::HSL3-GFP* wild-type and mutants was analyzed via transient expression and plasmolysis assays in *N. benthamiana* leaves. The *p35S::MIK2-GFP* plasma membrane receptor and cytoplasmic *p35S::GFP* were used as controls during plasmolysis. Scale bar = 20 μm. Plasmolysis was induced by treatment with 0.8 M mannitol for 10–30 min. Arrows indicate localization of receptor proteins at the plasma membrane after plasmolysis. Source data

**Extended Data Fig. 5:**
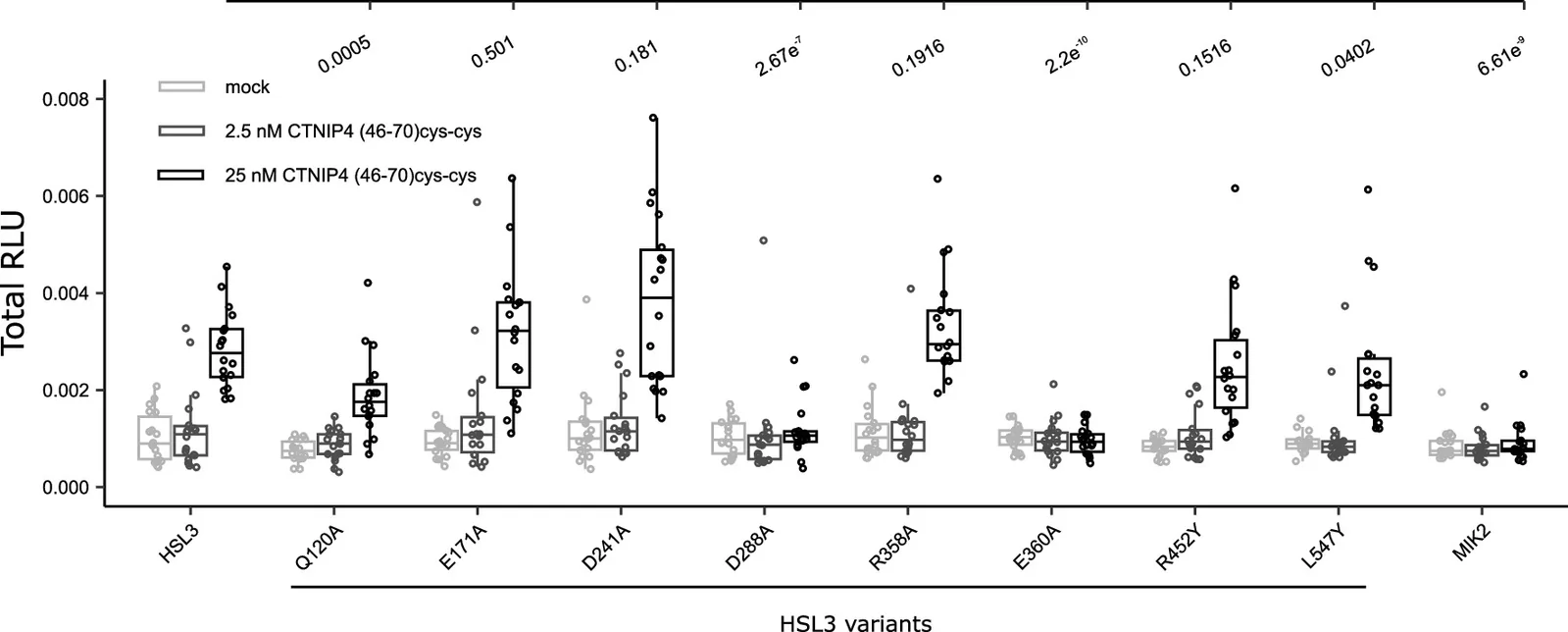
HSL3 pocket and zipper-like surface mutations impair CTNIP4-induced Ca^2+^ signaling. Box plot representation of Ca^2+^ influx assays in *N. benthamiana* plants transiently expressing HSL3 or the indicated pocket and zipper-surface mutants following treatment with mock or increasing concentrations of the CTNIP4(46–70)_Cys-Cys_ peptide. Data represent cumulative (3–30 min) relative luminescence units (RLUs). Nine independent biological replicates (n = 9 plants) were performed, each biological replicate is represented by two technical replicates. Box plots indicate the median and the interquartile range (IQR), the whiskers extend to the most extreme data points within 1.5 times the IQR. Significance was tested by performing non-parametric two-sided Wilcoxon-Mann-Whitney tests between ligand-treated HSL3 receptor vs HSL3 variants, and MIK2. Source data

**Extended Data Fig. 6:**
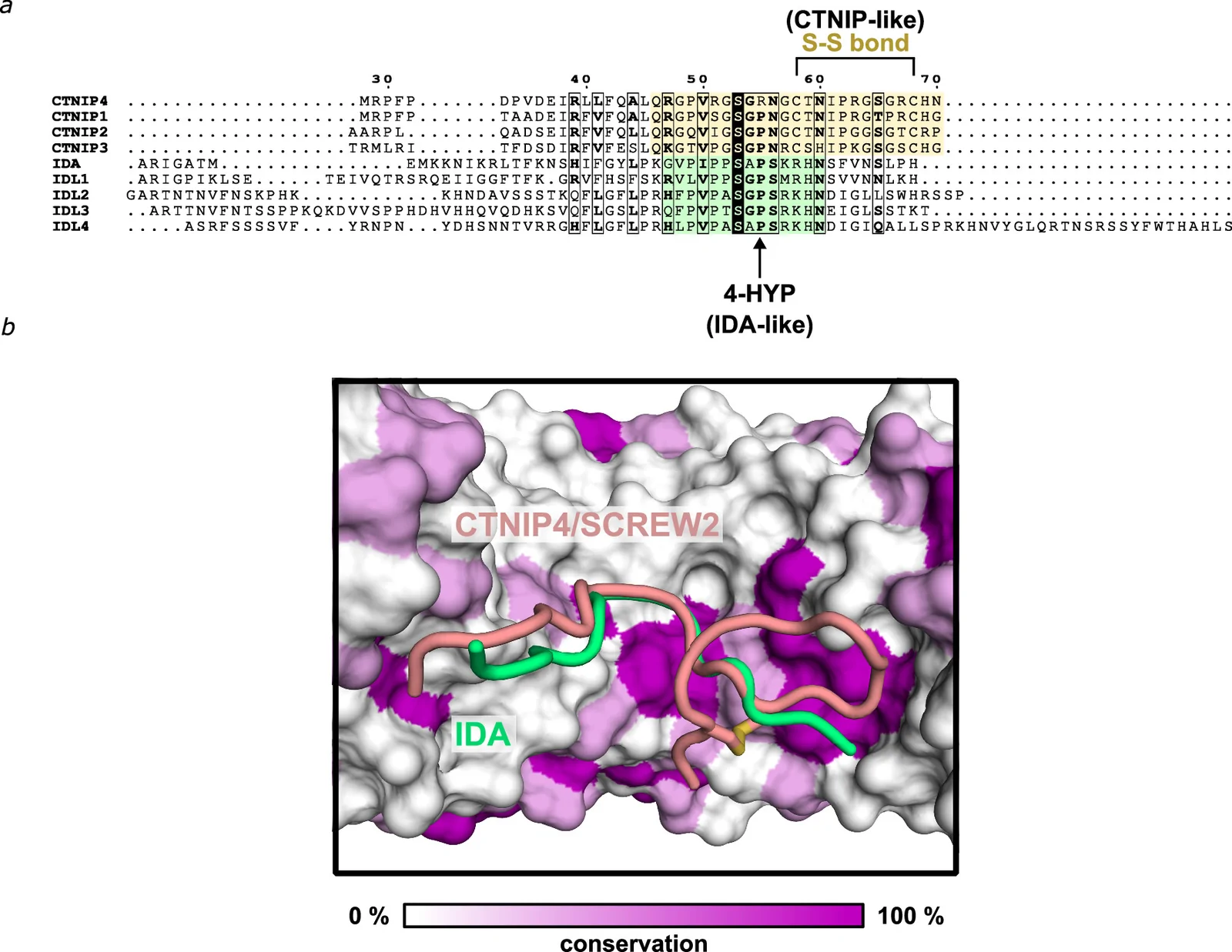
Distinct peptides have evolved a different receptor recognition mechanism. **a**, Sequence alignment of CTNIPs and IDA/IDLs. In green are depicted the mature processed IDA/IDL peptides. The hydroxylated Pro residue is indicated. In yellow are highlighted the minimal higher affinity CTNIPs mapped in this study. Disulfide bond stabilising the C-ter part of the peptide is indicated. **b**, Detail view of the pocket surface conservation of HSL3 and related LRR-RKs (Supplementary Fig. 5). The structure of HSL3-CTNIP4 is superimposed to HSL1-IDL2 (PDB: 7OGQ) to illustrate that the C-ter extension stabilised by the disulfide-bond of CTNIPs, provides a distinct receptor recognition mechanism.

**Extended Data Fig. 7:**
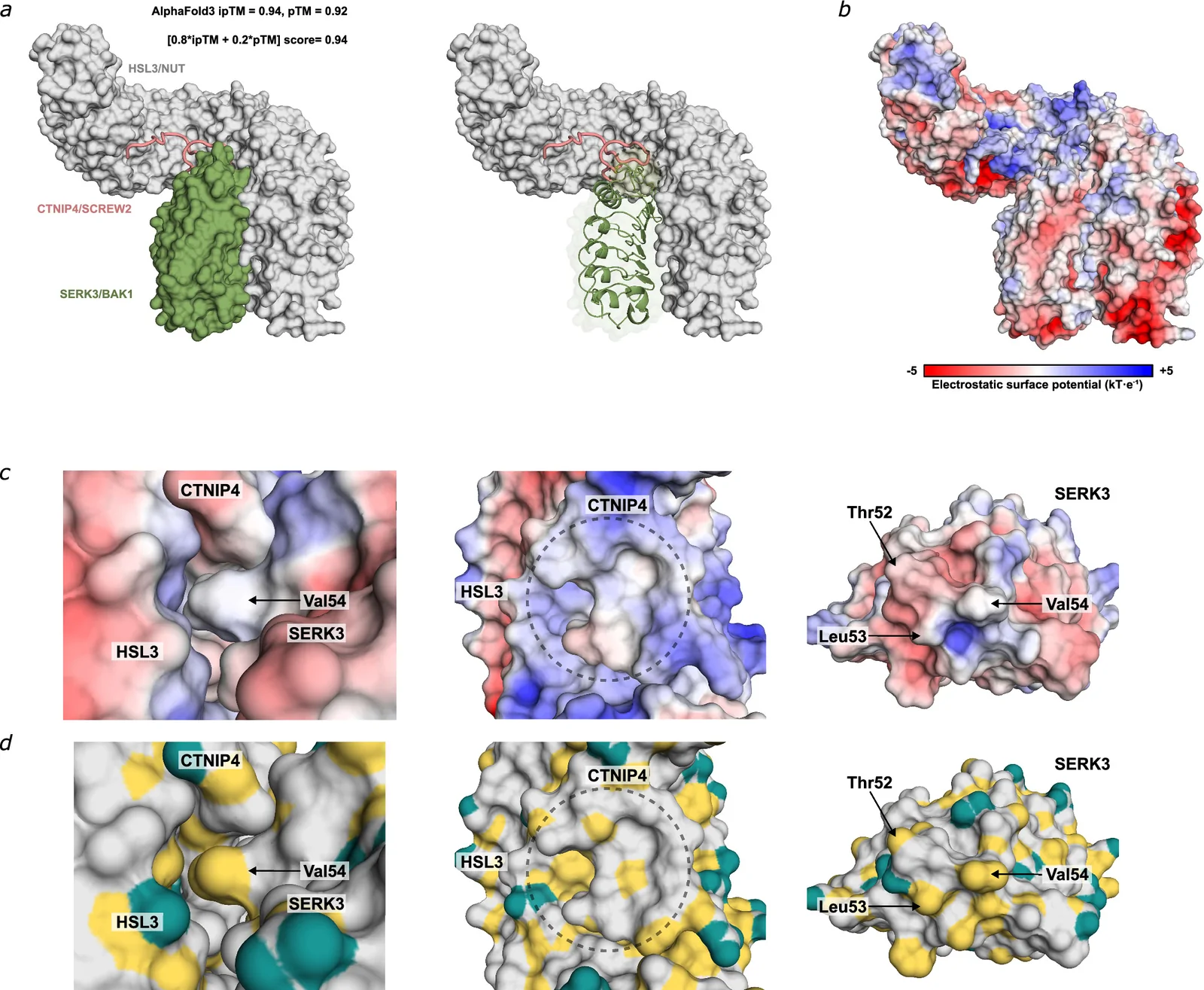
CTNIP4-induced hydrophobic interface promotes SERK co-receptor recruitment. **a**, Overall architecture of the predicted HSL3-CTNIP4-SERK3 ternary complex based on the AlphaFold model, shown in surface and cartoon representation. ipTM and pTM values are indicated. The predicted aligned error (PAE) between chains is very low (0.76–1.24 Å), supporting high confidence in the predicted protein-protein interfaces. **b**, Surface electrostatic potential of the ternary complex calculated at pH 5.0 using the APBS server^35^. Electrostatic potentials are indicated by the color scale. **c**, Close-up view of the interaction interface between the C-terminal HSL3-CTNIP4 receptor complex and the N-terminal SERK3/BAK1 co-receptor. The middle and right panels show the complementary surfaces of HSL3–CTNIP4 and SERK3 facing each other. White and light-colored regions indicate areas with low electrostatic charge as shown in panel **b**. **d**, The same interface as in **c**, colored according to residue chemical properties to highlight hydrophobic and polar contributions to the interaction. Yellow depicts hydrophobic side chains, green denotes polar groups, and grey indicates the protein backbone^36^. The circled region highlights the hydrophobic patch generated upon CTNIP4 binding to HSL3, which forms the platform proposed to recruit the SERK co-receptor.

**Extended Data Fig. 8:**
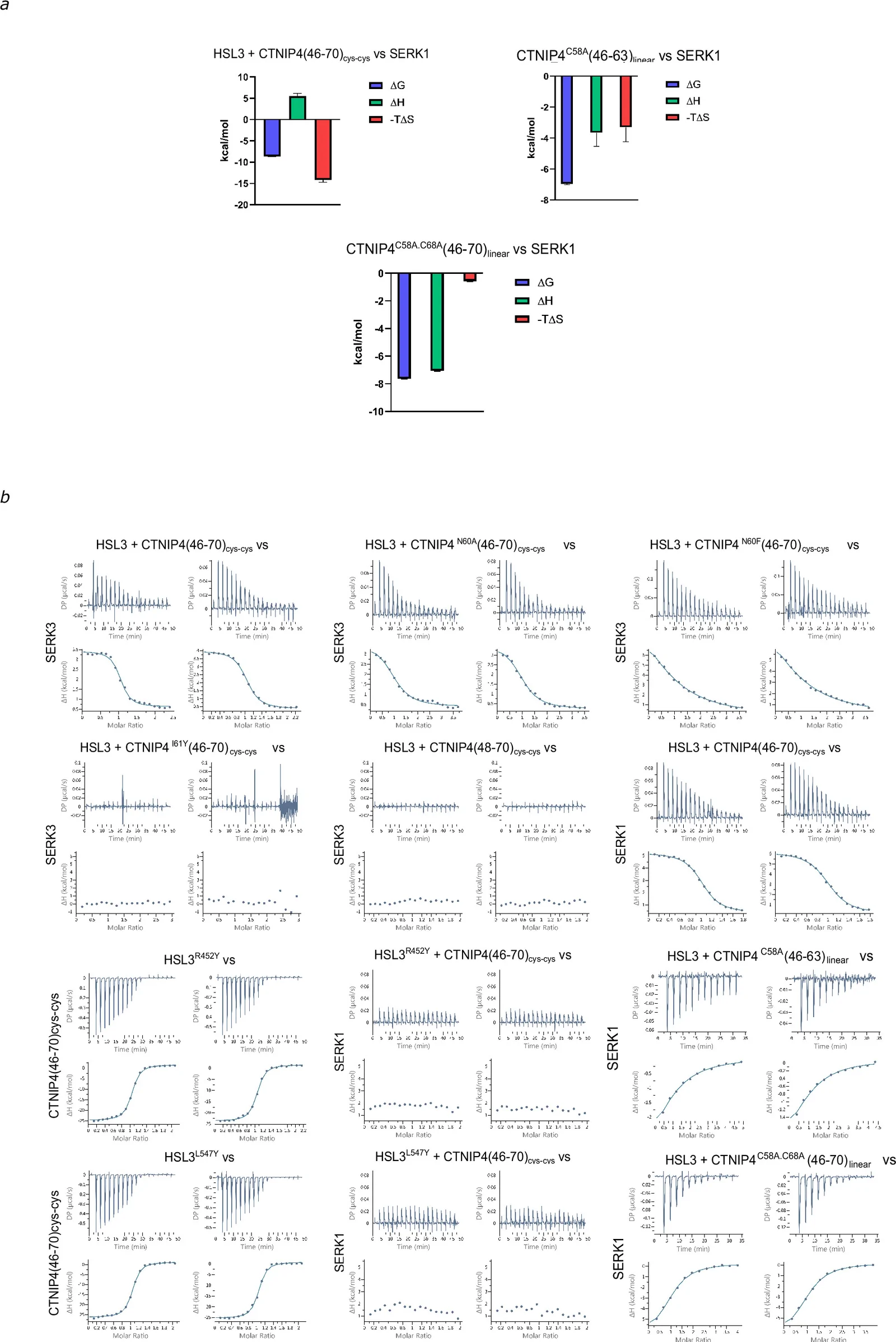
Distinct thermodynamic signatures of folded and linear CTNIP4 peptides in SERK recruitment. **a**, Thermodynamic parameters derived from isothermal titration calorimetry (ITC) measurements of SERK binding to the HSL3-CTNIP4 complex, showing the Gibbs free energy change (ΔG), enthalpy change (ΔH), and the entropic contribution (-TΔS). Binding to the disulfide-stabilized cyclic CTNIP4 peptide is predominantly entropy-driven, consistent with the formation of a hydrophobic interaction surface at the receptor-peptide interface that promotes co-receptor recruitment. In contrast, when the linear CTNIP4^C58A.C68A^ peptide is used, the interaction becomes enthalpy-driven, revealing a distinct thermodynamic signature. This shift indicates that the cyclic peptide fold generates a hydrophobic platform required for efficient SERK engagement (Fig. 2d, Fig. 3c and Extended Data Fig. 7). The enthalpy cost could be explained by the potential displacement of water molecules due to the predominantly hydrophobic nature of the interaction between the receptor-peptide interface and the co-receptor. **b**, ITC thermograms of independent replicate experiments corresponding to those shown in Fig. 3d,e.

**Extended Data Fig. 9:**
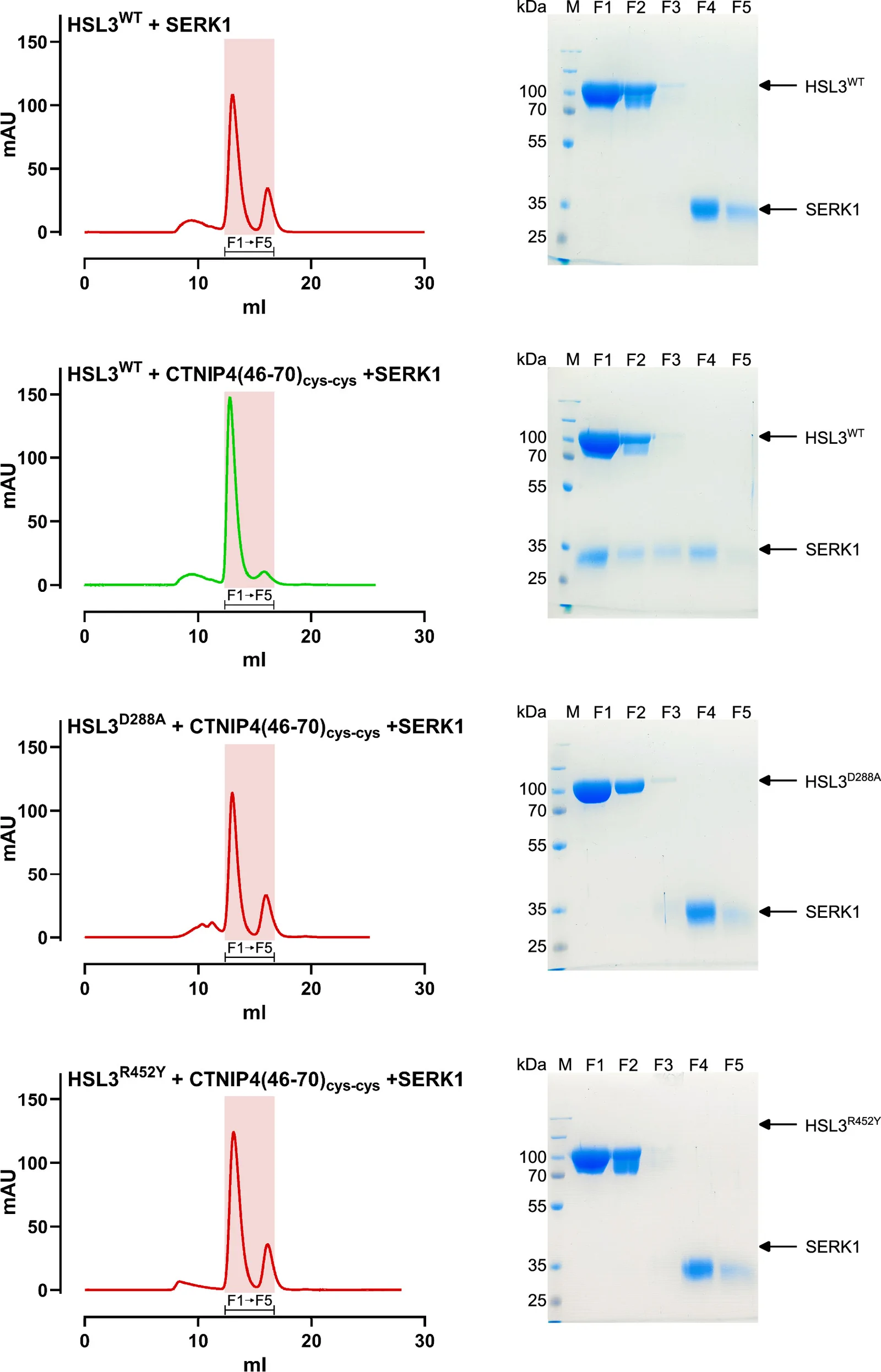
Analysis of HSL3-CTNIP4-SERK1 complex formation in solution. Size-exclusion chromatography (SEC) profiles of wild-type HSL3 and the indicated mutants in the presence or absence of CTNIP4(46–70)_Cys-Cys_ peptide. Corresponding SDS-PAGE analysis of SEC fractions 1–5 is shown for each experiment.

To elucidate the molecular basis of CTNIP4 recognition, we determined the crystal structure of the HSL3 ectodomain in complex with the bioactive CTNIP4(46–70)_Cys–Cys_ peptide at 2.12 Å resolution (Supplementary Table 1; Methods). The complex crystallized in a 1:1 stoichiometry arrangement (Fig. 2a and Extended Data Fig. 2a,b), with the receptor extracellular domain adopting the canonical architecture of that of an LRR-RK. The HSL3 ectodomain comprises 21 LRRs flanked by N- and C-terminal capping domains that frame a slightly curved solenoid scaffold^24^ (Extended Data Fig. 3a,c). The structure reveals that CTNIP4 adopts a unique disulfide-stabilized cyclic fold that is deeply embedded within the C-terminal portion of the HSL3 ligand-binding groove (Fig. 2a and Extended Data Fig. 2a,b). This compact, looped conformation is sculpted by a peptide intramolecular hydrogen-bond network and the Cys58–Cys68 disulfide bond, which locks the peptide into a three-dimensional architecture optimized for receptor recognition (Extended Data Fig. 2a,b). B-factor analysis indicates a gradient of coordination along the peptide: the linear N-terminal and core region of the peptide, extending up to Arg63, are highly ordered and tightly coordinated within the receptor pocket, whereas the cyclic loop region shows higher flexibility until the segment encompassing the disulfide bond and His69, where the covalent linkage restricts peptide flexibility and stabilizes the fold (Fig. 2b). The peptide’s preorganized conformation facilitates its precise accommodation within the receptor cavity. Consistent with this structural model, the truncated linear variant CTNIP4^C58A^(46–63), which includes only the highly coordinated region of the peptide but lacks both the C-terminal loop and the disulfide bond constraint, exhibited a ~200-fold reduction in binding affinity to HSL3 (Fig. 2c and Supplementary Fig. 2a). These data reinforce the functional importance of the C-terminal cyclic loop, whose conformational rigidity and defined topology are critical for high-affinity and specific receptor recognition. Detailed inspection of the receptor–peptide interface highlights the molecular basis for this high-affinity coordination. The N-terminal segment, containing Gln46 and Arg47 previously identified as key for binding in biochemical assays (Fig. 1b and Supplementary Fig. 2a), occupies the entrance of the HSL3 binding pocket and anchors the peptide through a network of direct and water-mediated hydrogen bonds with the receptor (Fig. 2d). Mutation of the receptor residue Gln120, which coordinates this N-terminal peptide segment, to alanine reduced binding affinity by ~60-fold, highlighting its functional relevance in peptide coordination. In the core region, additional polar interactions including those with Ser53, a residue conserved in HAE–IDL complexes, Asn56 and Arg51 further stabilize the receptor–peptide interface (Fig. 2d–f and Supplementary Figs. 1 and 3a,b). At the C-terminal end, the disulfide-stabilized cyclic loop inserts into a distinct cavity within the HSL3 ligand-binding pocket not observed in other peptide-binding LRR-RKs, where an extended network of hydrogen bonds and hydrophobic contacts reinforces peptide binding and confers overall structural stability (Fig. 2d). Within this region, CTNIP4 Ile61 plays a critical role by occupying a defined hydrophobic pocket in the receptor formed by Gly382 and surrounding residues. Substitution of Ile61 to tyrosine severely compromises this interaction and reduces binding affinity by ~120-fold (Fig. 2d,e and Supplementary Fig. 3a). By contrast, mutations of Asn60, which forms an extended hydrogen-bond network with HSL3 residues Tyr386, Gln408 and Glu360, have no dramatic effect on peptide binding to the receptor. Additional receptor residues in the core and C-terminal pocket, notably Asp288 and Glu360, contribute to the stabilization of the cyclic loop, further locking the peptide into place within the binding groove (Fig. 2d,f and Supplementary Fig. 3a,b). Consistent with these ITC measurements, expression of the corresponding HSL3 pocket mutants in vivo resulted in concordant reductions in CTNIP4-triggered Ca^2+^ influx, coupling structural anchoring to receptor output (Fig. 2d,f and Extended Data Figs. 4 and 5). Notably, the overall topology of the HSL3 ligand-binding pocket diverges from that of the HAE–HSL receptors and other closely related LRR membrane proteins, revealing a previously uncharacterized peptide-binding mode among plant receptor kinases (Extended Data Fig. 3a–f). In contrast to HAE–HSL receptors, whose pocket tapers towards the C-terminal region to accommodate the linear IDA/IDL peptides^6,7^, HSL3 displays an expanded C-terminal pocket that forms a distinct hydrophobic cavity shaped to accommodate the disulfide-locked cyclic architecture of CTNIP4. This structural extension generates a unique recognition surface that enables HSL3 to stabilize and coordinate the cyclic CTNIP4 fold, a feature absent from its sequence-related paralogues (Extended Data Fig. 3a–f). From the ligand perspective, CTNIPs and IDA/IDL peptides share a broadly similar N-terminal and core region but diverge markedly at their C-terminal end. The additional residues and disulfide-stabilized loop in CTNIPs introduce a structural determinant that promotes selective docking into the HSL3 pocket, thereby preventing cross-recognition by other IDA-type receptors and vice versa^4^ (Extended Data Fig. 6a,b). Together, these structural insights define HSL3 as a specialized receptor for cyclic CTNIP peptides, illustrating how subtle architectural variations within conserved LRR-RK frameworks can drive ligand specificity and define a new mechanism for peptide perception in plants. To investigate how CTNIP4 binding promotes receptor activation, we compared the crystal structure of the HSL3–CTNIP4 complex with AlphaFold 3-predicted models of the ternary HSL3–CTNIP4–SERK3 assembly. The model displays high confidence, with very low interchain predicted aligned error (PAE) values ranging from 0.76 to 1.24 Å, supporting a reliable prediction of the receptor–co-receptor interfaces (Fig. 3a and Extended Data Fig. 7a). Structural alignment further reveals that CTNIP4 adopts a nearly identical conformation in the binary and predicted ternary complexes (root mean square deviation, RMSD, 0.53 Å), suggesting that peptide binding to HSL3 probably pre-organizes the receptor surface for co-receptor recruitment (Fig. 3a and Extended Data Fig. 7a). In the AlphaFold-predicted ternary complex, the C-terminal cyclic loop of CTNIP4 is positioned at the interface between HSL3 and the SERK co-receptor, where it contacts the N-capping domain of SERK and appears to act as a molecular bridge between the receptor and co-receptor proteins. The model further suggests that the ternary assembly is stabilized by a zipper-like interaction surface formed between HSL3 LRRs 15–21 and SERK LRRs 1–5, extending towards the SERK C-terminal cap domain and generating a continuous receptor–co-receptor interface (Fig. 3a and Extended Data Fig. 7a). Structural comparison of the AlphaFold 3-predicted HSL3–CTNIP4–SERK3 ternary complex with the experimentally determined HSL1–IDL2–SERK1 structure suggested a similar co-receptor positioning (RMSD, 1.14 Å) and an overall conserved complex architecture, while predicting a distinct peptide-mediated activation mechanism for HSL3 (Fig. 3b). On CTNIP4 binding, the receptor–peptide interface reorganizes to generate a new predominantly hydrophobic surface along the C-terminal region of the peptide guided by the disulfide bond. In the AlphaFold-predicted ternary complex, this newly formed apolar patch seems to be directly engaging the N-terminal cap of the SERK co-receptor, acting as a molecular bridge that stabilizes the ternary assembly (Fig. 3c and Extended Data Fig. 7b–d). Supporting that hypothesis, ITC confirmed that while HSL3–CTNIP4 binding is enthalpy driven (Supplementary Fig. 2b), subsequent co-receptor recruitment is predominantly entropy driven, consistent with the burial of this hydrophobic interface (Fig. 3c,d and Extended Data Figs. 7c,d and 8a,b). Mutations disrupting the disulfide-stabilized cyclic fold or the local peptide region predicted to interact with SERKs (CTNIP4^I61Y^ and CTNIP4^N60A/F^) markedly reduced co-receptor recruitment and overall binding affinity compared with the wild-type CTNIP4(46–70)_Cys–Cys_ peptide (Fig. 3d and Extended Data Fig. 8b). Similarly, truncated or linear variants lacking the cyclic loop (CTNIP4^C58A^(46–63) and CTNIP4^C58A.C68A^(46–70)) exhibited 20–60-fold weaker binding and a pronounced shift towards enthalphy-driven interactions, indicating loss of the entropy-favoured interaction observed with the folded peptide. Together, these results highlight the importance of the disulfide-stabilized cyclic architecture, which generates the hydrophobic environment required for efficient SERK co-receptor recognition and receptor activation (Figs. 2d and 3c,d and Extended Data Figs. 7c,d and 8b). Consistent with the predicted ternary complex model, substitution of key HSL3 residues involved in peptide coordination or forming the receptor–co-receptor interface (Arg452Tyr and Leu547Tyr) abolished SERK1 association both in vitro and in vivo, supporting the proposed architecture of the ternary complex (Fig. 3a,e,f, Extended Data Figs. 7a, 8b and 9 and Supplementary Figs. 1 and 4b,c). Importantly, both HSL3^R452Y^ and HSL3^L547Y^mutants retained wild-type peptide-binding affinity (Fig. 3e, Extended Data Fig. 8b and Supplementary Fig. 1), indicating that these residues specifically contribute to the receptor–co-receptor interaction surface rather than ligand recognition. These results validate the functional relevance of the zipper-like interface predicted by the model and highlight its role in stabilizing the active signalling complex.

To translate these structural and thermodynamic findings into a physiological context, we assessed signalling competence of the receptor and peptide variants in planta using cytosolic Ca^2+^ influx and MAP kinase phosphorylation assays. CTNIP4 mutants that impaired either peptide–receptor or receptor–co-receptor interfaces exhibited a reduced Ca^2+^ influx or MAPK phosphorylation (Fig. 3g,h, Extended Data Fig. 5 and Supplementary Fig. 4a), consistent with their reduced binding affinities measured in vitro (Figs. 2e–f and 3d,e, Extended Data Fig. 8b and Supplementary Fig. 3a,b). Together, these findings provide functional support for the proposed ternary complex model and are consistent with a mechanism in which the disulfide-stabilized cyclic fold of CTNIP4 promotes SERK co-receptor recruitment by generating the hydrophobic interaction surface predicted by the AlphaFold 3 model, thereby facilitating assembly of the active signalling complex and downstream signalling.

Precise peptide recognition by structurally similar LRR-RKs is essential for coordinating diverse plant responses. Our study reveals that HSL3/NUT coordinates the CTNIP4(46–70)_Cys–Cys_ peptide with the highest affinity, thereby defining the minimal sequence and structural features for specific receptor binding and activation. Consistent with this architecture, HSL3/NUT selectively recognizes CTNIP/SCREW peptides through their N-terminal anchoring region and the disulfide-locked C-terminal cyclic loop stabilized by the Cys58–Cys68 bond. The HSL3 ligand-binding pocket differs markedly from those of other peptide-sensing LRR-RKs^6,7^, including the closely related HAE–HSL receptors. Whereas HAE–HSL receptors recognize the largely linear IDA/IDL peptides through a pocket that narrows towards the peptide C terminus, HSL3 has evolved an expanded hydrophobic cavity that specifically accommodates the disulfide-constrained cyclic architecture of CTNIP peptides. This structural innovation creates a distinct recognition surface that enables selective CTNIP perception while preventing cross-recognition by IDA/IDL receptors. More broadly, our findings illustrate how relatively subtle architectural modifications within a conserved LRR-RK scaffold can generate fundamentally different modes of peptide recognition, providing an evolutionary mechanism to expand peptide signalling repertoires while preserving specificity. A high-confidence AlphaFold 3 prediction of the HSL3–CTNIP4-SERK ternary complex further suggests that peptide binding reorganizes the receptor surface to generate a hydrophobic platform that promotes SERK recruitment, further stabilized by a zipper-like receptor–co-receptor interface. Although additional polar or solvent-mediated contacts cannot be excluded, thermodynamic analyses indicate that SERK binding to the HSL3–CTNIP4 complex is predominantly entropy driven, consistent with hydrophobic interactions dominating the interface. Accordingly, disruption of the cyclic fold or the hydrophobic interface abolishes ligand-induced interactions and signalling and shifts the interaction towards an enthalpy-driven signature, highlighting the role of the disulfide-stabilized conformation in generating the hydrophobic platform required for co-receptor engagement. Together, these findings show how the cyclic CTNIP4 fold couples ligand recognition to formation of a hydrophobic receptor surface that recruits the SERK co-receptor, providing a mechanistic framework for HSL3 activation and illustrating how conserved LRR scaffolds evolve distinct peptide recognition strategies to confer ligand specificity and orchestrate receptor–co-receptor assembly in plants.

## Methods

### Synthetic peptides

All peptides used in this study were synthesized and purchased from GenScript. Peptide sequences, including point mutations and the single disulfide bridge, are listed below. The linear production of CTNIP4(48–70)_linear_ was done according to the manufacturer and diluted right before the ITC assays to avoid oxidation.

### Peptide sequences used in this study

**Table:** 

Peptide	Sequence
CTNIP4(41–70)_Cys–Cys_	LFQALQRGPVRGSGRNG**C**TNIPRGSGR**C**HN
CTNIP4(44–70)_Cys–Cys_	ALQRGPVRGSGRNG**C**TNIPRGSGR**C**HN
CTNIP4(45–70)_Cys–Cys_	LQRGPVRGSGRNG**C**TNIPRGSGR**C**HN
CTNIP4(46–70)_Cys–Cys_	QRGPVRGSGRNG**C**TNIPRGSGR**C**HN
CTNIP4(47–70)_Cys–Cys_	RGPVRGSGRNG**C**TNIPRGSGR**C**HN
CTNIP4(48–70)_Cys–Cys_	GPVRGSGRNG**C**TNIPRGSGR**C**HN
CTNIP4(48–70)_linear_	GPVRGSGRNGCTNIPRGSGRCHN
CTNIP4^C58A.C68A^(46–70)_linear_	QRGPVRGSGRNGATNIPRGSGRAHN
CTNIP4^S53A^(46–70)_Cys–Cys_	QRGPVRGAGRNG**C**TNIPRGSGR**C**HN
CTNIP4^N60A^(46–70)_Cys–Cys_	QRGPVRGSGRNG**C**TAIPRGSGR**C**HN
CTNIP4^N60F^(46–70)_Cys–Cys_	QRGPVRGSGRNG**C**TFIPRGSGR**C**HN
CTNIP4^I61Y^(46–70)_Cys–Cys_	QRGPVRGSGRNG**C**TNYPRGSGR**C**HN
CTNIP4^C58A^(46–63)	QRGPVRGSGRNGATNIPR

Cysteine residues forming the disulfide bridge are highlighted in bold, and mutated residues are underlined.

### Protein expression and purification

The wild-type ectodomain of HSL3 (AT5G25930, residues 23–627) and mutants generated through site directed mutagenesis and confirmed by sequencing using the primers presented in Supplementary Table 2 were cloned into a modified pFastBac donor vector (Geneva Biotech) harbouring the 30K *Bombyx mori* secretion signal peptide^21^ and with a tobacco etch virus protease (TEV) cleavable C-terminal StrepII-9xHis tag. SERK1 (AT1G71830, residues 24–213) and BAK1 (AT4G33430, residues 1–220) were cloned in the same vector harbouring azurocidin and native signal peptide^25^, respectively. Baculovirus vectors were generated in DH10 MultiBac *Escherichia* *coli* cells (Geneva Biotech). Virus amplification was carried out in *Spodoptera frugiperda* Sf9 cells (Geneart, Thermo Fisher Scientific) and was used to infect *Trichoplusia ni* Tnao38 cells^26^ for protein expression. The cells were grown for 1 day at 28 °C and for 2 days at 21 °C with gentle shaking. The secreted proteins were subjected to tandem affinity purification, using Ni^2+^ (HisTrap excel, equilibrated in 25 mM KPi, and 500 mM NaCl, pH 7.8; Cytiva) and Strep columns (Strep-Tactin Superflow high-capacity; IBA) equilibrated in 25 mM Tris, 250 mM NaCl and 1 mM EDTA with pH 8.0. Affinity tags were removed using His-tagged TEV protease in a 1:50 ratio at 4 °C overnight. Separation of cleavage tags and aggregated proteins was performed using size-exclusion chromatography (SEC) on a Superdex 200 Increase 10/300 GL column (Cytiva) equilibrated in 20 mM sodium citrate and 150 mM NaCl with pH 5.0. Proteins were analysed for purity and structural integrity using SDS–polyacrylamide gel electrophoresis (PAGE).

### Crystallization and data collection

Purified HSL3 ectodomain was partially deglycosylated before crystallization. HSL3 was incubated with a mixture of Endo H and Endo F1 glycosidases for 3 h at room temperature at a ratio of 20:1:1 (protein:Endo H:Endo F1) in a buffer containing 100 mM Tris–HCl and 150 mM NaCl with pH 7.5. The partially deglycosylated HSL3 was further purified by SEC on a Superdex 200 Increase 10/300 GL column (Cytiva) equilibrated in 20 mM sodium citrate and 150 mM NaCl with pH 5.0 and used for crystallization screens.

Crystals of HSL3 in complex with CTNIP4(46–70)_Cys–Cys_ were obtained using the sitting-drop vapour diffusion technique at 18 °C. Crystals were grown in a solution containing 0.1 M citric acid with pH 4.0, 0.2 M ammonium acetate 25% (w/v) PEG 3,350, using a 1:1 ratio of protein complex to crystallization buffer (200 nl of each). Diffracting crystals were cryoprotected with mother liquor supplemented with 20% ethylene glycol and snap-frozen in liquid nitrogen.

A native, high-resolution X-ray diffraction dataset was collected at 100 K (−173 °C) at the European Synchrotron Radiation Facility (ESRF), Grenoble, France.

### Structure determination and refinement

The structure of HSL3 in complex with the CTNIP4(46–70)_Cys–Cys_ peptide was solved by molecular replacement using the AlphaFold^27^ model of HSL3 (AlphaFold ID: AF-Q9XGZ2-F1) as a search model, considering only the ectodomain. Solvent analysis suggested the presence of one HSL3/CTNIP4 complex per asymmetric unit. Molecular replacement was performed with Phaser within the CCP4 software suite^28^ and manually rebuilt in Coot^29^. The model was refined to 2.12 Å resolution using Refmac5^30^, including automatic Translation, Libration, Screw (TLS) refinement, which was applied to achieve optimal R factors. Water molecules were added where supported by electron density maps and appropriate hydrogen-bonding distances. Model validation was carried out using Coot validation tools and MolProbity^31^, and the refined coordinates have been deposited in the Protein Data Bank (PDB) under PDB 9T26.

### AlphaFold modelling of the HSL3–CTNIP4–SERK3 ternary complex architecture

The HSL3–CTNIP4–SERK3 ternary complex was modelled using AlphaFold 3 (ref. ^27^) with the server implementation and default parameters. The prediction included the extracellular domains of HSL3 (residues 23–615) and BAK1/SERK3 (residues 26–200) together with the peptide CTNIP4 (residues 46–70). Predictions were generated using seed 1829186204. Model confidence was evaluated using interface predicted template modelling score (ipTM), predicted template modelling score (pTM) and the predicted aligned error score (PAE) between protein chains.

### ITC

Experiments were performed at 25 °C using a MicroCal PEAQ-ITC (Malvern Instruments) with a 200-µl standard cell and a 40-µl titration syringe. HSL3 and its variants, together with SERKs (SERK1 and SERK3), were gel filtrated into the ITC buffer (20 mM sodium citrate, 150 mM NaCl, pH 5.0). Molar protein concentrations were calculated using individual molecular weights and molar extinction coefficients. For reference, HSL3: 68.43 kDa, 56,880 M^−1^ cm^−1^; SERK1: 21.94 kDa, 17,210 M^−1^ cm^−1^; SERK3/BAK1: 21.97 kDa, 17,085 M^−1^ cm^−1^. Values for HSL3 variants were calculated using ProtParam.

A typical experiment consisted of injecting 2 µl of a 140–800-µM solution of CTNIP4 peptides (peptides used are listed above) into a 15-µM solution of HSL3 (or variants) in the cell at 150-s intervals with a 500-rpm stirring speed. For SERKs versus HSL3/CTNIP4 peptide experiments, individual SERKs (SERK1 or SERK3) were titrated from the syringe into the cell containing 15 µM HSL3 (or variants) pre-incubated with saturating or near-saturating concentrations of CTNIP4 peptides, using the same injection pattern. ITC data were baseline corrected using the software’s offset correction. Experiments were performed in duplicates, and data were analysed with the same software. All ITC runs used for data analysis had *N* values ranging from 0.9 to 1.1, which were fitted to 1 during analysis.

### SEC analysis of HSL3–SERK complex formation

To evaluate complex formation in solution, purified ectodomains of HSL3 (wild-type or mutant variants) were mixed with SERK1 in the presence or absence of the CTNIP4(46–70)_Cys–Cys_ peptide. Proteins were exchanged into SEC buffer containing 20 mM sodium citrate and 150 mM NaCl with pH 5.0 before analysis. For complex assembly, 20 µM HSL3 was incubated with 22 µM SERK1 either alone or together with 33 µM CTNIP4(46–70)_Cys–Cys_ peptide. Mutant HSL3 variants (HSL3^D288A^ and HSL3^R452Y^) were incubated with 22 µM SERK1 and 33 µM CTNIP4(46–70)_Cys–Cys_ peptide under identical conditions. Protein mixtures (500 µl total volume) were incubated for 5 min at room temperature before injection. Samples were loaded onto a Superdex 200 Increase 10/300 GL column (Cytiva) pre-equilibrated in SEC buffer and separated by SEC. Elution profiles were monitored, and peak fractions were analysed by SDS–PAGE to assess complex formation and oligomeric state. A ternary HSL3–CTNIP4–SERK1 complex was detected exclusively for wild-type HSL3 in the presence of CTNIP4(46–70)_Cys–Cys_ and SERK1. No higher-order complex formation was observed for HSL3 or SERK1 alone nor for the HSL3^D288A^ or HSL3^R452Y^ variants in the presence of CTNIP4(46–70)_Cys–Cys_ and SERK1.

### Plant materials and conditions

*Arabidopsis thaliana* ecotype Columbia (Col-0) aequorin (Col-0^AEQ^)^32^ was used for cytoplasmic calcium measurements and were grown in growth chambers (20 °C, 60% relative humidity and 10:14 light:dark cycles). *Nicotiana benthamiana* aequorin^33^ was used for experiments which leveraged heterologous expression and were grown in the greenhouse (25 °C/22 °C day/night, 60% relative humidity and 16:8 light:dark cycles).

For MAPK assay, 10-day-old *A. thaliana* Col-0 and *hsl3-1* seedlings were used. Seeds were surface sterilized using chlorine gas for 5–6 h and plated on half-strength Murashige and Skoog (1/2 MS) media supplemented with 1% sucrose and 0.9% agar and were stratified for 2 days in the dark at 4 °C. Seeds were then moved to a growth chamber with conditions 16 h day/8 h night at 22 °C/18 °C, respectively. Germinated seedlings were transferred to 24-well plates with two seedlings per well containing 1 ml liquid 1/2 MS and grown for 7 days in the growth chamber.

### Cytoplastic calcium measurements in *Arabidopsis* and *N. benthamiana*

Leaf punches of an *Arabidopsis* line expressing aequorin were taken with a 4-mm biopsy punch of *Arabidopsis* aequorin and floated in 100 μl H_2_O with 20 μM coelenterazine (Merck), using individual cells of a white 96-well bottom plate (Greiner F-Boden, LUMITRAC, MED. Binding, (REF 655075)). After overnight incubation, the coelenterazine solution was replaced with 100 μl H_2_O and rested for approximately 30 min in the dark. Luminescence was quantified in a Berthold Tristar 3 plate reader every minute for 5 min before and 30 min after elicitor treatment using an integration time of 250 ms. R and the R packages dplyr (v1.1.2), ggpubr (v.0.6.0) and ggplot2 (v3.4.2) were used to analyse and plot the data. The resulting figure was edited in Corel-DRAW Home & Student x7. Considering experiments in *N. benthamiana*, leaf punches were taken following *Agrobacterium* infiltration for receptor expression as described below (24 h) in a stable aequorin expressing line of *N. benthamiana*. Subsequently, the above-described protocol was followed.

### Transient expression in *N. benthamiana* and confocal imaging

*N. benthamiana* does not respond to *Arabidopsis* CTNIPs, allowing the use of heterologous expression in *N. benthamiana* to test for HSL3 signaling function. *Agrobacterium tumefaciens* strain GV3101 transformed with the appropriate construct was grown overnight in Luria–Bertani media and spun down. The bacteria were resuspended in infiltration media (10 mM MES-KOH, pH 5.8, 10 mM MgCl_2_) and adjusted to an optical density at 600 nm (OD_600_) of 0.5. After 3 h of incubation, the youngest fully expanded leaves of 4–5-week-old plants were infiltrated. Protein localization was analysed 2 days after infiltration using constructs expressing HSL3 variants fused to a C-terminal GFP tag. MIK2–GFP, a known plasma membrane-localized protein, and free GFP were included as controls. Fluorescence imaging was performed using a Leica Stellaris 5 confocal microscope. GFP fluorescence was excited at 488 nm and detected between 494 and 531 nm, while chlorophyll autofluorescence was detected between 634 and 678 nm. Images were acquired using identical acquisition settings across samples and processed using LAS X software.

### MAPK phosphorylation assays

For MAPK phosphorylation assays, plant samples were ground into fine powder in liquid nitrogen homogenized in protein extraction buffer (50 mM Tris–HCl pH 7.5, 100 mM NaCl, 10% glycerol, 2 mM EDTA, 5 mM dithiothreitol, 1% IGEPAL CA630, 2 mM sodium molybdate, 1 mM sodium fluoride, 1 mM sodium orthovanadate, 4 mM sodium tartrate, protease inhibitor cocktail). Samples were incubated with rotation at 4 °C for 30 min and then centrifuged at 16,000*g* for 15 min. Supernatants were collected and SDS loading buffer was added for western blotting. Proteins were resolved using 10% SDS–PAGE and transferred to polyvinylidene difluoride (PVDF) membrane. Membranes were blocked using 5% non-fat milk and probed for MAPK activation using p44/42 MAPK (Cell Signaling, 9102, RRID: AB_330744, 1:2,000) primary antibody and horseradish peroxidase (HRP)-conjugated antirabbit IgG-peroxidase (Sigma, A0545, RRID: AB_257896, 1:10,000) secondary antibody. Protein bands were detected using enhanced chemiluminescence substrate (Thermo Scientific, 32106 or 34096). Coomassie Brilliant Blue solution was used to detect protein loading control.

### Co-immunoprecipitation assays

*A. tumefaciens* strains carrying constructs for the expression of HSL3, HSL3 receptor variants or BAK1^D416N^ were adjusted to an OD_600_ of 0.5, mixed in a 1:1 ratio and co-infiltrated into leaves of *N. benthamiana*. After 24 h, infiltrated leaves were bisected and midveins removed to allow mock and CTNIP4 treatments to be performed on paired samples derived from the same infiltration event. Leaf halves were incubated in 0.5× MS medium supplemented with sucrose for 30 min before treatment. Samples were then vacuum-infiltrated for 2 min and incubated for 10 min in either 50 nM CTNIP4 peptide diluted in 0.5× MS-sucrose or 0.5× MS-sucrose alone as a mock control. Treated leaves were blotted dry and immediately flash-frozen in liquid nitrogen. For co-immunoprecipitation assays, approximately 3.5 g frozen tissue was ground to a fine powder in nitrogen-cooled stainless-steel jars using an MM400 ball mill (Retsch). The powder was resuspended in extraction buffer (50 mM Tris–HCl pH 7.5, 150 mM NaCl, 2 mM EDTA, 10% (v/v) glycerol, 1 mM dithiothreitol (DTT) and protease inhibitor cocktail equivalent to Sigma-Aldrich P9599 at 1:100 dilution) using 2 ml buffer per gram of tissue. Protein extraction was performed for 30 min at 4 °C with gentle rotation. Extracts were filtered through two layers of Miracloth and clarified by centrifugation at 15,000*g* for 30 min at 4 °C. Protein concentrations were determined using the Bradford assay and extracts were normalized to equal protein amounts.

Protein extracts containing GFP-tagged HSL3 or the corresponding mutants were incubated with 10 µl GFP–Trap agarose beads (Chromotek) for 3 h at 4 °C with gentle rotation to immunoprecipitate receptor complexes. Beads were collected by centrifugation at 500*g* for 2 min and washed three times with 1 ml extraction buffer. After the final wash, beads were resuspended in 40 µl 2× Laemmli SDS sample buffer and heated at 80 °C for 10 min to elute bound proteins. A total of 20 µl of each immunoprecipitated fraction was separated on 8% SDS–PAGE gels for 90 min at 120 V and transferred to PVDF membranes. Membranes were probed with anti-GFP (B-2, Santa Cruz Biotechnology) and anti-BAK1 antibodies.

### Anti-BAK1 antibody generation

Anti-BAK1 antibodies were generated against a peptide targeting the C terminus of BAK1 (CDSTSQIENEYPSGPR) and coupled to keyhole limpet haemocyanin for immunization. Peptide synthesis, conjugation and immunizations were performed by Genscript. Rabbit immune serum was used for affinity purification against bead-coupled antigen peptide. The specificity of affinity-purified antibody was confirmed by immunoblotting against protein extracts from *Arabidopsis* seedlings lacking BAK1 protein (Supplementary Fig. 4c).

### Reporting summary

Further information on research design is available in the Nature Portfolio Reporting Summary linked to this article.

## Supplementary information

Supplementary Information Supplementary Figs. 1–4 and Tables 1 and 2.

Reporting Summary

Peer Review File

Supplementary Data 1 Unprocessed western blot and Coomassie-stained gel for Supplementary Figs. 2 and 4.

## Source data

Source Data Fig. 1 Statistical source data for Fig. 1c and unprocessed western blot and Coomassie-stained gel for Fig. 1d.

Source Data Fig. 3 Statistical source data and unprocessed western blot and Coomassie-stained gel for Fig. 3f,g.

Source Data Extended Data Fig. 1 Source data for Extended Data Fig. 1.

Source Data Extended Data Fig. 4 Source data for Extended Data Fig. 4.

Source Data Extended Data Fig. 5 Source data for Extended Data Fig. 5.

## Data Availability

All data supporting the findings of this study are available in this article and its Supplementary Information. The coordinates and structure factors for the HSL3–CTNIP4 complex have been deposited in the Protein Data Bank under accession code 9T26. Raw isothermal titration calorimetry (ITC) thermograms and size-exclusion chromatography (SEC) chromatograms are available via Zenodo at https://doi.org/10.5281/zenodo.21479519 (ref. ^34^). Source data are provided with this paper.
