# Supplementary material for: A disulfide bond sculpts the CTNIP4 phytocytokine fold for recognition by the receptor kinase HSL3
Source: Nat Plants. 2026 Sep 17;12(9):1688–97. doi: 10.1038/s41477-026-02380-y (PMC13585564; doi:10.1038/s41477-026-02380-y)
Supplement: Supplementary file 1 [file 41477_2026_2380_MOESM1_ESM.pdf]

nature plants

Supplementary information https://doi.org/10.1038/s41477-026-02380-y

A disulfide bond sculpts the CTNIP4 phytocytokine fold for recognition by the receptor kinase HSL3

In the format provided by the authors and unedited

Supplementary information:

Title: A disulfide bond sculpts the CTNIP4 phytocytokine fold for recognition by the receptor kinase HSL3

Authors: Pedro Jiménez-Sandoval[1], Oliver Johanndrees[2,4#], Simon Snoeck[2#], Chitthavalli Y. Harshith[2#], Moutasem Omary[2#], Caroline Broyart[1#], Jack Rhodes[3], Kyle W. Bender[2], Cyril Zipfel[2,3*], Julia Santiago[1*]

Affiliations:

[1] The Plant Signaling Mechanisms Laboratory, Department of Plant Molecular Biology, University of Lausanne, Lausanne, Switzerland.

[2] Institute of Plant and Microbial Biology, Zurich-Basel Plant Science Center, University of Zurich, Zurich, Switzerland

[3]The Sainsbury Laboratory, University of East Anglia, Norwich Research Park, United Kingdom

[4]Present address: Lonza, Visp, Switzerland.

[#]equal contributions

***Corresponding authors**: cyril.zipfel@botinst.uzh.ch, julia.santiago@unil.ch.

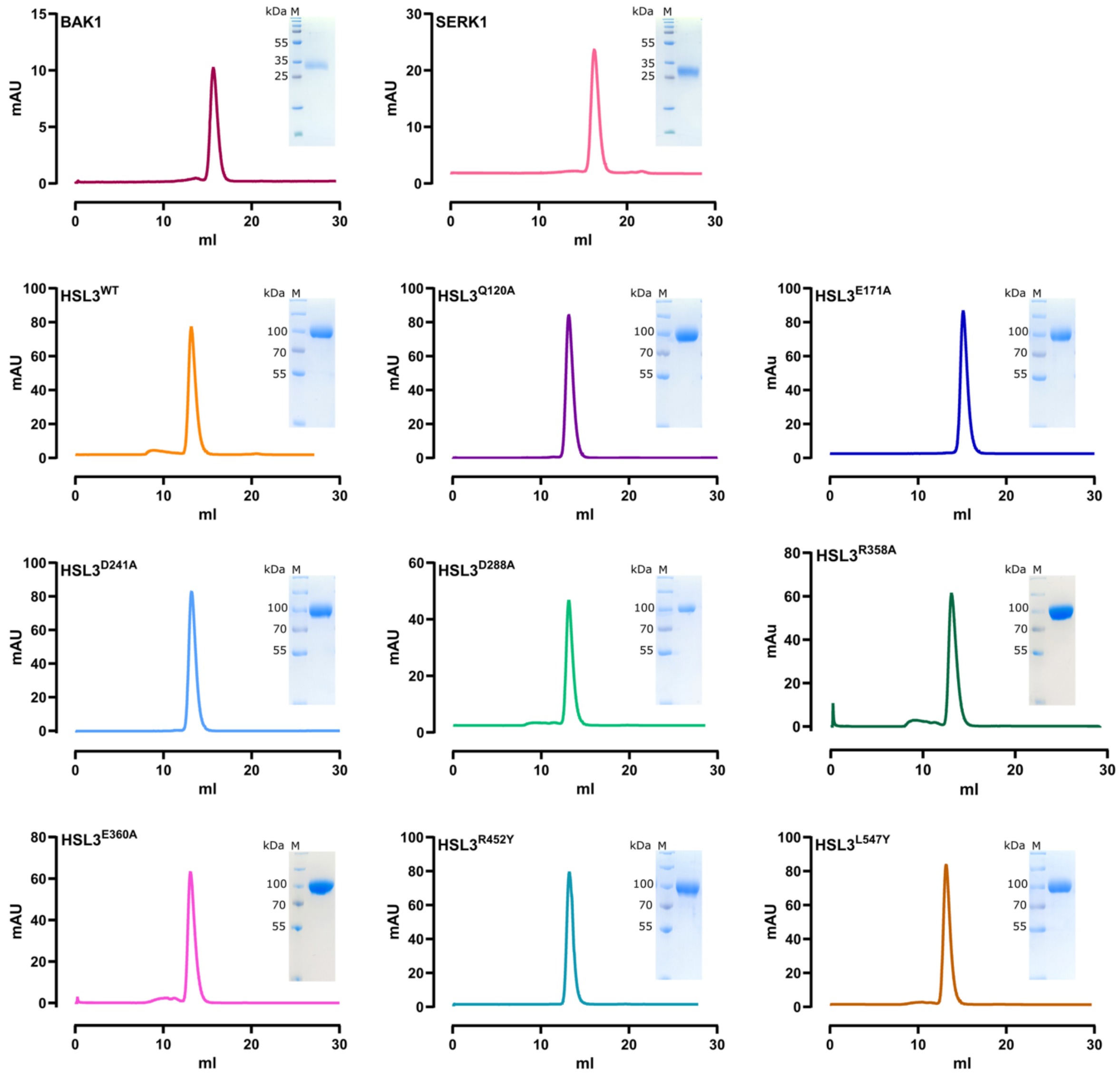

Supplementary Figure 1. Purification profiles of HSL3 wild-type, HSL3 variants, and SERKs co-receptor proteins. Size-exclusion chromatography (SEC) traces and SDS–PAGE analyses of insect cell-expressed and purified HSL3 wild-type, HSL3 variant, and SERK co-receptor ectodomains.

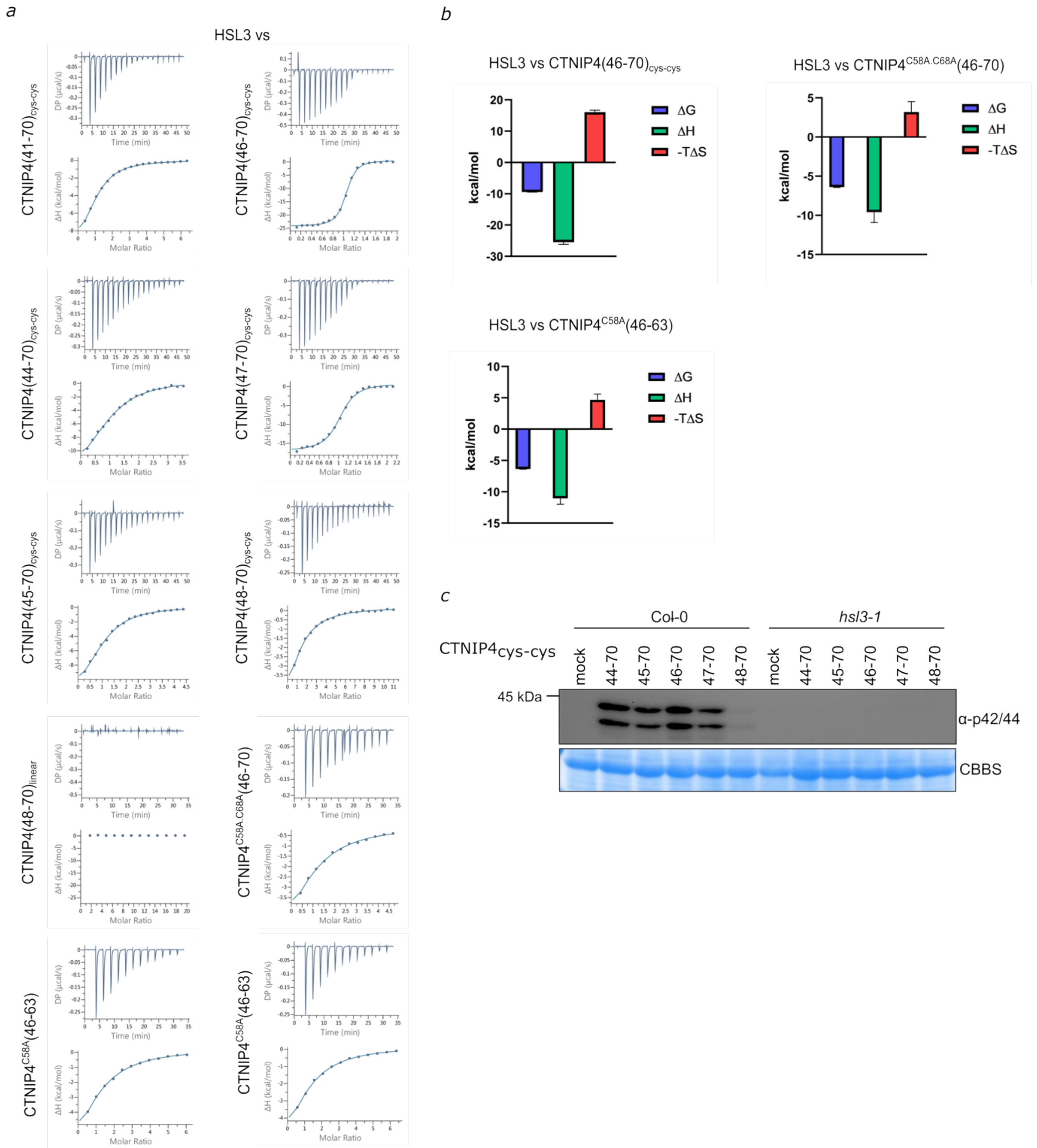

Supplementary Fig. 2. ITC experiments and thermodynamic parameters of CTNIP4 variants and MAPK activation replicate of experiments shown in Fig. 1. a, ITC thermograms of independent replicate experiments corresponding to those shown in Fig. 1b and 2c. **b**, Thermodynamic profiles of CTNIP4 peptides binding to HSL3 derived from ITC measurements, showing changes in Gibbs free energy (ΔG), enthalpy (ΔH), and the entropic contribution (−TΔS). **c**, Independent replicate of MAP kinase (MAPK) activation assays shown in Fig. 1d.

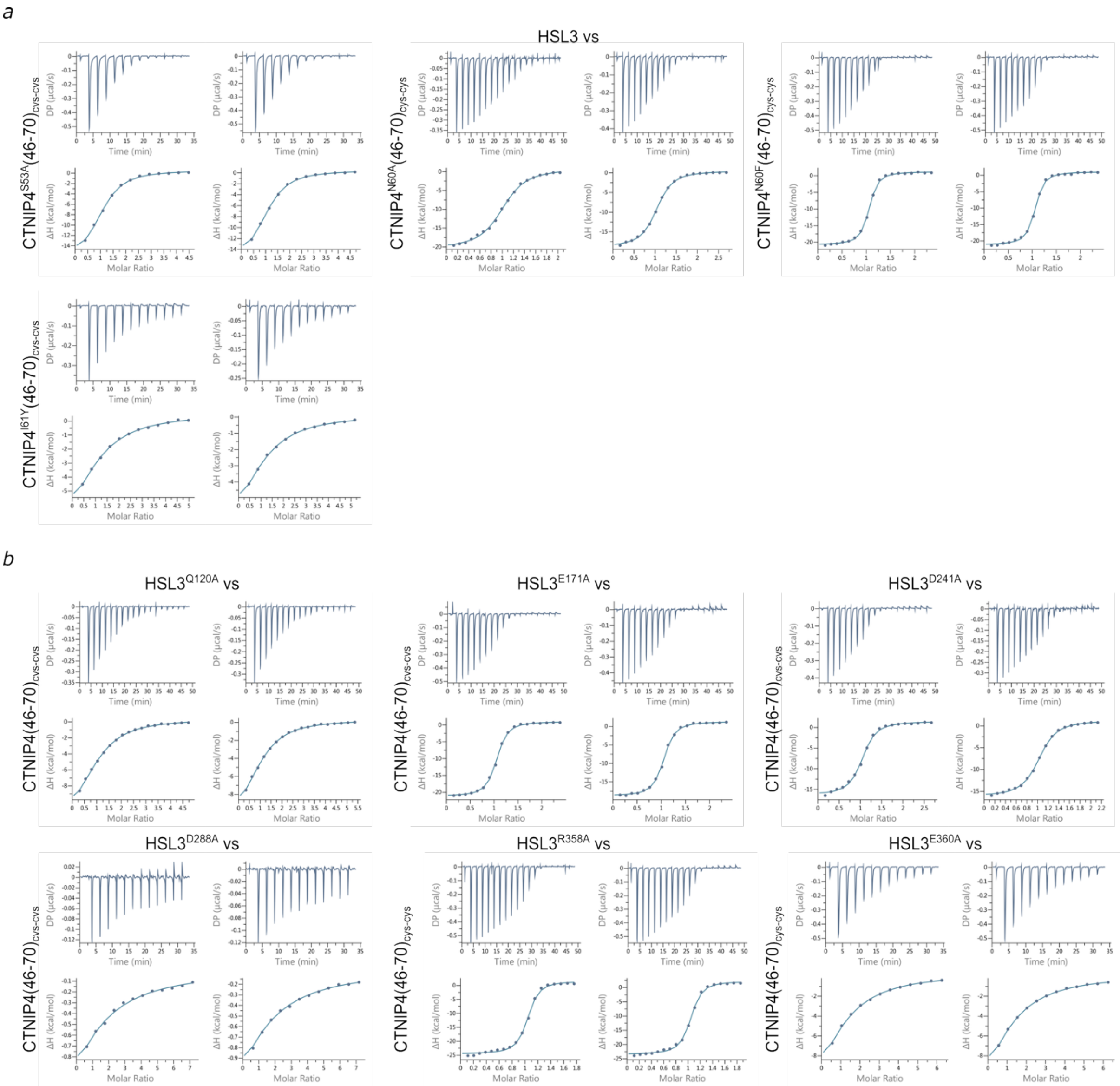

Supplementary Fig. 3. ITC experiments of HSL3 wild-type and variants vs CTNIP4 peptide. a, ITC thermograms of independent replicate experiments corresponding to those shown in Fig. 2e. **b,** ITC thermograms of independent replicate experiments corresponding to those shown in Fig. 2f.

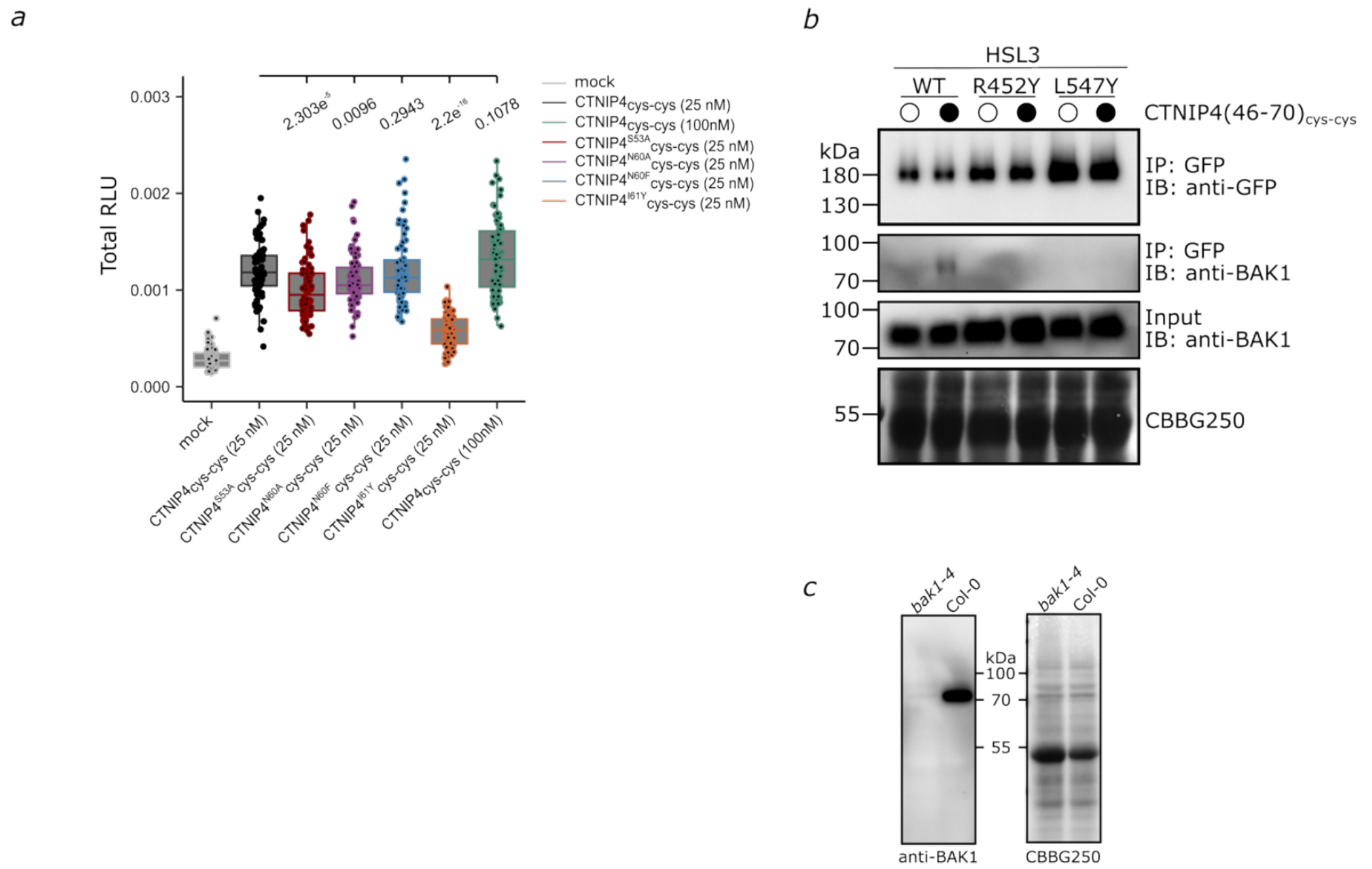

Supplementary Fig. 4. Ca^{2+} signaling analysis and CoIP validation of HSL3-CTNIP4-SERK interface mutants. a, Considering the cumulative RLUs (3-30 min) of experiments shown in Fig. 3g, the box plots indicate the median and the interquartile range (IQR), the whiskers extend to the most extreme data points within 1.5 times the IQR. Significance was tested by performing non-parametric Wilcoxon-Mann-Whitney tests between CTNIP4(46-70)$_{cys\text{-}cys}$ and CTNIP4(46-70)$_{cys\text{-}cys}$ variants. **b**, CoIP replicate of experiment shown in Fig. 3f (50 nM CTNIP4 peptide used). **c**, validation of the anti-BAK1 antibody by immunoblotting against protein extracts from Col-0 or the *bak1-4* mutants. The antibody detects a band of ~70 kDa specifically in Col-0.

Supplementary Table 1. Crystallographic table of the complex HSL3-CTNIP4.

Data collection and refinement statistics (molecular replacement)

	PDB ID: 9T26
**Data collection**	
Space group	C 2 2 21
Wavelength (Å)	0.96770
Cell dimensions	
a, *b*, *c* (Å)	56.69, 131.09, 220.99
α, β, γ (°)	90.00, 90.00, 90.00
Resolution (Å)	110.50 - 2.12 (2.18 - 2.12)*
R_{meas}	0.087 (0.878)
R_{pim}	0.041 (0.406)
CC(1/2) (%)	98.5 (77.3)
$I / \sigma I$	9.1 (1.2)
Completeness (%)	98 (97.7)
Redundancy	4.1 (4.2)
**Refinement**	
Resolution (Å)	110.50 - 2.12
No. reflections	45998
R_{work} / R_{free}	0.19 / 0.23
No. atoms	10044
Protein	9,371
Ligand/ion	447
Water	226
B-factors	58.05
Protein	56.34
Glycosylations	99.76
Water	52.32
R.m.s. deviations	
Bond lengths (Å)	0.016
Bond angles (°)	2.061
Ramachandran (%)	
favored	85.14
allowed	13.41
outliers	0.81
Molprobity results	
Molprobity score	1.83

*Single crystal diffraction. *Values in parentheses are for highest-resolution shell.

Supplementary Table 2. Primers used to generate HSL3 variants for recombinant protein expression in insect cells.

**HSL3 mutant**	**Primers**
Gln120Ala	Fw→GACTTGTCCGCGAACCTGCTCAACGGTTCC<br>Rv→GAGCAGGTTCGCGGACAAGTCCAGGTACTG
Glu171Ala	Fw→TACCAGTCCGCGTACGACGGTACTTTCCCA<br>Rv→ACCGTCGTACGCGGACTGGTACAGGTTCAG
Asp241Ala	Fw→GAACACGTGGCCCTCTCCGTGAACAACCTG<br>Rv→CACGGAGAGGGCCACGTGTTCCAGGTCGGT
Asp288Ala	Fw→GTGTTCCTCGCCTTGTCCGCTAACAACTTG<br>Rv→AGCGGACAAGGCGAGGAACACCAGGTTGGT
Arg358Ala	Fw→AAGTTGGAGGCTTTCGAGGTGTCCGAGAAC<br>Rv→CACCTCGAAAGCCTCCAACTTGGAGTGGAC
Glu360Ala	Fw→GAGCGTTTCGCGGTGTCCGAGAACCAGCTG<br>Rv→CTCGGACACCGCGAAACGCTCCAACTTGGA
Arg452Tyr	Fw→AACATGTCCTATATCGAGATCGACAACAAC<br>Rv→GATCTCGATATAGGACATGTTCCAGGCCAC
Leu547Tyr	Fw→CCTCGTCTGTATAACCTGGATCTGAGCGAA<br>Rv→ATCCAGGTTATACAGACGAGGCAGCAAGCC
