# Supplementary material for: A disulfide bond sculpts the CTNIP4 phytocytokine fold for recognition by the receptor kinase HSL3
Source: Nat Plants. 2026 Sep 17;12(9):1688–97. doi: 10.1038/s41477-026-02380-y (PMC13585564; doi:10.1038/s41477-026-02380-y)
Supplement: Supplementary file 2 [file 41477_2026_2380_MOESM2_ESM.pdf]

nature portfolio

Corresponding author(s): *Double-anonymous peer review submissions: write DAPR and your manuscript number here instead of author names.*

Last updated by author(s): *YYYY-MM-DD*

Reporting Summary

Nature Portfolio wishes to improve the reproducibility of the work that we publish. This form provides structure for consistency and transparency in reporting. For further information on Nature Portfolio policies, see our Editorial Policies and the Editorial Policy Checklist.

Statistics

For all statistical analyses, confirm that the following items are present in the figure legend, table legend, main text, or Methods section.

n/a	Confirmed	
☐	☒	The exact sample size (*n*) for each experimental group/condition, given as a discrete number and unit of measurement
☐	☒	A statement on whether measurements were taken from distinct samples or whether the same sample was measured repeatedly
☐	☒	The statistical test(s) used AND whether they are one- or two-sided<br>*Only common tests should be described solely by name; describe more complex techniques in the Methods section.*
☒	☐	A description of all covariates tested
☒	☐	A description of any assumptions or corrections, such as tests of normality and adjustment for multiple comparisons
☐	☒	A full description of the statistical parameters including central tendency (e.g. means) or other basic estimates (e.g. regression coefficient) AND variation (e.g. standard deviation) or associated estimates of uncertainty (e.g. confidence intervals)
☐	☒	For null hypothesis testing, the test statistic (e.g. *F*, *t*, *r*) with confidence intervals, effect sizes, degrees of freedom and *P* value noted<br>*Give P values as exact values whenever suitable.*
☒	☐	For Bayesian analysis, information on the choice of priors and Markov chain Monte Carlo settings
☒	☐	For hierarchical and complex designs, identification of the appropriate level for tests and full reporting of outcomes
☒	☐	Estimates of effect sizes (e.g. Cohen's *d*, Pearson's *r*), indicating how they were calculated

Our web collection on statistics for biologists contains articles on many of the points above.

Software and code

Policy information about availability of computer code

Data collection	Calcium: LightCompass®, version 6.16, Basic Edition (Berthold Technologies)<br>MAPK: ChemiDoc (Bio-rad)
Data analysis	Data analysis and plotting: R and the R-packages dplyr (v1.1.2), ggpubr (v.0.6.0), and ggplot2 (v3.4.2)<br>Figures: Corel-DRAW Home & Student x7 and Inkscape 1.3<br>MAPK: Image Lab Touch Software (v2.2.0.08)<br>Microscopy: LAS X office (v1.4.5.27713)

For manuscripts utilizing custom algorithms or software that are central to the research but not yet described in published literature, software must be made available to editors and reviewers. We strongly encourage code deposition in a community repository (e.g. GitHub). See the Nature Portfolio guidelines for submitting code & software for further information.

Data

Policy information about availability of data

All manuscripts must include a data availability statement. This statement should provide the following information, where applicable:

- Accession codes, unique identifiers, or web links for publicly available datasets
- A description of any restrictions on data availability
- For clinical datasets or third party data, please ensure that the statement adheres to our policy

The authors declare that the data (of each individual replicate) supporting the findings of this study are available within the manuscript and its supplementary files.

Research involving human participants, their data, or biological material

Policy information about studies with human participants or human data. See also policy information about sex, gender (identity/presentation), and sexual orientation and race, ethnicity and racism.

Reporting on sex and gender	N/A
Reporting on race, ethnicity, or other socially relevant groupings	N/A
Population characteristics	N/A
Recruitment	N/A
Ethics oversight	N/A

Note that full information on the approval of the study protocol must also be provided in the manuscript.

Field-specific reporting

Please select the one below that is the best fit for your research. If you are not sure, read the appropriate sections before making your selection.

☒ Life sciences ☐ Behavioural & social sciences ☐ Ecological, evolutionary & environmental sciences

For a reference copy of the document with all sections, see nature.com/documents/nr-reporting-summary-flat.pdf

Life sciences study design

All studies must disclose on these points even when the disclosure is negative.

Sample size	No sample size calculation was performed. Sample sizes reflect or exceed previous established protocols and are provided in the figure legends.
Data exclusions	N/A
Replication	Experiments were repeated using diverse plant batches on different days. Replicates are consistently mentioned in the figure legends. All findings were successfully reproduced, id est, we did not observe any inconsistencies across data replications. In the case of the in vitro binding assays, the number of replicates is indicated in the figure legend.
Randomization	All ligands (variants) compared were tested on the same leaf for each replicate. Leaf disks were randomly collected while avoiding veins.
Blinding	Blinding during data collection and/or analysis is not relevant to our study due to the quantitative (automated) nature of Calcium influxes (plate reader).

Reporting for specific materials, systems and methods

We require information from authors about some types of materials, experimental systems and methods used in many studies. Here, indicate whether each material, system or method listed is relevant to your study. If you are not sure if a list item applies to your research, read the appropriate section before selecting a response.

Materials & experimental systems

n/a	Involved in the study
☐	☒ Antibodies
☐	☒ Eukaryotic cell lines
☒	☐ Palaeontology and archaeology
☒	☐ Animals and other organisms
☒	☐ Clinical data
☒	☐ Dual use research of concern
☐	☒ Plants

Methods

n/a	Involved in the study
☒	☐ ChIP-seq
☒	☐ Flow cytometry
☒	☐ MRI-based neuroimaging

Antibodies

Antibodies used

α-p44/42 MAPK (Erk1/2) #9102 (Cell Signaling Technology, 1:2000); α-BAK1 (this study)

Validation

Validation statements for commercial antibodies are available from the corresponding manufacturers' websites.
New batch of α-BAK1 antibodies was validated using BAK1 knock-out mutant, see Sup. Fig. 13c.

Eukaryotic cell lines

Policy information about cell lines and Sex and Gender in Research

Cell line source(s)

BTI-TNAO38

Authentication

non of the cells used were authenticated in this study

Mycoplasma contamination

No mycoplasma contamination was detected

Commonly misidentified lines
(See ICLAC register)

n/a

Plants

Seed stocks

The Arabidopsis aequorin line was previously reported https://doi.org/10.1186/s12870-014-0374-4. hsl3-1 T-DNA insertion mutant was previously reported in https://doi.org/10.7554/eLife.74687

Novel plant genotypes

No novel plant genotypes were created/used for this study.

Authentication

The flg22-induced cytoplasmic calicium influx was consistently measured as described in the methods section to confirm the identity of the Arabidopsis aequorin line. For MAPK assays, hsl3-1 mutant line was used as negative control to capture CTNIP specific responses.
