# Supplementary material for: A disulfide bond sculpts the CTNIP4 phytocytokine fold for recognition by the receptor kinase HSL3
Source: Nat Plants. 2026 Sep 17;12(9):1688–97. doi: 10.1038/s41477-026-02380-y (PMC13585564; doi:10.1038/s41477-026-02380-y)
Supplement: Supplementary file 3 [file 41477_2026_2380_MOESM3_ESM.pdf]

nature portfolio

Peer Review File

A disulfide bond sculpts the CTNIP4 phytocytokine fold for recognition by the receptor kinase HSL3

Corresponding Author: Professor Julia Santiago

This file contains all reviewer reports in order by version, followed by all author rebuttals in order by version.

Version 0:

Reviewer comments:

Reviewer #1

(Comments for the Author)
This paper reports the co-crystal structure analysis of the receptor kinase HSL3 and its ligand CTNIP4. The authors first identified CTNIP4(46–70) Cys-Cys as the minimal unit that exhibits high binding affinity for HSL3. They then performed co-crystal structural analysis of the HSL3–CTNIP4 complex and elucidated the structural details of the HSL3 ligand-binding pocket. By comparing the crystal structure of the HSL3–CTNIP4 complex with an AlphaFold3-predicted model of the HSL3–CTNIP4–SERK ternary complex, they further demonstrated that recruitment of the SERK co-receptor occurs through a hydrophobic interface generated upon peptide binding. A comparison with the phylogenetically related IDA–HAE co-crystal structure also revealed that subtle structural changes within the LRR-RK framework control ligand specificity. Collectively, the authors shed light on one aspect of the diverse mechanisms of ligand recognition by plant peptide hormone receptors.

The series of results is supported by precise and solid co-crystal structural analyses, and I find no logical inconsistencies; the work is highly convincing. This work will likely be well received as a new successful example of co-crystallization studies of peptide ligand–receptor pairs. On the other hand, this reviewer is not fully convinced that the discovery of a hydrophobic pocket accommodating the disulfide-bond-stabilized cyclic CTNIP4 constitutes an impact strong enough to reshape established concepts of receptor–ligand recognition. A co-crystal structure of a disulfide-bond-stabilized cyclic peptide with an LRR protein has already been reported by the same authors for RALF4–LRX8 (Proc. Natl. Acad. Sci. USA 117, 7494–7503 (2020)). The loop region of RALF4, structurally stabilized by a disulfide bond, is essential for its high-affinity binding to LRX8. RALF4 peptide adopts a defined conformation when binding to the LRX LRR domain by making contacts with the binding pocket. Thus, it may be argued that the present study simply demonstrates that diverse receptor structures exist to accommodate diverse ligand architectures, rather than providing evidence for a fundamentally new concept in receptor–ligand recognition.

Reviewer #2

(Comments for the Author)
Summary:
The manuscript by Jiménez-Sandoval and coworkers provides a thorough structural and biochemical characterization of the HSL3–CTNIP4 receptor–ligand interaction. CTNIPs are peptides acting as phytocytokines and are bound by the HSL3/NUT LRR-RK, which induces heteromerization of the complex with SERK/BAK receptors. The authors redefine the minimal bioactive form of the CTNIP4 peptide as CTNIP4(46–70)Cys–Cys, which exhibits nanomolar affinity to its receptor and and shows robust signaling activity. The 2.12 Å crystal structure of HSL3-CTNIP4 reveals a unique recognition mechanism. CTNIP4 adopts a cyclic loop conformation that is recognized by an extended C-terminal hydrophobic pocket of HSL3, distinguishing it from other LRR-RKs that bind linear peptides. Functional assays (Ca^{2+} influx, MAPK activation) mirror ITC binding profiles and support the structural model.

Originality and Significance
The study is technically well conducted, relevant and interesting. The structure of the HSL3-CTNIP4 complex has not previously been determined and reveals a novel cyclic loop conformation, by which HSL3 binds CTNIP4 and ensures specificity for the ligand, preventing cross-activation with other LRR-RK. In addition, the study maps the optimal length for the bioactive CTNIP4.

Strengths:
- Mapping of affinity and functionality based on peptide length, revealing the peptide for optimal bioactivity
- High-resolution crystal structure of HSL3-CTNIP4 revealing a novel cyclic loop conformation that is critical for ligand binding
- Both biochemical and functional data, which strengthens the study.

Title, Abstract and introduction:
The title reflects the content of the manuscript. The abstract is well written and describes the presented study, as does the introduction. Relevant literature is cited (I focused on the structural biology refs). In general the language of the manuscript is clear, understandable and concise. I have pointed out the few typos below.

Data and Methodology:
Overall the experimental design and methodology is sound. I have a few detailed comments:

Crystallography:
I was not able to inspect the model/structure factors directly, as 9T26 is not yet released on the Protein Data Bank (I guess it's in HPUB?). Based on the reported crystal data, the structure seems to be of good quality, but I have several comments for clarification. Processing is conservative but sound. However, crystallographic data quality reporting in Table 1 relies on Rmerge/Rsym, which is outdated and redundancy dependent (Karplus & Diederichs, 2015). Redundancy independent R values are preferred, (Rpim or Rmeas) but at a minimum, the half-set correlation CC1/2 has to be reported in Table 1. It would be good to include the wavelength as well.

The Fo–Fc omit map (Supplementary Figure 3) is shown at 1.5 sigma? Is this a typo/an oversight and is it the 2Fo–Fc map instead, for which the sigma cutoff would be more appropriate? This is a rather low sigma value for the Fo-Fc difference map which is more prone to showing noise. Factors like low resolution, partial occupancies and flexibility can indeed require that a lower sigma cutoff than the usual 3 sigma is chosen and appropriate, but for a high resolution 2.12 Å crystal structure it is a bit unusual. Please clarify.
For a clearer representation of the omit map I would also consider to omit the receptor from the graphics and only show the density around the ligand itself. Another panel could then still show the receptor as well.

Also if the receptor is shown, the sidechains shown in stick representation (I guess they are important for binding?) should be labeled.

Figure 2b: What is a "low" and what a "high" B-factor? Please provide B-factor values.

Protein expression, purification, ITC assays:
Protein expression and purification is described well and protein quality is shown reliably in Supplementary Figure 1. Given the high affinity of the HSL3-CTNIP4(46-70)-SERK interaction, can this complex be isolated on SEC, as done for other LRR-RK complexes?

The ITC curves in the figures are too small. I appreciate the amount of ITC data and encourage very much to show individual replicates, but as it is now, one can hardly see them.
The general ITC methodology is strong and convincing and well described in the method section.

Structure prediction:
The methodology for the deep learning structure prediction using Alphafold3 of the HSL3-CTNIP4-SERK complex is not present in the Materials & Methods sections. It should be included, even if "only" the server implementation of Alphafold 3 was used. Modeling parameters, number of models etc... should be provided. Predictions should be supported by their relevant quality indicators such as iPTM, pTM, pLDDT, PAE. This is especially important here, as a complex architecture is predicted.

Conclusions:
The conclusions support the data well. I have a few comments to strengthen the manuscript:

1: The manuscript shows that longer N-terminal CTNIP4 variants (45–70) bind markedly weaker than 46–70 and suggests that additional N-terminal residues hinder recognition. It is intriguing that affinity decreases by more than x5000 by addition of a single residue, L45. What is the structural mechanism behind this? It is clear that residues 46-48 are perceived very stringently by the receptor and could act as an additional anchor (Figs 1 & 3) and it makes sense that removing these comes at a cost of affinity. But the mechanism behind the addition of a single residue decreasing affinity would be very relevant. It would support the author's point that 46-70 is indeed the bioactive CTNIP if a structural feature on the receptor could be identified that could read this amino acid.

2: What would really strengthen this manuscript would be experimental structural data for the ternary complex (CTNIP–HSL3–SERK). This might not be technically feasible, but it would address a central question about the binding mode: what does SERK recognize? The AlphaFold model provides a plausible hypothesis, but still leaves some questions:

- Figure 3 states "Upon CTNIP4 binding, the receptor-peptide interface reorganises to generate a new predominantly hydrophobic surface along the C-terminal region of the peptide guided by the disulfide bond. This newly formed apolar patch directly engages the N-terminal cap of the SERK co-receptor, acting as a molecular bridge that stabilises the ternary

assembly (Fig. 3c)."
While this is a compelling observation, Figure 3C illustrating this surface needs improvement. The ITC dissection of receptor-ligand binding do show convincing the entropy vs enthalpy driven binding, so the conclusions are sound, but the current figure with the mesh is more confusing than illustrating. One could e.g. add an additional panel to show the electrostatic properties of the hydrophobic interaction surface.

- Figure 2 suggests that the C-terminal part of CTNIP4 has high ADPs, is poorly ordered and not strongly coordinated by HSL3 (Figure 2d, a bit difficult to read). As shown in Fig. 3, this stretch is also hardly recognized by SERK (according to the AF model, Fig 2c. To me, there is a bit of a conceptual paradox: The presence of such a large interaction surface created by the cyclic loop and then the distal C-terminal residues being neither well ordered nor clearly utilized by receptor or co-receptor. Could the AF prediction be biased by training on existing linear peptide complexes deposited in the PDB? Deleting this stretch (63-70) has a large effect on affinity, how do individual amino acids contribute?

Could there be more to this binding mechanism or could the ternary complex could look different from the AF prediction shown in Figure 3 as it looks like that SERK3 does hardly interact with those residues but mostly with the CTNIP motif?

Minor comments and typos:

Typos:
Line 89: "Suppleme mtary" -> Supplementary
Line 99: "stoichoimetry"
Line 113 "The peptide preorganized conformation facilitates"... -> "The peptide's preorganized conformation facilitates"
Line 150: "Suplementary"
Line 499: Ca+2 influx assays -> Ca2+ influx assays

Figure 1:
Panel A is not very intuitive and could benefit from reformatting. I can see that affinity of HSL3 to CTNIP4 finds an optimum around 46/47, with both shorter and longer peptides not being perceived with high affinity. But the color-coded panel is too small for that. Could the representation be improved along with the rest of the figure and include also the functional data, as they correlate quite well with the binding data?

Supplementary Fig 5.
What is shown in Panels a, b? Legend incomplete

Supplementary Fig. 7.
Legend missing for Panel d. The description for Panel c refers to Figure 3b, which is a structure figure, not ITC data.

Reviewer #3

(Comments for the Author)
This manuscript by Sandoval et al. reports the crystal structure of the Arabidopsis LRR receptor-like kinase HSL3 in complex with the disulfide-stabilized cyclic peptide ligand CTNIP4, complemented by biochemical and physiological analyses. The study elucidates how HSL3 specifically recognizes the phytocytokine CTNIP4, offering insights relevant to stomatal immunity and receptor-like kinase (RLK) research. Although the work presents compelling structural and functional data, several major concerns should be addressed.
1. The current title resembles that of a review article and should be revised to more specifically reflect the key findings, including the protein names involved.
2. While the crystal structure of the HSL3–CTNIP4 complex and the accompanying biochemical and physiological data are robust, the analysis of the ternary HSL3-CTNIP4-SERK3 complex based solely on AlphaFold-3 predictions remains highly speculative. Drawing conclusions about conformational changes, interaction surfaces, and key residues from a predicted model is not supported.
3. Several conclusions appear overstated. For instance, claims such as “uncover a previously unrecognized mechanism,” “a previously uncharacterized cavity,” and “employs a novel mechanism to perceive CTNIP/SCREW peptides” should be moderated or more precisely contextualized.
Specific points:
• Line 27: The statement “a feature absent in the closely related HAE/HSL LRR-RKs” is misleading without further clarification regarding HAE/HSL.
• Line 30: The phrase “these structural, biochemical and physiological insights uncover a previously unrecognised mechanism of peptide perception and receptor activation” should be revised. Since most ligand–receptor interactions are unique, the authors are encouraged to specify the proteins involved, as suggested for the title.
• The structure determination method should be briefly mentioned in the main text, and the Ramachandran plot should be provided in the Table S1 to show the model quality.
• Line123 The N-terminal residues 46-47 of SCREW2, as well as residues involving binding in HSL3, should be tested through mutation, as these two residues seem very important for the high affinity binding
• Line 128: The description “a previously uncharacterized cavity” requires clarification, has an HSL3 structure been reported before?

• Lines 138–147: There appears to be a logical inconsistency in the description of the overall topology and binding pocket. SCREW2 and IDA peptides seem to bind HSL proteins in a similar region, with the N-terminal part aligned but the C-terminal part diverging (Fig. S6), could this represent a distinct peptide-binding mode (as suggested by Fig. 3b)?
• Lines 156–171: The use of an AlphaFold-3 predicted structure to analyze conformational changes and pinpoint interaction residues is speculative. While a rough model may be acceptable, detailed structural comparisons and interaction analyses (e.g., Fig. 3) should be avoided or significantly condensed. Moreover, mutational analyses focused largely on CTNIP4, with limited attention to SERK3 residues.
• Figure 1: The ITC binding curves could be moved to a supplementary figure to improve clarity.

Version 1:

Reviewer comments:

Reviewer #1

(Comments for the Author)
The LRR-RK subfamily XI functions as a class of receptors that recognize a wide variety of endogenous and exogenous peptide ligands. Because the extracellular LRR domain consists of relatively well-conserved repeating motifs, how ligand recognition specificity is achieved has long been a central question of interest to structural biologists. The elucidation of the crystal structure of the disulfide bond–containing peptide ligand CTNIP4 in complex with its receptor HSL3 provides a new example illustrating how subtle structural differences within the LRR domain can give rise to ligand selectivity.
Although some of the analyses rely on AlphaFold3-based predictions rather than experimental crystal structure data (perhaps reflecting current trends in the field), which represents a weakness of the study, the presented data as a whole are expected to contribute to a deeper understanding of peptide signaling.
The authors' response to this reviewer's comment is largely limited to an expansion of the Discussion section; however, in the revised manuscript, the conceptual advance of their study is described more concretely, which should help readers better understand the work.
However, the current version of the Discussion section leaves the reader with a rather dry impression. A more in-depth discussion—for example, regarding what biological advantages the newly identified ligand recognition mechanism may confer (i.e., why it has been maintained under diverse evolutionary selection pressures), as well as similarities and differences with ligand recognition mechanisms in receptors from other species—would further strengthen the manuscript.

Reviewer #2

(Comments for the Author)
Overall, I find that the revised manuscript has improved in response to the reviews. The authors have addressed my main technical concerns about crystallographic data reporting, electron density visualisation, B-factors, and the documentation of structural predictions. Importantly, they have added new experimental support for the proposed SERK-binding interface. Several earlier over-statements (e.g. regarding the "mature" peptide) were moderated and now the ternary complex is correctly presented as a prediction that is constrained by experiments rather than having the confidence level of an experimental structure. My remaining comments below are mostly clarifications and minor points about data presentation, not additional experiments.

1: OUR RESPONSE: We thank the reviewer for this helpful comment. The crystallographic data were originally reported following the template provided by Nature Plants. In response to the reviewer's suggestion, we have now updated Supplementary Table 1 to include the additional recommended data-quality metrics.

- The crystallographic data reporting is according to the state-of-the-art now, thanks.

2. OUR RESPONSE: We thank the reviewer for this constructive suggestion. The omit map shown corresponds to an |Fo–Fc| difference map contoured at 1.5σ, which we used to better visualize certain side-chain features of the peptide, particularly the arginine residues that participate in the interaction network. While we agree that higher contour levels are typically preferred for difference maps, the lower contour level was chosen here to allow clearer visualization of these features. To address the reviewer's concern and improve clarity, we have now added an additional panel showing the omit map contoured at 3σ, which more strictly highlights the well-defined electron density corresponding to the peptide. In addition, we have revised the figure to improve interpretability: the relevant receptor residues involved in peptide binding are now labeled, and the map representation has been adjusted to more clearly focus on the density surrounding the ligand. These changes are now included in the relabelled Supplementary Fig. 4.

- I thank the authors for including the 3σ Fo–Fc omit map and for improving the labelling of the ligand and receptor residues. At 2.1 Å resolution, the peptide backbone and most side chains are already well supported at 3σ (consistent with Fig. 2 B factors).

If I understood it correctly, the long arginine side chains and the high-B regions participate in the predicted SERK interface. I would therefore in fact expect that they appear weaker or partially disordered in the binary HSL3–CTNIP4 complex. Without the co-receptor, these side chains are partially exposed, have elevated B-factors and correspondingly look patchy in the omit map. This is exactly what the 3σ map shows and it is fully consistent with the proposed mechanism. In contrast, the

1.5σ map contains noise and, while it is visually more “complete”, is not necessary to support the structural conclusions. It may encourage over-interpretation of poorly ordered side chains. I would therefore consider the 3σ panel completely sufficient; I am satisfied with the structural evidence without the lower contouring map.

3. OUR RESPONSE: We thank the reviewer for this helpful comment. To clarify the interpretation of the B-factor distribution, we have now included the corresponding B-factor values directly in Fig. 2b.

- Thanks, the labeling of the scale is sufficient and evident for at even the high-B region are still quite well defined (73.85 as max B).

4. OUR RESPONSE: We thank the reviewer for this constructive suggestion. Following this recommendation, we performed size-exclusion chromatography (SEC) experiments to assess the ternary complex formation between HSL3, CTNIP4 and the SERK co-receptor. We have now included these data as new Supplementary Fig. 12, which complements the biochemical and new in vivo analyses presented in Fig. 3.
The SEC profiles show that the HSL3-SERK ternary complex is formed in a peptide-dependent manner, consistent with the ITC measurements. Furthermore, analysis of receptor mutants demonstrates that disruption of CTNIP4 binding prevents ternary complex formation, whereas mutations affecting the zipper-like receptor-co-receptor interface abolish SERK association even in the presence of the peptide. These results further support our structural model and biochemical data and highlight the requirement for both peptide binding and the zipper interface to stabilize the active ternary complex. The manuscript has been updated accordingly, and the new data are described in the Results section that now reads: page 6-7 lines 196-207.“Consistent with the predicted ternary complex model, substitution of key HSL3 residues involved in peptide coordination or forming the receptor-co-receptor interface (Arg452Tyr and Leu547Tyr) abolished SERK1 association both in vitro and in vivo, supporting the proposed architecture of the ternary complex (Fig. 3a,e,f and Supplementary Figs. 1, 10 ,12 and 13). Importantly, both HSL3R452Y and HSL3L547Ymutants retained wild-type peptide-binding affinity (Fig. 3e), indicating that these residues specifically contribute to the receptor-co-receptor interaction surface rather than ligand recognition. These results validate the functional relevance of the zipper-like interface predicted by the model and highlight its role in stabilizing the active signaling complex.”

- The newly added SEC data (SI Fig 12) are very nice. They demonstrate nicely the molecular glue function of the peptide and formation of the HSL3–CTNIP4–SERK ternary complex. They also neatly distinguish the peptide binding mutant from the receptor-coreceptor interface mutant. This underlines the proposed structure of the ternary complex. A really nicely conducted experiment.

5. OUR RESPONSE: We thank the reviewer for this positive assessment of the ITC methodology and for appreciating the inclusion of individual experimental replicates. We agree that clear visualization of these data is important. Because of the large number of ITC datasets included in the figures, increasing their size substantially is unfortunately constrained by the overall figure layout and journal formatting limits.
To ensure that the data remain fully accessible and interpretable to the readers, we have provided all figures at very high resolution in the revised manuscript. This will allow readers to zoom into the ITC panels and inspect individual binding curves and fits in detail without loss of clarity. We hope that this approach is satisfactory, preserving the completeness of the dataset while maintaining a clear and readable figure layout that complies with the journal’s formatting requirements for both the main and supplementary figures.

- In the PDF I received the ITC data is still quite washed out, making it difficult to read also at high zoom. But I believe this is technical doe to PDF compression and I agree with the authors about the constraints put by layout and journal formatting. I am still very positive about including the replicates; how best to implement this so that the data are legible for readers I leave to the editorial staff as they might have more experience with what is good to do here.

6. OUR RESPONSE: We thank the reviewer for highlighting the need to provide a detailed description of the structure prediction procedure. As suggested, we have now added a dedicated section in the Method section describing the AlphaFold3 prediction workflow used to model the HSL3-CTNIP4-SERK complex. The relevant prediction confidence metrics are already reported in newly named Supplementary Figure 10. including iPTM, pTM, and PAE (included in the figure legend).
The new method section included reads: “AlphaFold modelling of the HSL3-CTNIP4-SERK3 ternary complex architecture. The HSL3-CTNIP4-SERK3 ternary complex was modelled using AlphaFold327 with the server implementation and default parameters. The prediction included the extracellular domains of HSL3 (residues 23-615) and BAK1/SERK3 (residues 26-200) together with the peptide CTNIP4 (residues 46-70). Predictions were generated using seed 1829186204. Model confidence was evaluated using ipTM, pTM and the predicted aligned error (PAE) between protein chains. “

- The reporting standards for predicted strucutres are still not established, and I thank the authors for detailing their approach in the Methods section and for reporting the relevant quality indicators. The proposed ternary complex is also backed up by solid experimental data now. I consider this therefore to be a well-conducted use of a prediction as a structural hypothesis that is then supported by a large set of experimental data in absence of the experimental ternary complex strucutre.

I could of course comment on the lack of multiple modeling runs (or how many models were generated), but given the lack of defined standards for this still emerging, but very powerful technique I am satisfied here.

7. OUR RESPONSE: We thank the reviewer for raising this insightful point and agree that understanding the mechanistic basis for the strong decrease in affinity caused by the additional N-terminal residue would be highly informative. Our structural data shows the peptide segment that is fully coordinated by the receptor, begins at residue Gln46. In the crystal structure, the backbone of Gln46 is coordinated (Fig 2d), and together with Arg47 occupies the entrance of the HSL3 binding pocket, forming a highly coordinated network of interactions that tightly anchor the peptide with the receptor. That fits very well with the binding data. Based on this structural framework, our working hypothesis (because we have no additional structural data with longer peptides) is that the presence of additional upstream residues, may interfere with the accessibility or positioning of these key anchoring residues, introducing steric clashes thereby reducing their ability to engage productively with the receptor pocket. In other words, extension of the N-terminus may partially mask or sterically hinder the presentation of the QR motif that is optimally positioned in the high-affinity peptide, resulting in the substantial decrease in affinity observed in our binding assays. In addition, we also show in our in vivo assays that the CTNIP4(46-70) peptide is highly bioactive in low nM concentration. Importantly, because the exact mature peptide boundaries in vivo remain unknown, we call the peptide CTNIP4(46-70) mature-like. Our data define the minimal receptor-coordinated segment required for high-affinity binding to HSL3.

- I thank the authors for this thorough analysis of their structural data and also for moderating the claim about the mature peptide. While I agree it is the likely interpretation, in absence of in vivo data about the mature peptide's actual boundaries, it would remain an overclaim to identify CTNIP4(46–70) as _the_ mature form. I am satisfied with the explanation and the changes.

8. OUR RESPONSE: We thank the reviewer for this thoughtful comment and for carefully considering the structural interpretation of the C-terminal region of CTNIP4. We agree that this region shows elevated ADPs in the crystal structure, indicating increased flexibility relative to the receptor-anchored N-terminal segment. As the reviewer correctly notes, the distal C-terminal residues are not strongly coordinated by HSL3 and appear less ordered in the structure. We also note that AlphaFold predictions do not explicitly account for solvent molecules, which could indeed mediate additional interactions not captured in the model and our biochemical assays. OU
Despite this apparent flexibility, our biochemical and functional data indicate that the cyclic C-terminal segment plays an essential role in receptor activation. Specifically, truncation of this region (CTNIP4(46–63)) or disruption of the disulfide bond (C58A/C68A) –which prevents formation of the cyclic loop– results in a dramatic reduction in binding affinity and strongly compromises peptide bioactivity in Ca^{2+} influx assays (new Supplementary Fig 3). These observations suggest that although the distal residues appear flexible in the crystal structure, the cyclic loop contributes to the formation of the hydrophobic surface required for productive receptor-co-receptor complex formation. Consistent with this interpretation, our thermodynamic analysis shows that SERK binding to the HSL3-CTNIP4 complex is strongly entropy-driven, supporting a mechanism in which hydrophobic interactions and potentially solvent displacement may contribute substantially to the interaction. To further test the predicted structural model, we performed CoIP and SEC biochemical analyses of receptor mutants targeting the predicted zipper-like receptor-co-receptor interface. Mutations within this interface (R452Y and L547Y), which lie at the center of the predicted interaction surface, abolish co-receptor association while preserving peptide binding affinity (New Fig. 3 and Supplementary Fig 12). These results strongly support the functional relevance of the predicted receptor-co-receptor interface.
Nevertheless, we agree with the reviewer that additional interactions-potentially including solvent-mediated contacts-may contribute to stabilizing the ternary complex and may not be fully captured in the predicted model.
To acknowledge this point, we have revised the Results and Discussion section to clarify that while the AlphaFold model provides a high-confidence structural hypothesis, additional polar interactions involving the flexible C-terminal region may contribute to stabilizing the receptor-co-receptor complex in vivo. Our discussion now reads, page 7-8 line 227-241”

- With the B-factor scale now labeled, and the additional 3σ Fo-Fc map provided, I am actually reassured here. I made my comment based on an 1.5σ map and unknown maximum B-factors from Fig 2. With the addtional data provided, I would say there is quite good confidence in this binding mode and combined with the additional biochemical assays I am satisfied with how this was clarified.

9. OUR RESPONSE:We thank the reviewer for this suggestion. we have revised Fig. 1a to improve clarity and readability. The panel has been enlarged to better visualize the color-coded representation of the peptide variants and their corresponding binding affinities. In addition, we have expanded the figure legend to more clearly explain the color scheme used in the panel.

- Readability of the first panel has been improved. While the content is fine, still the ITC figures are too small to really appreciate and maybe the figure panels would benefit from rearranging to make them more legible. As I am not an expert in formatting, I would leave it to the editor also here if this figure should be further rearranged.

Typos:

line 88: "N-ter anchoring" -> sounds a bit colloquial, maybe "N-terminal anchoring"
whole manuscript: mix of US/UK: signaling/signalling, stabilised/stabilized. Should be homogenised.

Reviewer #3

(Comments for the Author)
The authors have addressed most of my previous concerns, with one remaining issue unresolved.
While the comparison between the AlphaFold3-predicted ternary complex and the binary crystal structure (from this work) as

well as other existing ternary complexes (e.g., HSL1–IDL2–SERK1) to formulate hypotheses is acceptable, the mutational analysis serves as supportive evidence for complex formation rather than for the specific architecture (line 214).
Additionally, caution should be exercised when describing predicted structure comparisons with the term “reveal”, for example, in the phrase “reveal similar overall conserved complex architecture or distinct peptide-mediated activation mechanism” (lines 178–182). I recommend moderating the wording in such statements.
Finally, please revise the title of Figure 3. While it certainly “defines” a distinct phytocytokine recognition pattern, as supported by the binary crystal structure data, it does not define the receptor activation interface.

Open Access This Peer Review File is licensed under a Creative Commons Attribution 4.0 International License, which permits use, sharing, adaptation, distribution and reproduction in any medium or format, as long as you give appropriate credit to the original author(s) and the source, provide a link to the Creative Commons license, and indicate if changes were made.
In cases where reviewers are anonymous, credit should be given to 'Anonymous Referee' and the source.
The images or other third party material in this Peer Review File are included in the article’s Creative Commons license, unless indicated otherwise in a credit line to the material. If material is not included in the article’s Creative Commons license and your intended use is not permitted by statutory regulation or exceeds the permitted use, you will need to obtain permission directly from the copyright holder.
To view a copy of this license, visit https://creativecommons.org/licenses/by/4.0/

Point by point response: NPLANTS-251021254

OUR RESPONSE: We would like to thank the Reviewers for their positive and constructive feedback to improve our manuscript. Please, find below our response to the comments.

Response to Reviewer #1:

This paper reports the co-crystal structure analysis of the receptor kinase HSL3 and its ligand CTNIP4. The authors first identified CTNIP4(46–70) Cys-Cys as the minimal unit that exhibits high binding affinity for HSL3. They then performed co-crystal structural analysis of the HSL3–CTNIP4 complex and elucidated the structural details of the HSL3 ligand-binding pocket. By comparing the crystal structure of the HSL3–CTNIP4 complex with an AlphaFold3-predicted model of the HSL3–CTNIP4–SERK ternary complex, they further demonstrated that recruitment of the SERK co-receptor occurs through a hydrophobic interface generated upon peptide binding. A comparison with the phylogenetically related IDA–HAE co-crystal structure also revealed that subtle structural changes within the LRR-RK framework control ligand specificity. Collectively, the authors shed light on one aspect of the diverse mechanisms of ligand recognition by plant peptide hormone receptors

The series of results is supported by precise and solid co-crystal structural analyses, and I find no logical inconsistencies; the work is highly convincing. This work will likely be well received as a new successful example of co-crystallization studies of peptide ligand–receptor pairs.

OUR RESPONSE: We thank the reviewer for this thoughtful comment and for recognizing the robustness of the structural and biochemical analyses.

On the other hand, this reviewer is not fully convinced that the discovery of a hydrophobic pocket accommodating the disulfide-bond-stabilized cyclic CTNIP4 constitutes an impact strong enough to reshape established concepts of receptor–ligand recognition. A co-crystal structure of a disulfide-bond-stabilized cyclic peptide with an LRR protein has already been reported by the same authors for RALF4–LRX8 (Proc. Natl. Acad. Sci. USA 117, 7494–7503 (2020)). The loop region of RALF4, structurally stabilized by a disulfide bond, is essential for its high-affinity binding to LRX8. RALF4 peptide adopts a defined conformation when binding to the LRX LRR domain by making contacts with the binding pocket. Thus, it may be argued that the present study simply demonstrates that diverse receptor structures exist to accommodate diverse ligand architectures, rather than providing evidence for a fundamentally new concept in receptor–ligand recognition.

OUR RESPONSE: We thank the reviewer for this thoughtful comment and for highlighting the need to clarify the conceptual advance of our work. We agree that disulfide-stabilized peptide folds have previously been observed in ligand-protein complexes, including our earlier RALF4-LRX8 structure. However, the mechanistic role of the disulfide bond and the resulting mode of ligand recognition differ substantially between the two systems.

In the RALF4-LRX8 complex, the disulfide bond primarily stabilizes a pre-organized peptide fold required for high-affinity binding. The LRX protein functions as an extracellular scaffold that accommodates this folded peptide, and the disulfide bond mainly acts as a structural constraint that maintains ligand conformation. Consistent with this role, LRX proteins can recognize multiple folded RALF peptides and vice versa (Moussu *et al.*, 2020), indicating that the disulfide bond itself does not confer ligand specificity. In contrast, the HSL3-CTNIP4 structure reveals a distinct recognition principle. As shown in Supplementary Fig. 5, HSL3 contains a previously undescribed C-terminal ligand-binding pocket that differs markedly from those of other peptide-binding LRR receptor kinases, including HAE-IDL, BAM/CLV-CLE, MIK2-SCOOP, PEPR-PEP, and RGFR/RGI-RGF receptors. This pocket forms a hydrophobic cavity that specifically accommodates the cyclic CTNIP4 conformation. In this system, the disulfide bond does not simply stabilize the ligand but reshapes the peptide architecture to generate a hydrophobic surface that is directly recognized by the receptor pocket, thereby contributing to receptor-ligand specificity.

Importantly, this peptide-induced architecture also has functional consequences for receptor activation. The cyclic CTNIP4 fold positions hydrophobic residues such that peptide binding generates a receptor surface compatible with SERK co-receptor recruitment. Our extended biochemical and in vivo mutational analyses show that disrupting this interface strongly impairs co-receptor association and downstream signaling. Thus, in the HSL3 system the disulfide-stabilized peptide fold not only mediates receptor specificity but also contributes to organizing the receptor surface required for assembly of the active HSL3-SERK signaling complex.

We therefore think that our findings go beyond illustrating receptor structural diversity and instead reveal a novel mechanism by which ligand folding actively shapes receptor architecture to enable co-receptor recruitment and signaling activation. We have clarified this distinction in the revised Discussion section.

Response to Reviewer #2:

Summary:
The manuscript by Jiménez-Sandoval and coworkers provides a thorough structural and biochemical characterization of the HSL3–CTNIP4 receptor–ligand interaction. CTNIPs are peptides acting as phytocytokines and are bound by the HSL3/NUT LRR-RK, which induces heteromerization of the complex with SERK/BAK receptors. The authors redefine the minimal bioactive form of the CTNIP4 peptide as CTNIP4(46–70)Cys–Cys, which exhibits nanomolar affinity to its receptor and and shows robust signaling activity. The 2.12 Å crystal structure of HSL3-CTNIP4 reveals a unique recognition mechanism. CTNIP4 adopts a cyclic loop conformation that is recognized by an extended C-terminal hydrophobic pocket of HSL3, distinguishing it from other LRR-RKs that bind linear peptides. Functional assays (Ca^{2+} influx, MAPK activation) mirror ITC binding profiles and support the structural model.

Originality and Significance: The study is technically well conducted, relevant and interesting. The structure of the HSL3-CTNIP4 complex has not previously been determined and reveals a novel cyclic loop conformation, by which HSL3 binds CTNIP4 and ensures specificity for the ligand, preventing cross-activation with other LRR-RK. In addition, the study maps the optimal length for the bioactive CTNIP4.

Strengths:
- Mapping of affinity and functionality based on peptide length, revealing the peptide for optimal bioactivity
- High-resolution crystal structure of HSL3-CTNIP4 revealing a novel cyclic loop conformation that is critical for ligand binding
-Both biochemical and functional data, which strengthens the study.

Title, Abstract and introduction:
The title reflects the content of the manuscript. The abstract is well written and describes the presented study, as does the introduction. Relevant literature is cited (I focused on the structural biology refs). In general the language of the manuscript is clear, understandable and concise. I have pointed out the few typos below.

Data and Methodology:
Overall the experimental design and methodology is sound. I have a few detailed comments:

OUR RESPONSE: We thank the reviewer for the positive and constructive evaluation of our work and for recognizing the technical rigor and significance of the study. We appreciate the reviewer’s assessment that the structural, biochemical, and functional analyses collectively provide a compelling framework for understanding CTNIP4 recognition by HSL3.

Crystallography:
I was not able to inspect the model/structure factors directly, as 9T26 is not yet released on the Protein Data Bank (I guess it's in HPUB?). Based on the reported crystal data, the structure seems to be of good quality, but I have several comments for clarification. Processing is conservative but sound. However, crystallographic data quality reporting in Table 1 relies on Rmerge/Rsym, which is outdated and redundancy dependent (Karplus & Diederichs, 2015). Redundancy independent R values are preferred, (Rpim or Rmeas) but at a minimum, the half-set correlation CC1/2 has to be reported in Table 1. It would be good to include the wavelength as well.

OUR RESPONSE: We thank the reviewer for this helpful comment. The crystallographic data were originally reported following the template provided by *Nature Plants*. In response to the reviewer’s suggestion, we have now updated Supplementary Table 1 to include the additional recommended data-quality metrics.

The Fo–Fc omit map (Supplementary Figure 3) is shown at 1.5 sigma? Is this a typo/an oversight and is it the 2Fo–Fc map instead, for which the sigma cutoff would be more appropriate? This is a rather low sigma value for the Fo-Fc difference map which is more prone to showing noise. Factors like low resolution, partial occupancies and flexibility can indeed require that a lower sigma cutoff than the usual 3 sigma is chosen and appropriate, but for a high resolution 2.12 Å crystal structure it is a bit unusual. Please clarify. For a clearer representation of the omit map I would also consider to omit the receptor from the graphics and only show the density around the ligand itself. Another panel could then still show the receptor as well. Also if the receptor is shown, the sidechains shown in stick representation (I guess they are important for binding?) should be labeled.

OUR RESPONSE: We thank the reviewer for this constructive suggestion. The omit map shown corresponds to an |Fo–Fc| difference map contoured at 1.5σ, which we used to better visualize certain side-chain features of the peptide, particularly the arginine residues that participate in the interaction network. While we agree that higher contour levels are typically preferred for difference maps, the lower contour level was chosen here to allow clearer visualization of these features. To address the reviewer’s concern and improve clarity, we have now added an additional panel showing the omit map contoured at 3σ, which more strictly highlights the well-defined electron

density corresponding to the peptide. In addition, we have revised the figure to improve interpretability: the relevant receptor residues involved in peptide binding are now labeled, and the map representation has been adjusted to more clearly focus on the density surrounding the ligand. These changes are now included in the relabelled Supplementary Fig. 4.

Figure 2b: What is a "low" and what a "high" B-factor? Please provide B-factor values.

OUR RESPONSE: We thank the reviewer for this helpful comment. To clarify the interpretation of the B-factor distribution, we have now included the corresponding B-factor values directly in Fig. 2b.

Protein expression, purification, ITC assays: Protein expression and purification is described well and protein quality is shown reliably in Supplementary Figure 1. Given the high affinity of the HSL3-CTNIP4(46-70)-SERK interaction, can this complex be isolated on SEC, as done for other LRR-RK complexes?

OUR RESPONSE: We thank the reviewer for this constructive suggestion. Following this recommendation, we performed size-exclusion chromatography (SEC) experiments to assess the ternary complex formation between HSL3, CTNIP4 and the SERK co-receptor. We have now included these data as new Supplementary Fig. 12, which complements the biochemical and new in vivo analyses presented in Fig. 3.
The SEC profiles show that the HSL3-SERK ternary complex is formed in a peptide-dependent manner, consistent with the ITC measurements. Furthermore, analysis of receptor mutants demonstrates that disruption of CTNIP4 binding prevents ternary complex formation, whereas mutations affecting the zipper-like receptor-co-receptor interface abolish SERK association even in the presence of the peptide. These results further support our structural model and biochemical data and highlight the requirement for both peptide binding and the zipper interface to stabilize the active ternary complex. The manuscript has been updated accordingly, and the new data are described in the Results section that now reads: page 6-7 lines 196-207.“Consistent with the predicted ternary complex model, substitution of key HSL3 residues involved in peptide coordination or forming the receptor-co-receptor interface (Arg452Tyr and Leu547Tyr) abolished SERK1 association both in vitro and in vivo, supporting the proposed architecture of the ternary complex (Fig. 3a,e,f and Supplementary Figs. 1, 10 ,12 and 13). Importantly, both HSL3R452Y and HSL3L547Ymutants retained wild-type peptide-binding affinity (Fig. 3e), indicating that these residues specifically contribute to the receptor-co-receptor interaction surface rather than ligand recognition. These results validate the functional relevance of the zipper-like interface predicted by the model and highlight its role in stabilizing the active signaling complex.”

The ITC curves in the figures are too small. I appreciate the amount of ITC data and encourage very much to show individual replicates, but as it is now, one can hardly see them. The general ITC methodology is strong and convincing and well described in the method section.

OUR RESPONSE: We thank the reviewer for this positive assessment of the ITC methodology and for appreciating the inclusion of individual experimental replicates. We agree that clear visualization of these data is important. Because of the large number of ITC datasets included in the figures, increasing their size substantially is unfortunately constrained by the overall figure layout and journal formatting limits.
To ensure that the data remain fully accessible and interpretable to the readers, we have provided all figures at very high resolution in the revised manuscript. This will allow readers to zoom into the ITC panels and inspect individual binding curves and fits in detail without loss of clarity. We hope that this approach is satisfactory, preserving the completeness of the dataset while maintaining a clear and readable figure layout that complies with the journal’s formatting requirements for both the main and supplementary figures.

Structure prediction: The methodology for the deep learning structure prediction using Alphafold3 of the HSL3-CTNIP4-SERK complex is not present in the Materials & Methods sections. It should be included, even if "only" the server implementation of Alphafold 3 was used. Modeling parameters, number of models etc... should be provided. Predictions should be supported by their relevant quality indicators such as iPTM, pTM, pLDDT, PAE. This is especially important here, as a complex architecture is predicted.

OUR RESPONSE: We thank the reviewer for highlighting the need to provide a detailed description of the structure prediction procedure. As suggested, we have now added a dedicated section in the Method section

describing the AlphaFold3 prediction workflow used to model the HSL3-CTNIP4-SERK complex. The relevant prediction confidence metrics are already reported in newly named Supplementary Figure 10. including iPTM, pTM, and PAE (included in the figure legend).
The new method section included reads: "**AlphaFold modelling of the HSL3-CTNIP4-SERK3 ternary complex architecture.**

The HSL3-CTNIP4-SERK3 ternary complex was modelled using AlphaFold3[27] with the server implementation and default parameters. The prediction included the extracellular domains of HSL3 (residues 23-615) and BAK1/SERK3 (residues 26-200) together with the peptide CTNIP4 (residues 46-70). Predictions were generated using seed 1829186204. Model confidence was evaluated using ipTM, pTM and the predicted aligned error (PAE) between protein chains. "

"

Conclusions:
The conclusions support the data well. I have a few comments to strengthen the manuscript:
1: The manuscript shows that longer N-terminal CTNIP4 variants (45–70) bind markedly weaker than 46–70 and suggests that additional N-terminal residues hinder recognition. It is intriguing that affinity decreases by more than x5000 by addition of a single residue, L45. What is the structural mechanism behind this? It is clear that residues 46-48 are perceived very stringently by the receptor and could act as an additional anchor (Figs 1 & 3) and it makes sense that removing these comes at a cost of affinity. But the mechanism behind the addition of a single residue decreasing affinity would be very relevant. It would support the author's point that 46-70 is indeed the bioactive CTNIP if a structural feature on the receptor could be identified that could read this amino acid.

OUR RESPONSE: We thank the reviewer for raising this insightful point and agree that understanding the mechanistic basis for the strong decrease in affinity caused by the additional N-terminal residue would be highly informative. Our structural data shows the peptide segment that is fully coordinated by the receptor, begins at residue Gln46. In the crystal structure, the backbone of Gln46 is coordinated (Fig 2d), and together with Arg47 occupies the entrance of the HSL3 binding pocket, forming a highly coordinated network of interactions that tightly anchor the peptide with the receptor. That fits very well with the binding data. Based on this structural framework, our working hypothesis (because we have no additional structural data with longer peptides) is that the presence of additional upstream residues, may interfere with the accessibility or positioning of these key anchoring residues, introducing steric clashes thereby reducing their ability to engage productively with the receptor pocket. In other words, extension of the N-terminus may partially mask or sterically hinder the presentation of the QR motif that is optimally positioned in the high-affinity peptide, resulting in the substantial decrease in affinity observed in our binding assays. In addition, we also show in our in vivo assays that the CTNIP4(46-70) peptide is highly bioactive in low nM concentration. Importantly, because the exact mature peptide boundaries in vivo remain unknown, we call the peptide CTNIP4(46-70) mature-like. Our data define the minimal receptor-coordinated segment required for high-affinity binding to HSL3.

2: What would really strengthen this manuscript would be experimental structural data for the ternary complex (CTNIP–HSL3–SERK). This might not be technically feasible, but it would address a central question about the binding mode: what does SERK recognize? The AlphaFold model provides a plausible hypothesis, but still leaves some questions:

OUR RESPONSE: We thank the reviewer for this valuable suggestion and fully agree that an experimental structure of the CTNIP-HSL3-SERK ternary complex would provide the most direct insight into the co-receptor recognition mechanism. We attempted to obtain such a structure and carried out extensive crystallization trials using the ternary complex. Unfortunately, despite considerable efforts, these attempts were unsuccessful and did not yield crystals suitable for high-resolution structural determination. To address this limitation, we significantly expanded the experimental validation of the proposed model. While the AlphaFold prediction provides a high-confidence structural hypothesis (see Supplementary Fig. 10), we sought to test its key structural features through complementary biochemical and in vivo experiments. First, we analyzed receptor mutants targeting residues predicted to coordinate peptide binding and assessed their impact on signaling responses (new Supplementary Fig. 8). Second, we experimentally tested the receptor-co-receptor interface predicted by AlphaFold, which forms a zipper-like surface between HSL3 and the SERK co-receptor. Mutations disrupting this interface were examined, in addition of the already provided ITC assays in Fig 3e, using co-immunoprecipitation (CoIP) (Fig 3h), and size-exclusion chromatography (SEC) (Supplementary Fig. 12) analyses. Importantly, these mutations do not affect peptide binding affinity (Fig 3e), indicating that they specifically disrupt co-receptor association

rather than ligand recognition. In contrast, mutations affecting the peptide-binding pocket impair both ligand binding and downstream signaling (Supplemementary Fig. 8). Together, these experiments demonstrate that disrupting either peptide recognition or the predicted receptor-co-receptor interface strongly compromises SERK recruitment and CTNIP4-induced signaling. Thus, although we do not have a ternary crystal structure, the hope that the combination of high-confidence AlphaFold predictions (supported by confidence metrics reported in Supplementary Fig. 10) and extensive biochemical and in vivo validation provides independent experimental support for the proposed architecture of the ternary signaling complex. These additional data strengthen the mechanistic model derived from the HSL3-CTNIP4 crystal structure and the predicted HSL3-SERK interface.

The new experiments and analyses are now incorporated into the manuscript and presented in Fig. 3 and Supplementary Figs. 8, 10, 11 and 12. In addition, we have revised the Results and Discussion section to clearly frame the ternary complex architecture as a *structurally informed mechanistic model supported by functional validation,* rather than as a directly determined structure. The new rephrasing and data incorporation now reads:page 6 (line 164)"To investigate how CTNIP4 binding promotes receptor activation, we compared the crystal structure of the HSL3-CTNIP4 complex with AlphaFold3-predicted models of the ternary HSL3-CTNIP4-SERK3 assembly. The model displays high confidence, with very low inter-chain predicted aligned error (PAE) values ranging from 0.76 to 1.24 Å, supporting a reliable prediction of the receptor-co-receptor interfaces (Fig. 3a and Supplementary Fig. 10). Structural alignment further reveals that CTNIP4 adopts a nearly identical conformation in the binary and predicted ternary complexes (RMSD = 0.53 Å), suggesting that peptide binding to HSL3 likely pre-organizes the receptor surface for co-receptor recruitment (Fig. 3a and Supplementary Fig. 10)."

Page 6 (line 182)"Upon CTNIP4 binding, the receptor-peptide interface reorganises to generate a new predominantly hydrophobic surface along the C-terminal region of the peptide guided by the disulfide bond. In the AlphaFold-predicted ternary complex**,** this newly formed apolar patch seems to be directly engaging the N-terminal cap of the SERK co-receptor, acting as a molecular bridge that stabilises the ternary assembly (Fig. 3c and Supplementary Fig. 10). Supporting that hypothesis, ITC confirmed that while HSL3-CTNIP4 binding is enthalpy-driven, subsequent co-receptor recruitment is predominantly entropy-driven, consistent with the burial of this hydrophobic interface (Fig. 3c,d and Supplementary Fig. 10 and 11). Mutations disrupting the disulfide-stabilised cyclic fold or the local peptide region predicted to interact with SERKs (CTNIP4^{I61Y} and CTNIP4$^{N60A/F}$) markedly reduced co-receptor recruitment and overall binding affinity compared to the wild-type CTNIP4(46-70)$_{Cys\text{-}Cys}$ peptide (Fig. 3d and Supplementary Fig. 11). Similarly, truncated or linear variants lacking the cyclic loop (CTNIP4^{C58A}(46-63) and CTNIP4$^{C58A.C68A}$(46-70)) exhibited 20-60-fold weaker binding and a pronounced shift towards enthalpy-driven interactions, indicating loss of the entropy-favored interaction observed with the folded peptide. Together, these results highlight the importance of the disulfide-stabilized cyclic architecture**,** which generates the hydrophobic environment required for efficient SERK co-receptor recognition and receptor activation (Fig. 2d and 3c-d and Supplementary Figs. 10 and 11). Consistent with the predicted ternary complex model**,** substitution of key HSL3 residues involved in peptide coordination or forming the receptor-co-receptor interface **(**Arg452Tyr and Leu547Tyr**)** abolished SERK1 association both *in vitro* and *in vivo***,** supporting the proposed architecture of the ternary complex (Fig. 3a,e,f and Supplementary Figs. 1, 10 ,12 and 13). Importantly, both HSL3^{R452Y} and HSL3^{L547Y}mutants retained wild-type peptide-binding affinity (Fig. 3e)**,** indicating that these residues specifically contribute to the receptor-co-receptor interaction surface rather than ligand recognition. These results validate the functional relevance of the zipper-like interface predicted by the model and highlight its role in stabilizing the active signaling complex.

To translate these structural and thermodynamic findings into a physiological context, we assessed signaling competence of the receptor and peptide variants *in planta* using cytosolic Ca^{2+} influx and MAP kinase phosphorylation assays. CTNIP4 mutants that impaired either peptide-receptor or receptor-co-receptor interfaces exhibited a reduced Ca^{2+} influx or MAPK phosphorylation (Fig. 3g,h and Supplemental Fig. 8 and 13), mirroring their diminished binding affinities *in vitro* (Fig. 2e-f, 3d-e and Supplementary Figs. 6 and 10). These findings validate the proposed architecture of the complex and demonstrate that the disulfide-stabilized cyclic fold of CTNIP4 generates a hydrophobic interface that promotes SERK co-receptor recruitment through a zipper-like receptor-co-receptor interaction surface**,** enabling formation of the active ternary signaling complex and downstream activation."

Figure 3 states "Upon CTNIP4 binding, the receptor-peptide interface reorganises to generate a new predominantly hydrophobic surface along the C-terminal region of the peptide guided by the disulfide bond. This newly formed apolar patch directly engages the N-terminal cap of the SERK co-receptor, acting as a molecular bridge that stabilises the ternary assembly (Fig. 3c)." While this is a compelling observation, Figure 3C illustrating this surface needs improvement. The ITC dissection of receptor-ligand binding do show convincing the entropy vs enthalpy driven binding, so the conclusions are sound, but the current figure with the mesh is more confusing than illustrating. One could e.g. add an additional panel to show the electrostatic properties of the hydrophobic interaction surface.

OUR RESPONSE: We thank the reviewer for this helpful suggestion and for recognizing the thermodynamic evidence supporting the proposed interaction mechanism. To improve the visualization of the interaction surface, we have generated a new Supplementary Fig. 11, which complements Fig. 3c. This figure more clearly illustrates the chemical nature of the receptor-peptide-co-receptor interface, including the electrostatic and hydrophobic properties of the interacting surfaces. These representations better highlight the hydrophobic patch formed upon CTNIP4 binding to HSL3, which is predicted to engage the SERK co-receptor.
Importantly, this visualization aligns with our ITC thermodynamic analysis, which indicates that SERK binding to the HSL3-CTNIP4 complex is predominantly entropy-driven, consistent with hydrophobic interactions dominating the receptor-co-receptor interface. We hope that the addition of this supplementary figure improves the clarity of the structural interpretation supporting the main conclusions presented in Fig. 3.

Figure 2 suggests that the C-terminal part of CTNIP4 has high ADPs, is poorly ordered and not strongly coordinated by HSL3 (Figure 2d, a bit difficult to read). As shown in Fig. 3, this stretch is also hardly recognized by SERK (according to the AF model, Fig 2c. To me, there is a bit of a conceptual paradox: The presence of such a large interaction surface created by the cyclic loop and then the distal C-terminal residues being neither well ordered nor clearly utilized by receptor or co-receptor. Could the AF prediction be biased by training on existing linear peptide complexes deposited in the PDB? Deleting this stretch (63-70) has a large effect on affinity, how do individual amino acids contribute? Could there be more to this binding mechanism or could the ternary complex could look different from the AF prediction shown in Figure 3 as it looks like that SERK3 does hardly interact with those residues but mostly with the CTNIP motif?

OUR RESPONSE: We thank the reviewer for this thoughtful comment and for carefully considering the structural interpretation of the C-terminal region of CTNIP4. We agree that this region shows elevated ADPs in the crystal structure, indicating increased flexibility relative to the receptor-anchored N-terminal segment. As the reviewer correctly notes, the distal C-terminal residues are not strongly coordinated by HSL3 and appear less ordered in the structure. We also note that AlphaFold predictions do not explicitly account for solvent molecules, which could indeed mediate additional interactions not captured in the model and our biochemical assays.
Despite this apparent flexibility, our biochemical and functional data indicate that the cyclic C-terminal segment plays an essential role in receptor activation. Specifically, truncation of this region (CTNIP4(46–63)) or disruption of the disulfide bond (C58A/C68A) –which prevents formation of the cyclic loop– results in a dramatic reduction in binding affinity and strongly compromises peptide bioactivity in Ca^{2+} influx assays (new Supplementary Fig 3). These observations suggest that although the distal residues appear flexible in the crystal structure, the cyclic loop contributes to the formation of the hydrophobic surface required for productive receptor-co-receptor complex formation. Consistent with this interpretation, our thermodynamic analysis shows that SERK binding to the HSL3-CTNIP4 complex is strongly entropy-driven, supporting a mechanism in which hydrophobic interactions and potentially solvent displacement may contribute substantially to the interaction. To further test the predicted structural model, we performed CoIP and SEC biochemical analyses of receptor mutants targeting the predicted zipper-like receptor-co-receptor interface. Mutations within this interface (R452Y and L547Y), which lie at the center of the predicted interaction surface, abolish co-receptor association while preserving peptide binding affinity (New Fig. 3 and Supplementary Fig 12). These results strongly support the functional relevance of the predicted receptor-co-receptor interface.
Nevertheless, we agree with the reviewer that additional interactions-potentially including solvent-mediated contacts-may contribute to stabilizing the ternary complex and may not be fully captured in the predicted model. To acknowledge this point, we have revised the Results and Discussion section to clarify that while the AlphaFold model provides a high-confidence structural hypothesis, additional polar interactions involving the flexible C-terminal region may contribute to stabilizing the receptor-co-receptor complex in vivo. Our discussion now reads, page 7-8 line 227-241"A high-confidence AlphaFold3 prediction of the HSL3-CTNIP4-SERK ternary complex suggests that peptide binding reorganizes the receptor surface to generate a hydrophobic

platform that promotes SERK recruitment, further stabilized by a zipper-like receptor-co-receptor interface. Although additional polar or solvent-mediated contacts cannot be excluded, thermodynamic analyses indicate that SERK binding to the HSL3-CTNIP4 complex is predominantly entropy-driven, consistent with hydrophobic interactions dominating the interface. Accordingly, disruption of the cyclic fold or the hydrophobic interface abolishes ligand-induced interactions and signaling and shifts the interaction toward an enthalpy-driven signature, highlighting the role of the disulfide-stabilized conformation in generating the hydrophobic platform required for co-receptor engagement. Together, these findings show how the cyclic CTNIP4 fold couples ligand recognition to formation of a hydrophobic receptor surface that recruits the SERK co-receptor, providing a mechanistic framework for HSL3 activation and illustrating how conserved LRR scaffolds evolve distinct peptide recognition strategies to confer ligand specificity and orchestrate receptor-co-receptor assembly in plants."

Minor comments and typos: Typos:
Line 89: "Supplememtary" -> Supplementary – Thank you. Corrected.
Line 99: "stoichoimetry"- Thank you. Corrected
Line 113 "The peptide preorganized conformation facilitates"... -> "The peptide's preorganized conformation facilitates" – Thank you. Corrected.
Line 150: "Suplementary"- Thank you. Corrected.
Line 499: Ca+2 influx assays -> Ca^{2+} influx assays- Thank you. Corrected.

Figure 1: Panel A is not very intuitive and could benefit from reformatting. I can see that affinity of HSL3 to CTNIP4 finds an optimum around 46/47, with both shorter and longer peptides not being perceived with high affinity. But the color-coded panel is too small for that. Could the representation be improved along with the rest of the figure and include also the functional data, as they correlate quite well with the binding data?

OUR RESPONSE:We thank the reviewer for this suggestion. we have revised Fig. 1a to improve clarity and readability. The panel has been enlarged to better visualize the color-coded representation of the peptide variants and their corresponding binding affinities. In addition, we have expanded the figure legend to more clearly explain the color scheme used in the panel.

Supplementary Fig 5. What is shown in Panels a, b? Legend incomplete

OUR RESPONSE: We thank the reviewer for pointing out this lack of clarity. We have revised the renamed Supplementary Fig. 6 legend to clearly indicate which ITC profiles correspond to panels a and b, and to ensure that all datasets are properly described.

Supplementary Fig. 7. Legend missing for Panel d. The description for Panel c refers to Figure 3b, which is a structure figure, not ITC data. –

OUR RESPONSE : We thank the reviewer for pointing this out. We have corrected the figure legend.

Response to Reviewer #3:

This manuscript by Sandoval et al. reports the crystal structure of the Arabidopsis LRR receptor-like kinase HSL3 in complex with the disulfide-stabilized cyclic peptide ligand CTNIP4, complemented by biochemical and physiological analyses. The study elucidates how HSL3 specifically recognizes the phytocytokine CTNIP4, offering insights relevant to stomatal immunity and receptor-like kinase (RLK) research. Although the work presents compelling structural and functional data, several major concerns should be addressed.

OUR RESPONSE: We thank the reviewer for recognizing the strength of the structural and functional analyses presented in this study. We appreciate the reviewer's acknowledgment that our work provides mechanistic insight

into how the Arabidopsis receptor-like kinase HSL3 specifically recognizes the phytocytokine CTNIP4, and its relevance to receptor-like kinase signaling and plant immunity.

1. The current title resembles that of a review article and should be revised to more specifically reflect the key findings, including the protein names involved.

OUR RESPONSE: We thank the reviewer for this helpful suggestion. In response, we have revised the title to more specifically reflect the key findings of the study and to explicitly include the relevant protein names. At the same time, we retained the overall structure of the original title, which was considered appropriate by Reviewer 2.
The revised title now reads: **“Disulfide bond sculpts the CTNIP4 phytocytokine fold for recognition by the receptor HSL3.”**

2. While the crystal structure of the HSL3–CTNIP4 complex and the accompanying biochemical and physiological data are robust, the analysis of the ternary HSL3-CTNIP4-SERK3 complex based solely on AlphaFold-3 predictions remains highly speculative. Drawing conclusions about conformational changes, interaction surfaces, and key residues from a predicted model is not supported.

OUR RESPONSE: We thank the reviewer for raising this important point. Unfortunately, despite our multiple efforts we could not get crystals from the ternary complex. We agree that the AlphaFold3 prediction represents a structural model rather than an experimentally determined structure and therefore must be interpreted with appropriate caution and validated. A similar concern was raised by Reviewer 2, and we address this point in detail above. In response to these comments, we have revised the manuscript to clearly state that the HSL3-CTNIP4-SERK architecture presented is a predicted mechanistic model informed by AlphaFold, rather than a directly determined ternary complex structure.
Importantly, the AlphaFold prediction of the ternary complex is supported by high confidence metrics, including very low inter-chain predicted aligned error (PAE) values (0.76–1.24 Å), as well as strong interface confidence scores (iPTM and pTM), which are reported in Supplementary Fig. 10. These metrics indicate a high-confidence prediction of the receptor-co-receptor interface. Nevertheless, we agree that such predictions require experimental validation. To address this point, we significantly expanded the experimental validation of the proposed interface through additional biochemical and in vivo analyses.
We had already shown, by ITC measurements, that SERK co-receptors bind the HSL3-CTNIP4 complex with high affinity, revealing a strongly entropy-driven interaction, consistent with the predominantly hydrophobic receptor-co-receptor interface predicted by the model AlphaFold. Importantly, when we performed the same experiments using the linear CTNIP4 C58A,C68A peptide, which cannot form the disulfide-stabilized cyclic loop, the ITC thermodynamic profile changed markedly: binding affinity was substantially reduced and the interaction became enthalpy-driven. This shift in thermodynamic signature suggests that the Cys–Cys loop fold of CTNIP4 contributes to the formation of the hydrophobic interaction surface, thereby enhancing the affinity of the receptor-co-receptor interaction. These observations further support and validate the proposed model in which the cyclic architecture of CTNIP4 helps organize the hydrophobic interface required for efficient SERK recruitment. To clarify this mechanistic interpretation, we have now incorporated this point into the Results and Discussion section of the revised manuscript.

In addition, we have challenged with in vivo co-immunoprecipitation (CoIP) assays the predicted receptor-co-receptor “zipper-like” interface and tested its functional consequences. These mutations were analyzed using ITC, but we have added in the revised version of the manuscript co-immunoprecipitation (CoIP) (Fig 3f and Supplementary Fig 13), and size-exclusion chromatography (SEC) experiments (Supplementary Fig.12). Importantly, these mutations retain wild-type peptide-binding affinity but disrupt co-receptor association (Fig. 3e), indicating that they specifically affect the receptor-co-receptor interaction surface rather than ligand recognition.
Furthermore, the same interface mutations strongly impair CTNIP4-induced signaling in vivo, as measured by Ca^{2+} influx assays (new Supplementary Fig. 8), providing additional physiological validation of the predicted interface. Together, these complementary biochemical and functional experiments independently support the architecture of the ternary complex proposed by the AlphaFold model.

To reflect these points more clearly, we have revised the manuscript to explicitly state that the AlphaFold prediction serves as a structurally informed hypothesis, which we then tested experimentally through mutational, biochemical, and in vivo analyses. These additional validation experiments are now presented in Fig. 3 and Supplementary Figs. 8, 10 , 12 and 13, and the Results and Discussion sections have been updated accordingly.

The new data incorporated in the results and discussion section now reads: page 6-8"To investigate how CTNIP4 binding promotes receptor activation, we compared the crystal structure of the HSL3-CTNIP4 complex with AlphaFold3-predicted models of the ternary HSL3-CTNIP4-SERK3 assembly. The model displays high confidence, with very low inter-chain predicted aligned error (PAE) values ranging from 0.76 to 1.24 Å, supporting a reliable prediction of the receptor-co-receptor interfaces (Fig. 3a and Supplementary Fig. 10). Structural alignment further reveals that CTNIP4 adopts a nearly identical conformation in the binary and predicted ternary complexes (RMSD = 0.53 Å), suggesting that peptide binding to HSL3 likely pre-organizes the receptor surface for co-receptor recruitment (Fig. 3a and Supplementary Fig. 10). In the AlphaFold-predicted ternary complex, the C-terminal cyclic loop of CTNIP4 is positioned at the interface between HSL3 and the SERK co-receptor, where it contacts the N-capping domain of SERK and appears to act as a molecular bridge between the receptor and co-receptor proteins. The model further suggests that the ternary assembly is stabilised by a zipper-like interaction surface formed between HSL3 LRRs 15-21 and SERK LRRs 1-5, extending toward the SERK C-terminal cap domain and generating a continuous receptor-co-receptor interface (Fig. 3a and Supplementary Fig. 10). Structural comparison of the modelled HSL3-CTNIP4-SERK3 ternary complex with the experimentally determined HSL1-IDL2-SERK1 structure revealed a similar co-receptor positioning (RMSD = 1.14 Å) and overall conserved complex architecture, but a distinct peptide-mediated activation mechanism in HSL3 (Fig. 3b). Upon CTNIP4 binding, the receptor-peptide interface reorganises to generate a new predominantly hydrophobic surface along the C-terminal region of the peptide guided by the disulfide bond. In the AlphaFold-predicted ternary complex**,** this newly formed apolar patch seems to be directly engaging the N-terminal cap of the SERK co-receptor, acting as a molecular bridge that stabilises the ternary assembly (Fig. 3c and Supplementary Fig. 10). Supporting that hypothesis, ITC confirmed that while HSL3-CTNIP4 binding is enthalpy-driven, subsequent co-receptor recruitment is predominantly entropy-driven, consistent with the burial of this hydrophobic interface (Fig. 3c,d and Supplementary Fig. 10 and 11). Mutations disrupting the disulfide-stabilised cyclic fold or the local peptide region predicted to interact with SERKs (CTNIP4^{I61Y} and CTNIP4$^{N60A/F}$) markedly reduced co-receptor recruitment and overall binding affinity compared to the wild-type CTNIP4(46-70)$_{Cys\text{-}Cys}$ peptide (Fig. 3d and Supplementary Fig. 11). Similarly, truncated or linear variants lacking the cyclic loop (CTNIP4^{C58A}(46-63) and CTNIP4$^{C58A.C68A}$(46-70)) exhibited 20-60-fold weaker binding and a pronounced shift towards enthalphy-driven interactions, indicating loss of the entropy-favored interaction observed with the folded peptide. Together, these results highlight the importance of the disulfide-stabilized cyclic architecture**,** which generates the hydrophobic environment required for efficient SERK co-receptor recognition and receptor activation (Fig. 2d and 3c-d and Supplementary Figs. 10 and 11). Consistent with the predicted ternary complex model**,** substitution of key HSL3 residues involved in peptide coordination or forming the receptor-co-receptor interface **(**Arg452Tyr and Leu547Tyr**)** abolished SERK1 association both *in vitro* and *in vivo***,** supporting the proposed architecture of the ternary complex (Fig. 3a,e,f and Supplementary Figs. 1, 10 ,12 and 13). Importantly, both HSL3^{R452Y} and HSL3^{L547Y}mutants retained wild-type peptide-binding affinity (Fig. 3e)**,** indicating that these residues specifically contribute to the receptor-co-receptor interaction surface rather than ligand recognition. These results validate the functional relevance of the zipper-like interface predicted by the model and highlight its role in stabilizing the active signaling complex.

To translate these structural and thermodynamic findings into a physiological context, we assessed signaling competence of the receptor and peptide variants *in planta* using cytosolic Ca^{2+} influx and MAP kinase phosphorylation assays. CTNIP4 mutants that impaired either peptide-receptor or receptor-co-receptor interfaces exhibited a reduced Ca^{2+} influx or MAPK phosphorylation (Fig. 3g,h and Supplemental Fig. 8 and 13), mirroring their diminished binding affinities *in vitro* (Fig. 2e-f, 3d-e and Supplementary Figs. 6 and 10). These findings validate the proposed architecture of the complex and demonstrate that the disulfide-stabilized cyclic fold of CTNIP4 generates a hydrophobic interface that promotes SERK co-receptor recruitment through a zipper-like receptor-co-receptor interaction surface**,** enabling formation of the active ternary signaling complex and downstream activation."

Discussion now reads (page8):"…A high-confidence AlphaFold3 prediction of the HSL3-CTNIP4-SERK ternary complex suggests that peptide binding reorganizes the receptor surface to generate a hydrophobic platform that promotes SERK recruitment, further stabilized by a zipper-like receptor-co-receptor interface. Although additional

polar or solvent-mediated contacts cannot be excluded, thermodynamic analyses indicate that SERK binding to the HSL3-CTNIP4 complex is predominantly entropy-driven, consistent with hydrophobic interactions dominating the interface. Accordingly, disruption of the cyclic fold or the hydrophobic interface abolishes ligand-induced interactions and signaling and shifts the interaction toward an enthalpy-driven signature, highlighting the role of the disulfide-stabilized conformation in generating the hydrophobic platform required for co-receptor engagement. Together, these findings show how the cyclic CTNIP4 fold couples ligand recognition to formation of a hydrophobic receptor surface that recruits the SERK co-receptor, providing a mechanistic framework for HSL3 activation and illustrating how conserved LRR scaffolds evolve distinct peptide recognition strategies to confer ligand specificity and orchestrate receptor-co-receptor assembly in plants."

3. Several conclusions appear overstated. For instance, claims such as "uncover a previously unrecognized mechanism," "a previously uncharacterized cavity," and "employs a novel mechanism to perceive CTNIP/SCREW peptides" should be moderated or more precisely contextualized.

OUR RESPONSE: We thank the reviewer for this comment and appreciate the suggestion to carefully contextualize our conclusions. The statements referring to a "previously unrecognized mechanism" or a "previously uncharacterized cavity" are based on our comparative structural analyses across known LRR receptor–peptide complexes. In particular, Supplementary Fig. 5 highlights the distinct architecture of the C-terminal ligand-binding pocket in HSL3 compared with other structurally characterized LRR receptor kinases (including HAE-IDL, BAM/CLV-CLE, MIK2-SCOOP, PEPR-PEP and RGFR/RGI-RGF complexes, among others). This analysis shows that the hydrophobic cavity accommodating the cyclic CTNIP4 fold is not observed in previously described receptor-peptide structures. In addition, Supplementary Fig. 9 further illustrates how this structural feature contributes to the specific recognition of CTNIP peptides.
Nevertheless, we agree that careful wording is important. To address the reviewer's concern, we have revised the relevant passages in the manuscript to better contextualize these statements while still reflecting the structural evidence presented in Supplementary Figs. 5 and 9.

Specific points: •

Line 27: The statement "a feature absent in the closely related HAE/HSL LRR-RKs" is misleading without further clarification regarding HAE/HSL.

OUR RESPOSNE: We thank the reviewer for this clarification point. To better support this statement, we refer to the structural comparisons between HSL3 and HAE-like receptors, which are illustrated in Supplementary Fig. 5 and Supplementary Fig. 9, together with the corresponding PDB references. These figures highlight the structural differences in the ligand-binding pocket between HSL3 and the HAE/HSL receptor family. To improve clarity, we have revised the text to more explicitly reference these structural comparisons in the revised manuscript.

The revised text now reads (page 5, lines 150-162): "HSL3 displays an expanded C-terminal pocket that forms a distinct hydrophobic cavity shaped to accommodate the disulfide-locked cyclic architecture of CTNIP4. This structural extension generates a unique recognition surface that enables HSL3 to stabilise and coordinate the cyclic CTNIP4 fold, a feature absent from its sequence-related paralogues (Supplementary Fig. 5). From the ligand perspective, CTNIPs and IDA/IDL peptides share a broadly similar N-terminal and core region but diverge markedly at their C-terminal end. The additional residues and disulfide-stabilised loop in CTNIPs introduce a structural determinant that promotes selective docking into the HSL3 pocket, thereby preventing cross-recognition by other IDA-type receptors and *vice versa* (Supplementary Fig. 9)[4]. Together, these structural insights define HSL3 as a specialised receptor for cyclic CTNIP peptides, illustrating how subtle architectural variations within conserved LRR-RK frameworks can drive ligand specificity and define a new mechanism for peptide perception in plants."

• Line 30: The phrase "these structural, biochemical and physiological insights uncover a previously unrecognised mechanism of peptide perception and receptor activation" should be revised. Since most ligand–receptor interactions are unique, the authors are encouraged to specify the proteins involved, as suggested for the title.

OUR RESPONSE: We thank the reviewer for this helpful suggestion. In response, we have revised the sentence to explicitly refer to the proteins involved. The statement now specifies the HSL3 receptor and the CTNIP4 peptide, providing clearer context for the mechanism described in this study.
It now reads (page 1): "Together, these structural, biochemical and physiological insights uncover a previously unrecognised mechanism of CTNIP4 peptide perception and HSL3 receptor activation, highlighting how subtle architectural variations enable precise ligand selectivity among highly conserved plant receptor kinases."

"

• The structure determination method should be briefly mentioned in the main text, and the Ramachandran plot should be provided in the Table S1 to show the model quality.

OUR RESPONSE: We thank the reviewer for this helpful suggestion. The structure determination methodology is already described in the Materials and Methods section of the manuscript. To improve clarity, we have now added a reference to this section in the main text where the structure is first introduced.
In addition, we have included the Ramachandran statistics in Table S1, reporting the percentage of residues in favored and allowed regions, to further document the quality of the structural model.
The sentence to link to the method section now reads: (page 3, line 74-75)"Given the essential role of the conserved cysteine residues for receptor interaction[4], all variants were synthesised with a disulfide bond linking Cys58 and Cys68 to stabilise the folded conformation and peptide homogeneity (see Methods). "

• Line123 The N-terminal residues 46-47 of SCREW2, as well as residues involving binding in HSL3, should be tested through mutation, as these two residues seem very important for the high affinity binding

OUR RESPONSE: We thank the reviewer for this helpful suggestion. To assess the contribution of these N-terminal residues, we adopted an approach based on peptide truncation, which allowed us to evaluate not only the contribution of the side chains of residues 46-47 but also the role of the peptide backbone in receptor coordination. Binding assays using truncated peptide variants clearly demonstrate the importance of this N-terminal segment for high-affinity interaction with HSL3, consistent with the structural observations (data in Fig. 1b).
In addition, we introduced a receptor mutation in the HSL3 binding pocket (Q120A), which interacts with the backbone of this N-terminal region. This mutation significantly reduces peptide binding affinity, further illustrating the importance of the first residues of the peptide for proper anchoring and coordination within the receptor pocket. Together, the truncation experiments and receptor mutagenesis provide complementary evidence supporting the critical role of this N-terminal segment in peptide recognition by HSL3.

Line 128: The description "a previously uncharacterized cavity" requires clarification, has an HSL3 structure been reported before?

OUR RESPONSE: We thank the reviewer for this clarification point. To our knowledge, a structure of Arabidopsis HSL3 has not been previously reported by the time of the first submission of the article. Therefore, the cavity described in the manuscript corresponds to a structural feature revealed by the first crystal structure of HSL3 presented in this study.

• Lines 138–147: There appears to be a logical inconsistency in the description of the overall topology and binding pocket. SCREW2 and IDA peptides seem to bind HSL proteins in a similar region, with the N-terminal part aligned but the C-terminal part diverging (Fig. S6), could this represent a distinct peptide-binding mode (as suggested by Fig. 3b)?

OUR RESPONSE: We thank the reviewer for this insightful comment. Indeed, the point raised here reflects one of the key observations of our study. As shown by the structural comparisons, the N-terminal region of the peptide aligns similarly within the receptor-binding groove, whereas the C-terminal region adopts a distinct trajectory, resulting in a different mode of engagement within the receptor pocket. This divergence is consistent with the distinct C-terminal pocket architecture of HSL3, which accommodates the cyclic CTNIP4 fold, representing a novel and distinct peptide-binding mode.

• Lines 156–171: The use of an AlphaFold-3 predicted structure to analyze conformational changes and pinpoint interaction residues is speculative. While a rough model may be acceptable, detailed structural comparisons and interaction analyses (e.g., Fig. 3) should be avoided or significantly condensed. Moreover, mutational analyses focused largely on CTNIP4, with limited attention to SERK3 residues.

OUR RESPONSE: We thank the reviewer for this comment. We believe that this concern is similar to the one raised previously and by Reviewer 2, which we addressed in detail above. As noted there, we agree that the AlphaFold3-derived ternary complex represents a structural prediction rather than an experimentally determined structure, and we have revised the manuscript to clearly present it as a mechanistic model rather than a definitive structure.

Importantly, as highlighted above the AlphaFold model of the HSL3-CTNIP4-SERK complex is supported by high-confidence prediction metrics, including very low inter-chain predicted aligned error (PAE) values and strong interface confidence scores (iPTM/pTM), which are reported in Supplementary Fig. 10. While these metrics support the reliability of the predicted interface, we agree that experimental validation is required.
To address this point, we complemented the structural prediction with extensive biochemical and functional validation. Our ITC measurements show that SERK co-receptors bind the HSL3-CTNIP4 complex with high affinity and reveal a strongly entropy-driven interaction, consistent with the predominantly hydrophobic receptor-co-receptor interface predicted by the model. Notably, when the linear CTNIP4 C58A,C68A peptide is used, the interaction becomes enthalpy-driven and substantially weaker, indicating that the disulfide-stabilized cyclic fold contributes to the formation of the hydrophobic surface that promotes high-affinity co-receptor binding.
In addition, we experimentally tested the predicted receptor-co-receptor interface through mutational analysis of key residues forming the proposed zipper-like interaction surface. These mutants have been analyzed using ITC, co-immunoprecipitation (CoIP), and size-exclusion chromatography (SEC) assays. Importantly, these mutations retain wild-type peptide-binding affinity but disrupt co-receptor association, demonstrating that they specifically affect the receptor-co-receptor interaction surface rather than ligand binding. Consistently, these mutations also impair CTNIP4-induced signaling in vivo, providing physiological support for the proposed interface.
Together, these complementary biochemical and in vivo analyses provide independent experimental support for the architecture of the ternary complex suggested by the AlphaFold model. As stated above, to address the reviewer's concern, we have revised the Results and Discussion sections to emphasize that the AlphaFold structure serves as a structurally informed hypothesis that we experimentally validate, rather than a definitive structural determination.

• Figure 1: The ITC binding curves could be moved to a supplementary figure to improve clarity.

OUR RESPONSE: We thank the reviewer for this suggestion. However, we have chosen to retain the ITC binding curves in the main figure because they represent the primary experimental evidence supporting the binding interactions described in this section. To ensure clarity and accessibility, we have provided the figures at high resolution, allowing readers to zoom in and examine the thermograms and fitted curves in detail.

Corrections to the manuscript identified by the authors:

We would like to apologize to the Editor and the reviewers for two errors that we identified in the manuscript during the revision process. These issues have now been corrected and the corresponding experiments repeated to ensure accuracy.

First, during resequencing of the HSL3 mutant constructs, we discovered that the HSL3 R358A and HSL3 E360A mutants had been inadvertently exchanged. We therefore regenerated the correct viruses and repeated the corresponding experiments. New proteins were expressed and purified, and the binding experiments were repeated, resulting in updated ITC measurements, thermograms, SEC profiles, and SDS-PAGE analyses, which are now presented in Fig. 2f, Supplementary Fig 1 and Supplementary Fig. 6 . In addition, we tested the physiological impact of these corrected mutants ***in vivo***, and the results are now included in new Supplementary Fig. 8, showing full agreement with the updated biochemical data. The Results section and associated figures have been revised accordingly.

Second, we detected an error in Fig. 3d, where the Kd value for the SERK1 vs HSL3 + $\text{CTNIP4}_{\text{C58A}}$(46.63) experiment was inadvertently duplicated from the dataset corresponding to the HSL3-peptide interaction only. This value has now been corrected in the updated figure.

For transparency, we have attached the original ITC data table (also in the Supplementary material) corresponding to these measurements, which has now been corrected in **Fig. 3d**.

We sincerely apologize for these oversights and thank the Editor and reviewers for their understanding.

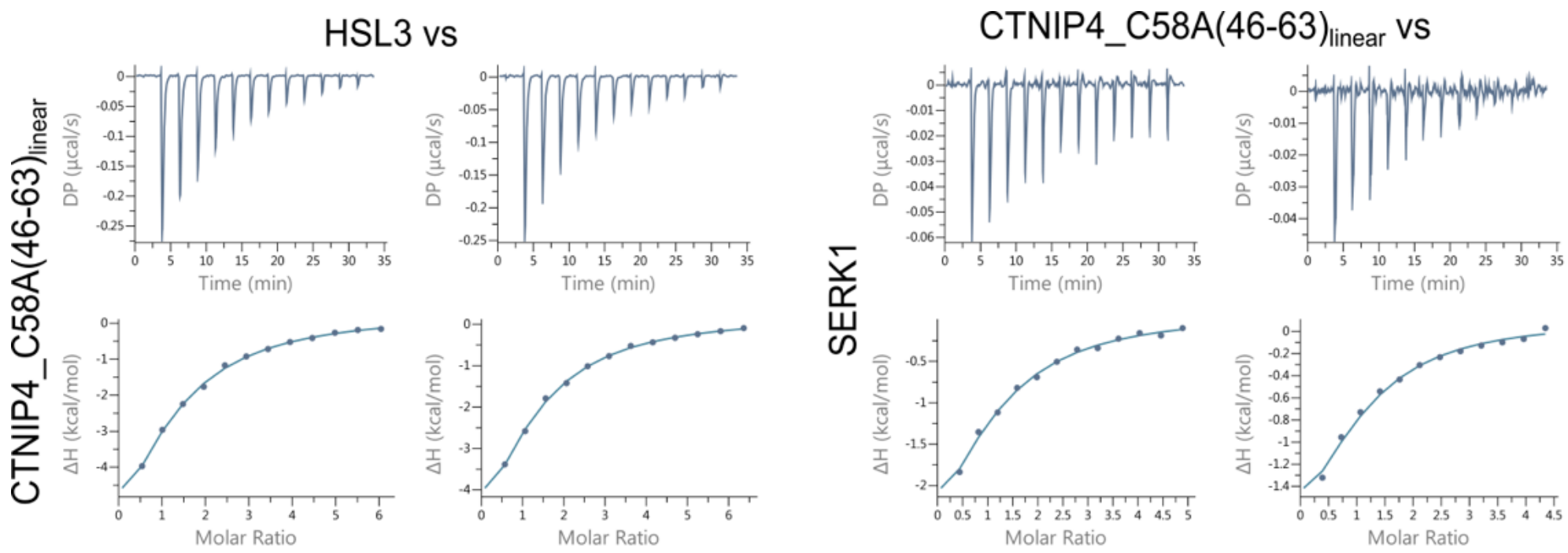

Ligand$_{(syringe)}$	Receptor$_{(cell)}$	$K_{d(nM)}$	$\Delta H_{(kcal/mol)}$	N
CTNIP4_C58A(46-63)$_{linear}$	HSL3	21500.0 ± 300.0	-11.1 ± 1.0	1
+ SERK1		8205.0 ± 855.0	-3.7 ± 0.9	1

*********** Reviewer Reports *************

Point by point response: NPLANTS-251021254

OUR RESPONSE: We would like to thank the Reviewers and the Editor for their positive and constructive feedback to improve our manuscript. Please, find below our response to the comments.

Response to Reviewer #1:

The LRR-RK subfamily XI functions as a class of receptors that recognize a wide variety of endogenous and exogenous peptide ligands. Because the extracellular LRR domain consists of relatively well-conserved repeating motifs, how ligand recognition specificity is achieved has long been a central question of interest to structural biologists. The elucidation of the crystal structure of the disulfide bond–containing peptide ligand CTNIP4 in complex with its receptor HSL3 provides a new example illustrating how subtle structural differences within the LRR domain can give rise to ligand selectivity.

Although some of the analyses rely on AlphaFold3-based predictions rather than experimental crystal structure data (perhaps reflecting current trends in the field), which represents a weakness of the study, the presented data as a whole are expected to contribute to a deeper understanding of peptide signaling.

OUR RESPONSE: We thank the reviewer for their thoughtful assessment of our work and for acknowledging its contribution to advancing our understanding of peptide signaling. We appreciate the reviewer's recognition that, taken together, the genetic, biochemical, structural, and functional data presented in this study provide important insights into the mechanisms of peptide perception by HSL3.

The authors' response to this reviewer's comment is largely limited to an expansion of the Discussion section; however, in the revised manuscript, the conceptual advance of their study is described more concretely, which should help readers better understand the work.However, the current version of the Discussion section leaves the reader with a rather dry impression. A more in-depth discussion—for example, regarding what biological advantages the newly identified ligand recognition mechanism may confer (i.e., why it has been maintained under diverse evolutionary selection pressures), as well as similarities and differences with ligand recognition mechanisms in receptors from other species—would further strengthen the manuscript.

OUR RESPONSE: We thank the reviewer for this thoughtful suggestion. To address this point, we have expanded the Discussion to further emphasize the evolutionary and biological implications of the HSL3 recognition mechanism. In particular, we discuss how the specialized HSL3 ligand-binding pocket, compared with the HAE–IDA receptor system, illustrates how structural diversification within a conserved LRR-RK scaffold can promote signaling specificity, prevent receptor cross-recognition, and facilitate the evolution of new peptide signaling pathways. A broader comparative analysis of HSL3 receptors and CTNIP peptides across plant species is currently the subject of ongoing work and will be presented in a separate study.

The new added paragraph in the discussion now reads: (lines 228-235):"The HSL3 ligand-binding pocket differs markedly from those of other peptide-sensing LRR-RKs[6,7], including the closely related HAE-HSL receptors. Whereas HAE-HSL receptors recognize the largely linear IDA/IDL peptides through a pocket that narrows towards the peptide C terminus, HSL3 has evolved an expanded hydrophobic cavity that specifically accommodates the disulfide-constrained cyclic architecture of CTNIP peptides. This structural innovation creates a distinct recognition surface that enables selective CTNIP perception while preventing cross-recognition by IDA/IDL receptors. More broadly, our findings illustrate how relatively subtle architectural modifications within a conserved LRR-RK scaffold can generate fundamentally different modes of peptide recognition, providing an evolutionary mechanism to expand peptide signaling repertoires while preserving signaling specificity. "

--**Response to Reviewer #2:**

Overall, I find that the revised manuscript has improved in response to the reviews. The authors have addressed my main technical concerns about crystallographic data reporting, electron density visualisation, B-factors, and the documentation of structural predictions. Importantly, they have added new experimental support for the proposed SERK-binding interface. Several earlier over-statements (e.g. regarding the "mature" peptide) were

moderated and now the ternary complex is correctly presented as a prediction that is constrained by experiments rather than having the confidence level of an experimental structure. My remaining comments below are mostly clarifications and minor points about data presentation, not additional experiments.

1: OUR RESPONSE: We thank the reviewer for this helpful comment. The crystallographic data were originally reported following the template provided by Nature Plants. In response to the reviewer's suggestion, we have now updated Supplementary Table 1 to include the additional recommended data-quality metrics.

- The crystallographic data reporting is according to the state-of-the-art now, thanks.

OUR RESPONSE: We thank the reviewer for this positive assessment and are pleased that the revised crystallographic data reporting now meets the expected standards.

2. OUR RESPONSE: We thank the reviewer for this constructive suggestion. The omit map shown corresponds to an |Fo–Fc| difference map contoured at 1.5σ, which we used to better visualize certain side-chain features of the peptide, particularly the arginine residues that participate in the interaction network. While we agree that higher contour levels are typically preferred for difference maps, the lower contour level was chosen here to allow clearer visualization of these features. To address the reviewer's concern and improve clarity, we have now added an additional panel showing the omit map contoured at 3σ, which more strictly highlights the well-defined electron density corresponding to the peptide. In addition, we have revised the figure to improve interpretability: the relevant receptor residues involved in peptide binding are now labeled, and the map representation has been adjusted to more clearly focus on the density surrounding the ligand. These changes are now included in the relabelled Supplementary Fig. 4.

- I thank the authors for including the 3σ Fo−Fc omit map and for improving the labelling of the ligand and receptor residues. At 2.1 Å resolution, the peptide backbone and most side chains are already well supported at 3σ (consistent with Fig. 2 B factors).

OUR RESPONSE: We thank the reviewer for this positive assessment and are pleased that the additional omit map and the improved labelling of the ligand and receptor residues have addressed the reviewer's concerns and enhanced the clarity of the structural data presentation.

If I understood it correctly, the long arginine side chains and the high-B regions participate in the predicted SERK interface. I would therefore in fact expect that they appear weaker or partially disordered in the binary HSL3–CTNIP4 complex.
Without the co-receptor, these side chains are partially exposed, have elevated B-factors and correspondingly look patchy in the omit map. This is exactly what the 3σ map shows and it is fully consistent with the proposed mechanism. In contrast, the 1.5σ map contains noise and, while it is visually more "complete", is not necessary to support the structural conclusions. It may encourage over-interpretation of poorly ordered side chains. I would therefore consider the 3σ panel completely sufficient; I am satisfied with the structural evidence without the lower contouring map.

3. OUR RESPONSE: We thank the reviewer for this helpful comment. To clarify the interpretation of the B-factor distribution, we have now included the corresponding B-factor values directly in Fig. 2b.

- Thanks, the labeling of the scale is sufficient and evident for at even the high-B region are still quite well defined (73.85 as max B).

OUR RESPONSE: We thank the reviewer for this positive assessment and are pleased that the revised figure presentation and labeling clearly convey the quality of the electron density, including in regions with higher B-factors.

4. OUR RESPONSE: We thank the reviewer for this constructive suggestion. Following this recommendation, we performed size-exclusion chromatography (SEC) experiments to assess the ternary complex formation between HSL3, CTNIP4 and the SERK co-receptor. We have now included these data as new Supplementary Fig. 12, which complements the biochemical and new in vivo analyses presented in Fig. 3.
The SEC profiles show that the HSL3-SERK ternary complex is formed in a peptide-dependent manner, consistent with the ITC measurements. Furthermore, analysis of receptor mutants demonstrates that disruption of CTNIP4 binding prevents ternary complex formation, whereas mutations affecting the zipper-like receptor-co-receptor interface abolish SERK association even in the presence of the peptide. These results further support our structural model and biochemical data and highlight the requirement for both peptide binding and the zipper interface to stabilize the active ternary complex. The manuscript has been updated accordingly, and the new data are described in the Results section that now reads: page 6-7 lines 196-207."Consistent with the predicted ternary complex model, substitution of key HSL3 residues involved in peptide coordination or forming the receptor-co-receptor interface (Arg452Tyr and Leu547Tyr) abolished SERK1 association both in vitro and in vivo, supporting the proposed architecture of the ternary complex (Fig. 3a,e,f and Supplementary Figs. 1, 10 ,12 and 13). Importantly, both HSL3R452Y and HSL3L547Ymutants retained wild-type peptide-binding affinity (Fig. 3e),

indicating that these residues specifically contribute to the receptor-co-receptor interaction surface rather than ligand recognition. These results validate the functional relevance of the zipper-like interface predicted by the model and highlight its role in stabilizing the active signaling complex."

- The newly added SEC data (SI Fig 12) are very nice. They demonstrate nicely the molecular glue function of the peptide and formation of the HSL3–CTNIP4–SERK ternary complex. They also neatly distinguish the peptide binding mutant from the receptor-coreceptor interface mutant. This underlines the proposed structure of the ternary complex. A really nicely conducted experiment.

OUR RESPONSE: We sincerely thank the reviewer for this very positive assessment. We are pleased that the additional SEC experiments clearly support the proposed molecular glue function of CTNIP4, the formation of the HSL3–CTNIP4–SERK ternary complex, and the distinct roles of the peptide-binding and receptor–co-receptor interface mutants. We appreciate the reviewer's recognition of the value of these experiments in supporting our structural model.

5. OUR RESPONSE: We thank the reviewer for this positive assessment of the ITC methodology and for appreciating the inclusion of individual experimental replicates. We agree that clear visualization of these data is important. Because of the large number of ITC datasets included in the figures, increasing their size substantially is unfortunately constrained by the overall figure layout and journal formatting limits. To ensure that the data remain fully accessible and interpretable to the readers, we have provided all figures at very high resolution in the revised manuscript. This will allow readers to zoom into the ITC panels and inspect individual binding curves and fits in detail without loss of clarity. We hope that this approach is satisfactory, preserving the completeness of the dataset while maintaining a clear and readable figure layout that complies with the journal's formatting requirements for both the main and supplementary figures.

- In the PDF I received the ITC data is still quite washed out, making it difficult to read also at high zoom. But I believe this is technical doe to PDF compression and I agree with the authors about the constraints put by layout and journal formatting. I am still very positive about including the replicates; how best to implement this so that the data are legible for readers I leave to the editorial staff as they might have more experience with what is good to do here.

OUR RESPONSE: We thank the reviewer for this constructive comment and for supporting the inclusion of the replicate ITC experiments. To preserve consistency with the journal layout, we have retained the current figure format. However, to ensure full accessibility of the data, all raw ITC files have been deposited and are available to the readers. We have also provided the figures at the highest possible resolution so that readers can enlarge the thermograms and inspect the individual traces in detail.

6. OUR RESPONSE: We thank the reviewer for highlighting the need to provide a detailed description of the structure prediction procedure. As suggested, we have now added a dedicated section in the Method section describing the AlphaFold3 prediction workflow used to model the HSL3-CTNIP4-SERK complex. The relevant prediction confidence metrics are already reported in newly named Supplementary Figure 10. including iPTM, pTM, and PAE (included in the figure legend).
The new method section included reads: "AlphaFold modelling of the HSL3-CTNIP4-SERK3 ternary complex architecture.
The HSL3-CTNIP4-SERK3 ternary complex was modelled using AlphaFold327 with the server implementation and default parameters. The prediction included the extracellular domains of HSL3 (residues 23-615) and BAK1/SERK3 (residues 26-200) together with the peptide CTNIP4 (residues 46-70). Predictions were generated using seed 1829186204. Model confidence was evaluated using ipTM, pTM and the predicted aligned error (PAE) between protein chains. "

- The reporting standards for predicted strucutres are still not established, and I thank the authors for detailing their approach in the Methods section and for reporting the relevant quality indicators. The proposed ternary complex is also backed up by solid experimental data now. I consider this therefore to be a well-conducted use of a prediction as a structural hypothesis that is then supported by a large set of experimental data in absence of the experimental ternary complex strucutre.I could of course comment on the lack of multiple modeling runs (or how many models were generated), but given the lack of defined standards for this still emerging, but very powerful technique I am satisfied here.
OUR RESPONSE: We sincerely thank the reviewer for this thoughtful and positive assessment. We are pleased that the additional methodological details and reporting of the prediction quality metrics, together with the extensive experimental validation presented in the revised manuscript, provide sufficient support for the proposed ternary complex model. We appreciate the reviewer's recognition of our approach of using the AlphaFold3 prediction as a structural hypothesis that is rigorously supported by complementary biochemical, biophysical, and functional data.

7. OUR RESPONSE: We thank the reviewer for raising this insightful point and agree that understanding the mechanistic basis for the strong decrease in affinity caused by the additional N-terminal residue would be highly informative. Our structural data shows the peptide segment that is fully coordinated by the receptor, begins at

residue Gln46. In the crystal structure, the backbone of Gln46 is coordinated (Fig 2d), and together with Arg47 occupies the entrance of the HSL3 binding pocket, forming a highly coordinated network of interactions that tightly anchor the peptide with the receptor. That fits very well with the binding data. Based on this structural framework, our working hypothesis (because we have no additional structural data with longer peptides) is that the presence of additional upstream residues, may interfere with the accessibility or positioning of these key anchoring residues, introducing steric clashes thereby reducing their ability to engage productively with the receptor pocket. In other words, extension of the N-terminus may partially mask or sterically hinder the presentation of the QR motif that is optimally positioned in the high-affinity peptide, resulting in the substantial decrease in affinity observed in our binding assays. In addition, we also show in our in vivo assays that the CTNIP4(46-70) peptide is highly bioactive in low nM concentration. Importantly, because the exact mature peptide boundaries in vivo remain unknown, we call the peptide CTNIP4(46-70) mature-like. Our data define the minimal receptor-coordinated segment required for high-affinity binding to HSL3.

- I thank the authors for this thorough analysis of their structural data and also for moderating the claim about the mature peptide. While I agree it is the likely interpretation, in absence of in vivo data about the mature peptide's actual boundaries, it would remain an overclaim to identify CTNIP4(46–70) as _the_ mature form. I am satisfied with the explanation and the changes.

OUR RESPONSE: We sincerely thank the reviewer for this positive assessment. We are pleased that the revised text and moderated interpretation have addressed the reviewer's concern.

8. OUR RESPONSE: We thank the reviewer for this thoughtful comment and for carefully considering the structural interpretation of the C-terminal region of CTNIP4. We agree that this region shows elevated ADPs in the crystal structure, indicating increased flexibility relative to the receptor-anchored N-terminal segment. As the reviewer correctly notes, the distal C-terminal residues are not strongly coordinated by HSL3 and appear less ordered in the structure. We also note that AlphaFold predictions do not explicitly account for solvent molecules, which could indeed mediate additional interactions not captured in the model and our biochemical assays. OU Despite this apparent flexibility, our biochemical and functional data indicate that the cyclic C-terminal segment plays an essential role in receptor activation. Specifically, truncation of this region (CTNIP4(46–63)) or disruption of the disulfide bond (C58A/C68A) –which prevents formation of the cyclic loop– results in a dramatic reduction in binding affinity and strongly compromises peptide bioactivity in Ca^{2+} influx assays (new Supplementary Fig 3). These observations suggest that although the distal residues appear flexible in the crystal structure, the cyclic loop contributes to the formation of the hydrophobic surface required for productive receptor-co-receptor complex formation. Consistent with this interpretation, our thermodynamic analysis shows that SERK binding to the HSL3-CTNIP4 complex is strongly entropy-driven, supporting a mechanism in which hydrophobic interactions and potentially solvent displacement may contribute substantially to the interaction. To further test the predicted structural model, we performed CoIP and SEC biochemical analyses of receptor mutants targeting the predicted zipper-like receptor-co-receptor interface. Mutations within this interface (R452Y and L547Y), which lie at the center of the predicted interaction surface, abolish co-receptor association while preserving peptide binding affinity (New Fig. 3 and Supplementary Fig 12). These results strongly support the functional relevance of the predicted receptor-co-receptor interface.
Nevertheless, we agree with the reviewer that additional interactions-potentially including solvent-mediated contacts-may contribute to stabilizing the ternary complex and may not be fully captured in the predicted model. To acknowledge this point, we have revised the Results and Discussion section to clarify that while the AlphaFold model provides a high-confidence structural hypothesis, additional polar interactions involving the flexible C-terminal region may contribute to stabilizing the receptor-co-receptor complex in vivo. Our discussion now reads, page 7-8 line 227-241”

- With the B-factor scale now labeled, and the additional 3σ Fo-Fc map provided, I am actually reassured here. I made my comment based on an 1.5σ map and unknown maximum B-factors from Fig 2. With the addtional data provided, I would say there is quite good confidence in this binding mode and combined with the additional biochemical assays I am satisfied with how this was clarified.

OUR RESPONSE: We sincerely thank the reviewer for this positive assessment. We are pleased that the inclusion of the labeled B-factor scale, the additional 3σ Fo−Fc omit map, and the complementary biochemical validation have clarified the structural interpretation and provided confidence in the proposed binding mode.

9. OUR RESPONSE:We thank the reviewer for this suggestion. we have revised Fig. 1a to improve clarity and readability. The panel has been enlarged to better visualize the color-coded representation of the peptide variants and their corresponding binding affinities. In addition, we have expanded the figure legend to more clearly explain the color scheme used in the panel.

- Readability of the first panel has been improved. While the content is fine, still the ITC figures are too small to really appreciate and maybe the figure panels would benefit from rearranging to make them more legible. As I am not an expert in formatting, I would leave it to the editor also here if this figure should be further rearranged.

OUR RESPONSE: We thank the reviewer for this constructive suggestion and are pleased that the readability of the first panel has improved. To maximize the legibility of the ITC data while maintaining the journal's figure layout, we have provided the figures at the highest possible resolution, allowing readers to zoom in and inspect the thermograms in detail.

Typos:

line 88: "N-ter anchoring" -> sounds a bit colloquial, maybe "N-terminal anchoring"
OUR RESPONSE: corrected
whole manuscript: mix of US/UK: signaling/signalling, stabilised/stabilized. Should be homogenised.
OUR RESPONSE: corrected

--**Response to Reviewer #3:**
The authors have addressed most of my previous concerns, with one remaining issue unresolved.
While the comparison between the AlphaFold3-predicted ternary complex and the binary crystal structure (from this work) as well as other existing ternary complexes (e.g., HSL1–IDL2–SERK1) to formulate hypotheses is acceptable, the mutational analysis serves as supportive evidence for complex formation rather than for the specific architecture (line 214). Additionally, caution should be exercised when describing predicted structure comparisons with the term "reveal", for example, in the phrase "reveal similar overall conserved complex architecture or distinct peptide-mediated activation mechanism" (lines 178–182). I recommend moderating the wording in such statements.
Finally, please revise the title of Figure 3. While it certainly "defines" a distinct phytocytokine recognition pattern, as supported by the binary crystal structure data, it does not define the receptor activation interface.

OUR RESPONSE: We thank the reviewer for this thoughtful comment. We agree that, while the mutational, biochemical and structural data strongly support the proposed model, they do not experimentally establish the precise architecture of the predicted ternary complex. Accordingly, we have moderated the wording throughout the manuscript to clearly distinguish experimentally determined observations from hypotheses derived from the AlphaFold3 model. In addition, we have revised the title of Figure 3 to avoid implying that the receptor activation interface is experimentally defined.

Line 214: We have reformulated that paragraph that now reads: "To translate these structural and thermodynamic findings into a physiological context, we assessed signalling competence of the receptor and peptide variants *in planta* using cytosolic Ca^{2+} influx and MAP kinase phosphorylation assays. CTNIP4 mutants that impaired either peptide-receptor or receptor-co-receptor interfaces exhibited a reduced Ca^{2+} influx or MAPK phosphorylation (Fig. 3g,h and Supplemental Fig. 8 and 13), consistent with their reduced binding affinities measured *in vitro* (Fig. 2e-f, 3d-e and Supplementary Figs. 6 and 10). Together, these findings provide functional support for the proposed ternary complex model and are consistent with a mechanism in which the disulfide-stabilized cyclic fold of CTNIP4 promotes SERK co-receptor recruitment by generating the hydrophobic interaction surface predicted by the AlphaFold3 model, thereby facilitating assembly of the active signalling complex and downstream signalling."

Regarding the phrase "in the phrase "reveal similar overall conserved complex architecture or distinct peptide-mediated activation mechanism" (lines 178–182). I recommend moderating the wording in such statements." Our new sentence now reads: "Structural comparison of the AlphaFold3-predicted HSL3–CTNIP4–SERK3 ternary complex with the experimentally determined HSL1–IDL2–SERK1 structure suggested a similar co-receptor positioning (RMSD = 1.14 Å) and an overall conserved complex architecture, while predicting a distinct peptide-mediated activation mechanism for HSL3 (Fig. 3b)"

Title of Figure 3 now reads: **Structural basis of HSL3 phytocytokine recognition and a model for receptor activation**
